# Supplementary material for: Potential traumatic events in the workplace and depression, anxiety and post-traumatic stress: a cross-sectional study among Dutch gynaecologists, paediatricians and orthopaedic surgeons
Source: BMJ Open. 2020 Sep 2;10(9):e033816. doi: 10.1136/bmjopen-2019-033816 (PMC7470507; doi:10.1136/bmjopen-2019-033816)
Supplement: Supplementary data [file bmjopen-2019-033816supp001.pdf]

Supplementary file 1: Questionnaires

Paediatricians

1 What is your gender?
1. Male
2. Female

2 What is your age?
1. 20-29 years
2. 30-39 years
3. 40-49 years
4. 50-59 years
5. 60-69 years
6. ≥ 70 years

3 Are you a member of the Dutch Society of Paediatricians (NvK)?
1. Yes
2. No

4 What is your current position?
1. Resident
2. Attending physician
3. Retired
4. Non-practicing/management

5 How many years have you been working in the paediatrics field as a physician? (Including as intern)
1. ≤ 5 years
2. 6-10 years
3. 11-15 years
4. 16-20 years
5. 21-25 years
6. 26-30 years
7. > 30 years

6 At the moment, are you working in a general hospital or in an academic referral centre?
1. General hospital
2. Academic referral centre
3. Both
4. I do not work anymore, but I used to work in a general hospital
5. I do not work anymore, but I used to work in an academic referral centre

7 At which department do you work the most? (If you are not practicing anymore, fill in where you used to work)
1. Neonatology
2. Emergency room
3. General paediatrician
4. NICU
5. Child intensive care
6. Policlinic
7. Children's oncology
8. Other…

8 What do you consider to be an adverse event on the work-floor? (More than one answer possible)
1. Not applicable

2. Bad news conversation/interview
3. (Critically) ill neonate
4. (Critically) ill children
5. When a patient dies
6. When you miss a diagnosis
7. When you have to refuse a patient
8. When you are in doubt whether you are making the right decision
9. When an inexperienced colleague is on call
10. When you decide on an abstaining course of treatment
11. Treatment of neonates
12. When you know a patient will remain severely handicapped
13. Handling the emotions of the parents
14. (Suspicion of) child abuse
15. Other …

9 How do you cope with adverse events on the work-floor? (More than one answer possible)
1. Not applicable / never experienced
2. Seeking professional help
3. Use new medication
4. Drink (more) alcohol
5. Smoke (more) cigarettes
6. Use (more) drugs
7. Work out (more)
8. Going home as soon as possible
9. Call in sick
10. Hide your emotions
11. Find a distraction
12. Praying or other religious activities
13. Talking to friends and/or family
14. Informally discussing the matter with peers/colleagues
15. Quitting
16. Work less
17. Not working any evening/night shifts
18. Other …

10 The current support organized by your institution after an adverse event is good:
1. Strongly disagree
2. Disagree
3. Agree
4. Strongly agree

11 There is plenty of room to informally discuss adverse events in the department/partnership:
1. Strongly disagree
2. Disagree
3. Agree
4. Strongly agree

12 There is plenty of room to informally discuss adverse events in the hospital:
1. Strongly disagree
2. Disagree
3. Agree
4. Strongly agree

13 There should be a culture change regarding support after an adverse event:
1. Strongly disagree

2. Disagree
3. Agree
4. Strongly agree

14 There is a protocol at your institution regarding support after an adverse event:
1. Yes
2. Yes, but nobody uses it
3. No, but there is one currently being created
4. No
5. I do not know

15 Support after an adverse event consisted of: (more than one answer possible)
1. I have never experienced an adverse event
2. There was none
3. Professionally organised peer-support
4. Self-initiated peer-support with direct colleagues (own department)
5. Self-initiated peer-support with indirect colleagues (different department)
6. Conversation(s) with a psychologist or coach
7. Evaluation with the present team
8. Help from the medical officer after a sick-leave
9. Other …

16 Your preferred support after an adverse event would be: (more than one answer possible)
1. Not applicable
2. Support is unnecessary
3. Professionally organised peer-support
4. Evaluation with the present team
5. Peer-support from direct colleagues (own department)
6. Peer-support from indirect colleagues (different department)
7. Mindfulness
8. Other …

17 It should be mandatory for the hospital to organise support after an adverse event:
1. Yes
2. No

18 You have learned to cope with adverse events through/during:
1. Med-school (without clerkships)
2. Clerkships
3. Internships
4. Residency
5. Attending physician
6. Other study
7. Psychological help
8. Specific course/training
9. Peer-review
10. Mindfulness
11. Never learnt
12. Other…

19 In the course of your career, you have become more defensive:
1. Strongly disagree
2. Disagree
3. Agree
4. Strongly agree

20 Have you changed your working-conditions (e.g. less shifts, more diagnostic tests) after experiencing a patient-related adverse event?
1. Yes
2. No

21 What did you change? (More than one answer possible)
1. Work less
2. Less evening/night-shifts
3. No evening/night-shifts anymore
4. Calling a colleague sooner
5. Quit my job
6. More diagnostic tests
7. Start treatment sooner
8. Other…

22 Sometimes people experience traumatic events, such as live-threatening situations as a cause of a natural disaster, high-impact trauma or fire; being attacked or raped; witness a murder, death or hear find out someone close to them experienced something terrible. As a physician, one can experience such events in patient-care: critical illness or death of a patients, severe injury, as well as violent behaviour from a patient or their family.
Have you ever, during your work AS A PHYSICIAN experienced such (adverse) events/incidents?
1. Yes
2. No

23 Can you briefly describe the adverse event(s)?
1. Yes, please describe it…
2. No

24 Did (some part of) this/these adverse event(s) take place more than 4 weeks ago?
1. Yes
2. No

Followed by the TSQ questionnaire.

35 It might be that you did not experience the reactions and emotions as described on the previous pages, but that you recognize them from a past period in your life. For example, having disturbing thoughts or dreams, reliving the event, getting upset. Do you recognize these reactions?
1. Yes
2. No

36 Which of these reactions do you recognize? (More than one answer possible)
1. Disturbing thoughts
2. Disturbing dreams
3. Reliving experiences
4. Getting upset
5. Physical responses
6. Insomnia
7. Irritability
8. Trouble focussing
9. Hyper arousal
10. Tensed

37 How long did these symptoms last?
1. < 4 weeks

2. > 4 weeks
3. > 6 months
4. > 1 year

38 Have you ever been diagnosed with PTSD (posttraumatic stress-disorder)?
1. Yes
2. No

Followed by the HADS questionnaire.

53 Have you ever seriously considered quitting your job as a paediatrician? (e.g. by looking for a different job, talking to human resources about ending your contract?
1. Yes, absolutely
2. Yes, sometimes
3. No, never

54 What was the reason to consider quitting? (More than one answer possible)
1. Bad collaboration (working together) with a co-worker
2. Not challenging enough within the field
3. A lot of complications
4. Afraid to make mistakes
5. Too much responsibility
6. Traumatic experience on the work-floor
7. Complaint(s) from patients
8. One-sided
9. A lot of stress
10. A high workload
11. Disbalance between work and private life
12. Disagreements with work providers
13. Different interests
14. New challenge
15. Too much administration
16. Too much bureaucracy
17. Patient-violence
18. Too many rules
19. Insufficient guidance from supervisors
20. Problems with the partnership
21. Private reasons
22. Disutility
23. Other…

55 Did you ever faced a complaint at the disciplinary board?
1. Yes
2. No

Gynaecologists

1 Gender
1. Female
2. Male

2 Age
1. 25-34 years
2. 35-44 years

3. 45-54 years
4. 55-64 years
5. 65 years and older

3 Current position
1. Resident
2. Attending / fellow
3. Retired gynaecologist
4. Non-practicing/management

4 How many years have you been working as a gynaecologist? (Including working as a resident)

5 I considered quitting my job as a gynaecologist:
1. Strongly disagree
2. Disagree
3. Agree
4. Strongly agree

6 In case you considered quitting, what was the most important reason?
1. Most important reason: …
2. Never considered quitting

7 Did you ever face a complaint at the disciplinary board?
1. Yes
2. No

8 What do you consider to be an adverse event on the work-floor? (More than one answer possible)
1. Not applicable
2. Bad news conversations/interview
3. The critical moments when a patient's life is at risk
4. When a patient dies
5. When you miss a diagnosis
6. When you feel like you cannot help the patients
7. When you are in doubt whether you are making the right decision
8. Other …

9 After experiencing adverse events on the work-floor, I do the following: (More than one answer possible)
1. Not applicable
2. Seeking professional help
3. Going home as soon as possible
4. Drink more alcohol, use more drugs and smoke more cigarettes than I normally would do
5. Use new medication I barely or normally not use
6. Trying not to think about it
7. Find a distraction
8. Praying or other religious activities
9. Formal case review
10. Work out or doing my hobby
11. Talking to friends and/or family
12. Informally discussing the matter with peers/colleagues
13. I developed symptoms of a burn-out (e.g. emotional exhaustion, depersonalisation, lower self-esteem)
14. Call in sick
15. Quit my job
16. Other …

10 The current support organized by your institution after an adverse event is good:
1. Strongly disagree
2. Disagree
3. Agree
4. Strongly agree

11 There is plenty of room to informally discuss adverse events in the department/partnership:
1. Strongly disagree
2. Disagree
3. Agree
4. Strongly agree

12 You have learned to cope with adverse events through/during:
1. Clerkships
2. Residency
3. Specific course/training
4. Intervision
5. Other …
6. Never really learned it

13 After experiencing an adverse event I changed my working conditions (e.g. no more shifts, no more vaginal breech births, not performing any surgeries alone, coming to the hospital sooner instead of supervision over the telephone)
1. Strongly disagree
2. Disagree
3. Agree
4. Strongly agree

14 In the course of your career, you have become more defensive:
1. Strongly disagree
2. Disagree
3. Agree
4. Strongly agree

15 Support after an adverse event consisted of… (fill in the box)

16 There is a protocol at your institution regarding support after an adverse event:
1. No
2. I do not know
3. Yes, consisting of…

17 Your preferred support after an adverse event would be: (more than one answer possible)
1. Evaluation with direct colleagues
2. Intervision with indirect colleague’s (physicians with a different specialty)
3. Talking to a psychologist or coach
4. A buddy
5. Other …

18 When you lay awake at night, you are not made for this job:
1. Strongly disagree
2. Disagree
3. Agree
4. Strongly agree

19 There should be a culture change regarding support after an adverse event:

1. Strongly disagree
2. Disagree
3. Agree
4. Strongly agree

20 Sometimes people experience traumatic events, such as live-threatening situations as a cause of a natural disaster, high-impact trauma or fire; being attacked or raped; witness a murder, death or hear find out someone close to them experienced something terrible. As a physician, one can experience such events in patient-care: critical illness or death of a patients, severe injury, as well as violent behaviour from a patient or their family.
1) Have you ever, during your work AS A PHYSICIAN experienced such (adverse) events/incidents?
2) After at least one of those adverse events, did you experience intense fear, helplessness or abomination?
3) Did this/these event(s) happened at least 4 weeks ago?

1. I answered yes to all of these questions
2. No, I did not answer yes to all these questions

Followed by the TSQ

31 Can you briefly describe the adverse event(s)?

32 It might be that you did not experience the reactions and emotions as described on the previous pages, but that you recognize them from a past period in your life. For example, having disturbing thoughts or dreams, reliving the event, getting upset. Do you recognize these reactions?

1. Yes
2. No

Followed by the HADS.

Othopaedic surgeons

1 What is your gender?

1. Male
2. Female

2 What is your age?

1. 20-29 years
2. 30-39 years
3. 40-49 years
4. 50-59 years
5. 60-69 years
6. ≥ 70 years

3 Are you a member of the Dutch Orthopaedic Society (NOV)?

1. Yes
2. No

4 What is your current position?

1. Resident
2. Attending physician
3. Retired
4. Non-practicing/management

5 How many years have you been working in the orthopaedic field as a physician? (Including as intern)

1. ≤ 5 years
2. 6-10 years
3. 11-15 years
4. 16-20 years
5. 21-25 years
6. 26-30 years
7. > 30 years

6 What do you consider to be an adverse event on the work-floor? (More than one answer possible)

1. Not applicable
2. Bad news conversation/interview
3. (Critically) ill patients
4. When a patient dies
5. When you miss a diagnosis
6. When you have to refuse a patient
7. When you are in doubt whether you are making the right decision
8. When an inexperienced colleague is on call
9. When you decide on an abstaining course of treatment
10. Treatment of young patients
11. When you know a patient will remain severely handicapped
12. Other …

7 How do you cope with adverse events on the work-floor? (More than one answer possible)

1. Not applicable / never experienced
2. Seeking professional help
3. Use new medication
4. Drink (more) alcohol
5. Smoke (more) cigarettes
6. Use (more) drugs
7. Work out (more)
8. Going home as soon as possible
9. Call in sick
10. Hide your emotions
11. Find a distraction
12. Praying or other religious activities
13. Talking to friends and/or family
14. Informally discussing the matter with peers/colleagues
15. Quitting
16. Work less
17. Other …

8 The current support organized by your institution after an adverse event is good:

1. Strongly disagree
2. Disagree
3. Agree
4. Strongly agree

9 There is plenty of room to informally discuss adverse events in the department/partnership:

1. Strongly disagree
2. Disagree
3. Agree
4. Strongly agree

10 There is plenty of room to informally discuss adverse events in the hospital:
1. Strongly disagree
2. Disagree
3. Agree
4. Strongly agree

11 There should be a culture change regarding support after an adverse event:
1. Strongly disagree
2. Disagree
3. Agree
4. Strongly agree

12 There is a protocol at your institution regarding support after an adverse event:
1. Yes
2. Yes, but nobody uses it
3. No, but there is one currently being created
4. No
5. I do not know

13 Support after an adverse event consisted of: (more than one answer possible)
1. I have never experienced an adverse event
2. There was none
3. Professionally organised peer-support
4. Self-initiated peer-support with direct colleagues (own department)
5. Self-initiated peer-support with indirect colleagues (different department)
6. Conversation(s) with a psychologist or coach
7. Evaluation with the present team
8. Help from the medical officer after a sick-leave
9. Other …

14 Your preferred support after an adverse event would be: (more than one answer possible)
1. Not applicable
2. Support is unnecessary
3. Professionally organised peer-support
4. Evaluation with the present team
5. Peer-support from direct colleagues (own department)
6. Peer-support from indirect colleagues (different department)
7. Mindfulness
8. Other …

15 It should be mandatory for the hospital to organise support after an adverse event:
1. Yes
2. No

16 You have learned to cope with adverse events through/during:
1. Med-school (without clerkships)
2. Clerkships
3. Internships
4. Residency
5. Attending physician
6. Psychological help
7. Specific course/training
8. Peer-review
9. Mindfulness
10. Never learnt

17 In the course of your career, you have become more defensive:
1. Strongly disagree
2. Disagree
3. Agree
4. Strongly agree

18 Have you changed your working-conditions (e.g. less shifts, more diagnostic tests) after experiencing a patient-related adverse event?
1. Yes
2. No

19 What did you change? (More than one answer possible)
1. Work less
2. Less evening/night-shifts
3. No evening/night-shifts anymore
4. Calling a colleague sooner
5. Quit
6. Start treatment sooner
7. Other…

20 Have you ever been diagnosed with PTSD (posttraumatic stress-disorder)?
1. Yes
2. No

21 Sometimes people experience traumatic events, such as live-threatening situations as a cause of a natural disaster, high-impact trauma or fire; being attacked or raped; witness a murder, death or hear find out someone close to them experienced something terrible. As a physician, one can experience such events in patient-care: critical illness or death of a patients, severe injury, as well as violent behaviour from a patient or their family.
Have you ever, during your work AS A PHYSICIAN experienced such (adverse) events/incidents?
1. Yes
2. No

22 Did this/these take place less than 4 weeks ago?
1. Yes
2. No

23 Can you describe the event/incident?
1. Yes
2. No

Followed by the TSQ and HADS questionnaire.

51 Have you ever seriously considered quitting your job as an orthopaedic surgeon? (e.g. by looking for a different job, talking to human resources about ending your contract?
1. Yes
2. No

52 What was the reason to consider quitting? (More than one answer possible)
1. Bad collaboration (working together) with a co-worker
2. Not challenging enough within the field
3. A lot of complication
4. Afraid to make mistakes
5. Too much responsibility

6. Traumatic experience on the work-floor
7. Complaint(s) from patients
8. One-sided
9. A lot of stress
10. A high workload
11. Disbalance between work and private life
12. Disagreements with work providers
13. Different interests
14. New challenge
15. Too much administration
16. Too much bureaucracy
17. Patient-violence
18. Too many rules
19. Insufficient guidance from supervisors
20. Problems with the partnership
21. Private reasons
22. Disutility
23. Other…

53 Did you ever face a complaint at the disciplinary board?

1. Yes
2. No
