# Supplementary material for: Potential traumatic events in the workplace and depression, anxiety and post-traumatic stress: a cross-sectional study among Dutch gynaecologists, paediatricians and orthopaedic surgeons
Source: BMJ Open. 2020 Sep 2;10(9):e033816. doi: 10.1136/bmjopen-2019-033816 (PMC7470507; doi:10.1136/bmjopen-2019-033816)
Supplement: Supplementary data [file bmjopen-2019-033816supp002.pdf]

Supplementary file 2: logistic regression model and odds ratios 'work experience – HADS-D and HADS-A'

Model summary HADSA

Model	Deviance	AIC	BIC	df	X²	p	McFadden R²	Nagelkerke R²	Tjur R²	Cox & Snell R²
H_0	1092.619	1094.619	1099.84	1371						
H_1	1078.140	1092.140	1128.70	1365	14.479	0.025	0.013	0.019	0.014	0.010

Coefficients

					Wald Test			95% Confidence interval (odds ratio scale)	
Work experience	Estimate	Standard Error	Odds Ratio	z	Wald Statistic	df	p	Lower bound	Upper bound
(Intercept)	-1.508	0.198	0.221	-7.595	57.690	1	< .001	0.150	0.327
5-10 years	-0.179	0.263	0.836	-0.680	0.463	1	0.496	0.500	1.400
10-15 years	-0.361	0.281	0.697	-1.284	1.650	1	0.199	0.401	1.209
15-20 years	-0.164	0.279	0.848	-0.589	0.347	1	0.556	0.491	1.465
20-25 years	-0.347	0.299	0.707	-1.160	1.345	1	0.246	0.393	1.271
25-30 years	-0.461	0.315	0.631	-1.461	2.134	1	0.144	0.340	1.170
>30 years	-1.116	0.341	0.328	-3.276	10.735	1	0.001	0.168	0.639

Note. HADSA level 'Abnormal' coded as class 1.

Model summary HADSD

Model	Deviance	AIC	BIC	df	X²	p	McFadden R²	Nagelkerke R²	Tjur R²	Cox & Snell R²
H_0	653.648	655.648	660.872	1371						
H_1	635.472	649.472	686.040	1365	18.176	0.006	0.028	0.035	0.033	0.013

Coefficients

					Wald Test			95% Confidence interval (odds ratio scale)	
	Estimate	Standard Error	Odds Ratio	z	Wald Statistic	df	p	Lower bound	Upper bound
(Intercept)	-3.314	0.416	0.036	-7.974	63.591	1	< .001	0.016	0.082
5-10 years	0.302	0.510	1.352	0.592	0.350	1	0.554	0.498	3.675
10-15 years	0.849	0.486	2.337	1.746	3.048	1	0.081	0.901	6.064
15-20 years	1.023	0.484	2.781	2.115	4.472	1	0.034	1.078	7.176
20-25 years	0.979	0.496	2.661	1.974	3.897	1	0.048	1.007	7.033
25-30 years	1.081	0.497	2.946	2.176	4.736	1	0.030	1.113	7.797
>30 years	-0.385	0.615	0.681	-0.626	0.392	1	0.531	0.204	2.270

Note. HADSD level 'Abnormal' coded as class 1.
