# Supplementary material for: Potential traumatic events in the workplace and depression, anxiety and post-traumatic stress: a cross-sectional study among Dutch gynaecologists, paediatricians and orthopaedic surgeons
Source: BMJ Open. 2020 Sep 2;10(9):e033816. doi: 10.1136/bmjopen-2019-033816 (PMC7470507; doi:10.1136/bmjopen-2019-033816)
Supplement: Author's manuscript [file bmjopen-2019-033816.draft_revisions.pdf]

BMJ Open is committed to open peer review. As part of this commitment we make the peer review history of every article we publish publicly available.

When an article is published we post the peer reviewers' comments and the authors' responses online. We also post the versions of the paper that were used during peer review. These are the versions that the peer review comments apply to.

The versions of the paper that follow are the versions that were submitted during the peer review process. They are not the versions of record or the final published versions. They should not be cited or distributed as the published version of this manuscript.

BMJ Open is an open access journal and the full, final, typeset and author-corrected version of record of the manuscript is available on our site with no access controls, subscription charges or pay-per-view fees (http://bmjopen.bmj.com).

If you have any questions on BMJ Open's open peer review process please email info.bmjopen@bmj.com

Potential traumatic events in the workplace and depression, anxiety and posttraumatic stress: a cross-sectional study among Dutch gynaecologists, paediatricians and orthopaedic surgeons.

Journal:	*BMJ Open*
Manuscript ID	bmjopen-2019-033816
Article Type:	Original research
Date Submitted by the Author:	26-Aug-2019

Complete List of Authors:	Scheepstra, Karel; Amsterdam UMC - Locatie AMC, Psychiatry<br>Pauw, Hannah; Amsterdam UMC - Locatie AMC, Faculty of Medicine<br>van Steijn, Minouk; Onze Lieve Vrouw Hosp, Obstetrics and Gynaecology; Amsterdam UMC - Locatie AMC, Obstetrics and Gynaecology<br>Stramrood, Claire; Amsterdam UMC - Locatie AMC, Obstetrics and Gynaecology<br>Olff, Miranda; Amsterdam UMC - Locatie AMC, Psychiatry; Arq Psychotrauma Research<br>van Pampus, Maria G.; Onze Lieve Vrouw Hosp, Obstetrics and Gynaecology

Keywords:	OCCUPATIONAL & INDUSTRIAL MEDICINE, PSYCHIATRY, Depression & mood disorders < PSYCHIATRY, Anxiety disorders < PSYCHIATRY, Adverse events < THERAPEUTICS, Protocols & guidelines < HEALTH SERVICES ADMINISTRATION & MANAGEMENT

SCHOLARONE™
Manuscripts

I, the Submitting Author has the right to grant and does grant on behalf of all authors of the Work (as defined in the below author licence), an exclusive licence and/or a non-exclusive licence for contributions from authors who are: i) UK Crown employees; ii) where BMJ has agreed a CC-BY licence shall apply, and/or iii) in accordance with the terms applicable for US Federal Government officers or employees acting as part of their official duties; on a worldwide, perpetual, irrevocable, royalty-free basis to BMJ Publishing Group Ltd ("BMJ") its licensees and where the relevant Journal is co-owned by BMJ to the co-owners of the Journal, to publish the Work in this journal and any other BMJ products and to exploit all rights, as set out in our licence.

The Submitting Author accepts and understands that any supply made under these terms is made by BMJ to the Submitting Author unless you are acting as an employee on behalf of your employer or a postgraduate student of an affiliated institution which is paying any applicable article publishing charge ("APC") for Open Access articles. Where the Submitting Author wishes to make the Work available on an Open Access basis (and intends to pay the relevant APC), the terms of reuse of such Open Access shall be governed by a Creative Commons licence – details of these licences and which Creative Commons licence will apply to this Work are set out in our licence referred to above.

Other than as permitted in any relevant BMJ Author's Self Archiving Policies, I confirm this Work has not been accepted for publication elsewhere, is not being considered for publication elsewhere and does not duplicate material already published. I confirm all authors consent to publication of this Work and authorise the granting of this licence.

For peer review only

Potential traumatic events in the workplace and depression, anxiety and posttraumatic stress: a cross-sectional study among Dutch gynaecologists, paediatricians and orthopaedic surgeons.

For peer review only

Authors

Karel W. F. Scheepstra*, MD[1]
Hannah S. Pauw, BSc[2]
Minouk E. van Steijn, MD[3,5]
Claire A.I. Stramrood, MD PhD[3]
Miranda Olff, PhD[1,4]
Maria G. van Pampus, MD PhD[5]

1. Amsterdam UMC, University of Amsterdam, Department of Psychiatry, Meibergdreef 9, 1105 AZ, Amsterdam, the Netherlands.
2. Amsterdam UMC, University of Amsterdam, Faculty of Medicine, Meibergdreef 9, 1105 AZ, Amsterdam, the Netherlands.
3. Amsterdam UMC, University of Amsterdam, Department of Obstetrics and Gynaecology, Meibergdreef 9, 1105 AZ, Amsterdam, the Netherlands.
4. Arq Psychotrauma Expert Group, Nienoord 5, 1112 XE, Diemen, the Netherlands
5. OLVG, Department of Obstetrics and Gynaecology, Oosterpark 9, 1090 HM, Amsterdam, the Netherlands

***Corresponding author:**
K.W.F. Scheepstra
Department of Psychiatry
Meibergdreef 9
1105 AZ Amsterdam
The Netherlands
k.w.scheepstra@amsterdamumc.nl
+31 (0) 20 8913500

ABSTRACT

Objective: To measure the prevalence of work-related potential traumatic events, support protocols and mental health symptoms amongst Dutch gynaecologists, orthopaedic surgeons and paediatricians.

Design: Cross-sectional study.

Setting: Nationwide survey between 2014 and 2017.

Participants: An online questionnaire was sent to all Dutch gynaecologists, orthopaedic surgeons and paediatricians (4959 physicians). 1374 questionnaires were eligible for analysis, corresponding with a response rate of 27.7%.

Outcome measures: Primary outcome measures are the prevalence of work-related potential traumatic events (PTEs), depression, anxiety, psychological distress and traumatic stress, measured with validated screening instruments (Hospital Anxiety and Depression Scale, HADS; Trauma Screening Questionnaire, TSQ). Secondary outcomes are the association of mental health and defensive practice to traumatic events and support protocols.

Results: Of the respondents, 20.8% experienced a work-related potential traumatic event at least four weeks ago. Prevalence rates indicative of depression, anxiety or posttraumatic stress disorder were respectively 6.4%, 13.6% and 1.5%. Comparing female versus male attendings, depression (9.2% vs. 5.2%, p=0.019), anxiety (18.2% vs. 8.2%, p<0.001) and psychological distress (22.8% vs. 12.5%, p<0.001), were significantly more prevalent in women. The absence of a support protocol was significantly associated with more probable posttraumatic stress disorder (p=0.022).

Conclusion: Physicians are frequently exposed to potential traumatic events with high emotional impact over the course of their career. Lacking a support protocol after adverse events was associated with more posttraumatic stress. Adverse events were associated with more dissatisfaction with work and a more defensive practice. More awareness must be created for the mental health of physicians as well as for the implementation of a well-organised support system after potential traumatic events.

Word count: 256

Keywords: Occupational & Industrial Medicine, Psychiatry, Depression & mood disorders, Anxiety disorders, Adverse Events, Protocols & guidelines

ARTICLE SUMMARY

Strengths and limitations of this study

- This is one of the few studies done on PTSD in physicians, in particular with regard to mental health in relation to social and occupational functioning and clinical practice related consequences.
- This study incorporated the A and F criterion in our probable PTSD diagnosis, therefore the prevalence is more likely to reflect the real prevalence of PTSD.
- One limitation is that the population of this study is limited to three specialties and one country in Europe and thus it is difficult to translate these results to physicians worldwide.

For peer review only

For peer review only - http://bmjopen.bmj.com/site/about/guidelines.xhtml

INTRODUCTION

Physicians are frequently exposed to medical adverse events, such as life-threatening situations, illnesses, grief, death, and patient violence and aggression. Dealing with such events makes the medical profession one with occupational health hazards, comparable with fire fighters, rescue workers, military and police personnel.[1–3] The support for physicians is, however, underdeveloped in most areas of the medical profession compared to other professions.[4,5] It is generally assumed that physicians have adequate coping mechanisms to deal with such work stressors. Physician health is one of the competencies described in the CanMEDS framework.[6] This framework recognises that, to provide optimal patient care, physicians must take responsibility for their own well-being and that of their colleagues. Several hospitals have started peer-support programmes, as this was shown to help physicians dealing with adverse events.[7]

Depression, anxiety and posttraumatic stress disorder

Major depressive disorder (MDD) is a common affective disorder that is characterised by a depressed mood and/or loss of pleasure.[8] A review on the epidemiology of mental disorders in Europe describes a one-year prevalence of 6.9% in the general population[9]; the Nemesis-2 study showed a one-year prevalence of mood disorders of 3.0% among Dutch citizens with a high income.[10] A meta-analysis on depressive symptoms and MDD among resident physicians showed high prevalence rates (20.9% to 43.2%).[11] Fewer studies were done on depressive symptoms among medical specialists, showing prevalence rates from 10.3 to 28.0%.[12–15] A recent literature review showed that as many as 40 per 100,000 physicians die by suicide each year in the United States of America, more than double the rate of the general population.[16]

Anxiety disorders include many different disorders, ranging from a specific phobia to a generalised anxiety disorder. The one-year prevalence for anxiety disorders in the Netherlands is 6.0% among citizens with a high income and 10.1% among citizens with a low income.[10]

PTSD is a highly debilitating disorder that can develop after exposure to a psychotraumatic event.[17] Symptoms of PTSD include intrusions, avoidance of stimuli related to the trauma, alterations in

For peer review only - http://bmjopen.bmj.com/site/about/guidelines.xhtml

cognition or mood, and hyperarousal. The point prevalence of posttraumatic stress symptoms among physicians ranges between 3.8% and 15.0.[12,18–22]

This study investigates the prevalence of potential traumatic events (PTEs) and support protocols in relation to symptoms of depression, anxiety and posttraumatic stress disorder (PTSD) among Dutch physicians. This study also investigates the differences between medical specialties, gender and position.

METHODS

Population and procedure

Each physician received one invitation for an online questionnaire (supplementary file 1) and two reminders over an 8-week period. The questionnaire was sent through Thesistools® or Surveymonkey®, creating an anonymous (non-traceable) e-mail link. The questionnaire was sent to 1589 gynaecologists, 2160 paediatricians and 1210 orthopaedic surgeons, with a total of 4959 physicians, including residents. The invitation to participate in the questionnaire for the gynaecologists was sent in 2014, orthopaedic surgeons in 2016 and paediatricians in 2017.

The membership databases of the Dutch Society of Obstetrics & Gynaecology (NVOG), Dutch Society of Orthopaedic Surgeons (NOV) and the Dutch Society of Paediatricians (NvK) were used. Registration as a medical specialist or resident in one of these societies is obligatory and so all Dutch medical specialists in these fields were contacted. These databases contained residents, attending, retired and non-practicing physicians.

This study was exempted from ethical approval by the Medical Research Ethics Committees United (MEC-U), and registered under numbers W17.169 and W18.096.

For peer review only - http://bmjopen.bmj.com/site/about/guidelines.xhtml

Patient and public involvement

Engagement with the described medical societies was vital to ensure participation and a high response rate. The qualitative questions were piloted among several medical specialists of each specific medical specialty.

Measurements

The instruments included the Hospital Anxiety and Depression Scale (HADS) and the Trauma Screening Questionnaire (TSQ) as validated screening instruments. The HADS is a 14-item screening instrument for depression and anxiety, where the subscales contain seven questions each. The cut-off value of the Dutch version of the depression (HADS-D) and anxiety subscale (HADS-A) is equal to or higher than eight. The total HADS cut-off value for general distress is equal or higher than 12, corresponding to clinically relevant psychological distress; this is a rather non-specific but sensitive measurement to screen for mental disorders.[23] The sensitivity and specificity for both HADS-A and HADS-D are approximately 0.80, with Cronbach's alphas for HADS-A from 0.68 to 0.93 (mean 0.83) and for HADS-D from 0.67 to 0.90, in different populations. [24]

The TSQ is a ten-item screening instrument for a probable diagnosis of PTSD according to the DSM-IV, with a sensitivity of 0.94 and specificity of 0.56 when using a cut-off of six and higher. The Dutch psychometric properties are validated with Cronbach's alphas from 0.71 to 0.91.[25] Only respondents who answered 'yes' to experiencing a work-related traumatic event at least four weeks ago were asked to fill out the TSQ.

Additionally, the survey contained questions about demographics, personal experiences, coping and support strategies after adverse events in the workplace. Regarding the latter, multiple-choice options were given as well as an open field where respondents were able to add their individual answers or comment on their experience. These qualitative questions were piloted among several medical specialists of each specific medical specialty, to increase face value of the questions (supplementary file 2).

For peer review only - http://bmjopen.bmj.com/site/about/guidelines.xhtml

Statistical analysis

Statistical analysis was performed using IBM Statistical Package for the Social Sciences (SPSS) version 22 and JASP version 0.8.5.1. Demographic data and multiple-choice questions were analysed using descriptive statistics and exported as frequency tables and bar charts. Differences in outcomes (in various groups) for categorical variables were tested using either a Chi-squared test or a Fisher's exact test where applicable. A two-sided p-value equal to or less than 0.05 was considered statistically significant. Logistic regression with odds-ratio was done and adjusted for potential confounders, a p-value equal to or less than 0.05 was considered statistically significant.

For peer review only - http://bmjopen.bmj.com/site/about/guidelines.xhtml

RESULTS

A total of 1374 questionnaires were collected with a response rate of 27.7%. Table 1 shows the respondents characteristics by specialty.

Table 1: Respondent characteristics

	Total (n=4959)	Gynaecologists (n=1589)	Orthopaedic surgeons (n=1210)	Paediatricians (n=2160)
**Response rate**	27.7	48.9	21.3	29.8
**Gender**				
Male	44.9	34.7	85.6	32.7
Female	55.1	65.3	14.4	67.3
**Position**				
Resident	23.0	26.9	20.9	18.0
Attending physician	67.2	64.7	62.3	74.9
Retired	7.1	5.4	13.4	5.6
Non-practicing	2.6	3.0	3.4	1.5
**Years in practice**				
≤ 5	12.4	14.3	12.0	9.8
6-10	18.6	19.0	19.5	17.3
11-15	15.8	17.7	14.0	13.9
16-20	14.3	11.6	13.4	19.3
21-25	12.4	12.8	9.2	13.9
26-30	11.3	9.5	14.4	12.0
>30	15.1	14.7 *	17.5	13.9
**Complaints disciplinary board**	19.9	21.0	29.8	11.2
**Support protocol**				
Yes	19.6	15.8	18.8	26.3
No	42.4	52.7	30.1	34.1
I do not know	38.0	31.4	51.0	39.5

All values shown as %
*=2 missing

The majority of the orthopaedic surgeons were male (85.6%) while in the other specialties the majority were female. In all specialties the majority of the respondents were attending physicians. The gender and position distributions were compared with the membership databases of the societies, and for none of the specialties the distribution in the sample was significantly different from the population. The orthopaedic surgeons reported to have had the most complaints at a disciplinary board. One hundred eight (26.3%) paediatricians reported to have a support protocol, whereas only 18.8% of the orthopaedic surgeons and 15.8% of the gynaecologists report to have a protocol after adverse events.

Outcomes of the HADS by specialty are shown in table 1.

For peer review only - http://bmjopen.bmj.com/site/about/guidelines.xhtml

Table 2: Mental health – Outcomes HADS + TSQ by specialty

	Total (n=1374)	Gynaecologists (n=672)	Orthopaedic Surgeons (n=292)	Paediatricians (n=410)
**Depression**	6.4	6.5	4.8	7.3
**Anxiety**	13.6	15.8	8.2	14.1
**Psychological distress**	17.1	18.2	12.0	19.3
**PTSD**				
PTE ≥ 4 weeks ago	20.8	12.8	19.5	34.9
PTS symptoms earlier	13.4	8.2	8.6	25.4
TSQ score above cut-off	1.5	1.5	0.3	2.2

All values shown in %
Abbreviations: Potential Traumatic Event (PTE); Trauma Screening Questionnaire (TSQ); Hospital Anxiety and Depression Scale (HADS); Posttraumatic stress (PTS); Posttraumatic stress disorder (PTSD).

For peer review only

Table 3: Mental health - Outcomes HADS by position (resident vs. attending) and gender

	Total (n=1240)	Depression	Anxiety	Psychological distress
**Total**		6.7	15.2	19.0
Male	40.8	5.5	10.1	14.2
Female	59.2	8.2	*** 18.7	*** 22.3
**Residents**	25.4	4.7	17.1	18.4
Male	25.6	3.7	13.6	14.8
Female	74.4	5.1	18.3	19.6
**Attending physicians**	74.6	7.4	13.6	18.1
Male	45.9	5.2	8.2	12.5
Female	54.1	* 9.2	*** 18.2	*** 22.8

All values shown as %
*=p<0.05, **p<0.01, ***p<0.001 (χ^2)
Abbreviations: Hospital Anxiety and Depression Scale (HADS)

Eighty eight (6.4%) respondents scored above the cut-off value for depression and one hundred and eighty eight (13.6%) respondents scored above the cut-off value for anxiety. Two hundred and thirty six (17.1%) scored above the cut-off value of the total HADS, corresponding to clinically relevant psychological distress. When comparing specialties, there is no significant difference in depression. However, looking at the prevalence of anxiety and psychological distress, the orthopaedic surgeons scored significantly lower compared to both gynaecologists and paediatricians.

As seen in table 3, no significant difference was found between male and female residents. When comparing all male versus female physicians, the prevalence rates of anxiety (10.1% vs. 18.7%, p<0.001) and the total HADS indicating psychological distress (14.2% vs. 22.3%, p<0.001) were

For peer review only - http://bmjopen.bmj.com/site/about/guidelines.xhtml

significantly higher among female physicians. When comparing male and female attendings, the rates of depression (5.2% vs. 9.2%, p=0.019), anxiety (8.2% vs. 18.2%, p<0.001) and the total HADS (12.5·0% vs. 22.8%, p<0.001) were significantly higher among female attendings. The highest rate of respondents with a score above the cut-off value for anxiety were found among physicians with 6-10 years in practice (supplementary file 3). There were no differences in primary outcomes when comparing residents with attendings. There was a significant increase in the prevalence of depression in the groups with more years in practice (odds ratio 2.95, p=0.006, supplementary file 3), but not with increased age. We found a significant decrease in the prevalence of anxiety with more years in practice (odds ratio 0.33, p=0.025, supplementary file 3).

Posttraumatic stress disorder

Two hundred eighty six respondents (20.8%) reported having experienced at least one potential traumatic event (PTE) during their work, at least four weeks ago, thereby meeting DSM-IV criterion A and F. Of these respondents, 183 reported recognising posttraumatic stress (PTS) symptoms after this event (13.4% of the total group, 64.3% of the potentially traumatised group) and 20 screened positive for probable PTSD (1.5% of the total group, 7.0% of the potentially traumatised group, table 2). Of the paediatricians 34.9% reported having experienced a PTE at least four weeks ago. This is significantly higher than the percentages of the gynaecologists (12.8%, p<0.0001) and orthopaedic surgeons (19.5%, p<0.0001). When comparing the prevalence of probable PTSD, the paediatricians had a significant higher prevalence compared to the orthopaedic surgeons (2.2% vs. 0.3%, p=0.041). Both the paediatricians and gynaecologists report to recognise more PTS symptoms. When comparing gender and position (resident vs. attending, table 4), residents are less likely to have experienced a PTE (16.8% vs. 22.6%, p=0.028) and thus also report significantly less PTS symptoms earlier (7.9% vs. 15.6%, p<0.001).

For peer review only - http://bmjopen.bmj.com/site/about/guidelines.xhtml

Table 4: Mental health - Outcomes TSQ by position (resident vs. attending) and gender

	Total (n=1240)		PTE		PTS symptoms earlier	PTSD
**Total**			21.1		13.6	1.5
Male	40.8		21.3	*	11.3	1.2
Female	59.2		21.0		15.3	1.6
**Residents**	25.4		16.8		7.9	0.9
Male	25.6		21.0		4.9	0.0
Female	74.4	*	15.3	***	8.9	1.3
**Attending**	74.6		22.6		15.6	1.6
Male	45.9		21.4		12.5	1.4
Female	54.1		23.6	*	18.2	1.8

All values shown as %

*=p<0.05, **p<0.01, ***p<0.001 (χ^2)

Abbreviations: Potential Traumatic Event (PTE); Trauma Screening Questionnaire (TSQ); Hospital Anxiety and Depression Scale (HADS); Posttraumatic stress (PTS); Posttraumatic stress disorder (PTSD).

For peer review only

The highest rate of probable PTSD was found among attendings with 15 respondents (1.6%) compared to 3 of the residents (0.9%). When comparing gender, male and female physicians report the same number of PTEs, but female physicians are more likely to report earlier PTS-symptoms (15.3% vs. 11.3%, p=0.044). Those that witnessed a PTE, reported significantly more defensive work changes after an incident (28.0% vs 20.5%, p=0.007), but they did not consider to quit as a physician more often (37.5% vs 35.0%, p=0.45). However, those that recognised PTS-symptoms after an incident did consider quitting work more often (42.6% vs. 29.2%, p=0.029). Those with current probable PTSD became more defensive (45.0% vs. 26.7%, trend, p=0.079) and significantly considered quitting medical work more often (60.0% vs. 35.8%, p=0.032).

suppl

DISCUSSION

Depression and anxiety

This study examined the point prevalence rates of probable depression, anxiety, psychological distress, work-related PTE and PTSD among gynaecologists, orthopaedic surgeons and paediatricians. It is part of the Work-related Adverse and Traumatic Events Research (WATER) and this paper combines the results of several previous studies [26,27], to show differences between support protocols, specialties, gender and position. Our point prevalence rates of depression (6.4%) and anxiety (13.6%), are high

For peer review only - http://bmjopen.bmj.com/site/about/guidelines.xhtml

compared to 12-month prevalence rates of mood disorders (3.0%) and anxiety (6.0%) in high income populations in the Netherlands, and compared to European 12-month prevalence rate of anxiety (6.4%).[9,10] The point prevalence rates in this study are comparable to American 12-month general prevalence rates of depression (6.7%) and anxiety (18.1%).[28,29] This indicates that physicians are more depressed and anxious compared to the general population and the population with high income in the Netherlands. This high prevalence is in line with current literature, where even higher prevalence rates for depression and anxiety were found among medical students, residents and specialists.[11,12,30] We found a significant increase in the prevalence of depression corresponding with more years in practice. Furthermore, this study showed a significantly higher prevalence of depression and anxiety among female compared to male attendings. This finding correlates with previous study results,[31] where prevalence rates for depression and anxiety were higher among British female doctors compared to males. These findings are also in line with epidemiologic data on the gender distribution of mood and anxiety disorders in the general population.[9,10] This finding is relevant, because of the feminization of medical specialties globally, and an increase in female physicians of 1.0 to 2.0 % each year in the last ten years in the Netherlands.[32] One of the relevant findings of this study is the high prevalence of psychological distress (17.6%). Scoring above this cut-off is not specific for one diagnosis, but does indicate a high level of distress and a risk for potential unfit physicians.

Posttraumatic stress disorder

In this sample, 285 respondents (20.8%) reported to have experienced a potential traumatic event (PTE) that took place longer than four weeks ago. This is within the range of the estimated prevalence of second victims of 10.4% and 43.3%, reported by Seys et al.[33] Our prevalence for work-related PTSD (1.5%) is slightly higher, compared to the general Dutch prevalence of PTSD (1.2%).[34] The Dutch prevalence of only work-related PTSD is not known, however assumed to be lower. Of the potential traumatised respondents, 7.0% screened positive for PTSD, which is comparable to other

For peer review only - http://bmjopen.bmj.com/site/about/guidelines.xhtml

occupations with mental health hazards, such as police officers after critical police incidents (7.0%).[35]

One of the key findings is that after experiencing a PTE, the absence of a support protocol is significantly associated with more probable PTSD (p=0.022). This once again shows the importance of support protocols and implicates that a well perceived support system may prevent posttraumatic stress complaints.

Of the residents who filled out the TSQ, three screened positive for PTSD (all female respondents). These findings are in line with the known gender distribution, as women are more likely to develop PTSD than men.[36] Other studies found higher prevalence rates of PTSD (i.e. Joseph[18] = 15%, Mills[21] = 11.9%, Ruitenburg[12] =15%, and Shi[19] = 3.8%), but they used different (non-) validated screening instruments and methods. None of these screening instruments assessed when the PTE happened (for a diagnosis of PTSD this needs to be at least four weeks ago), meaning that their respondents may not meet criterion A of PTSD and could also be suffering from acute stress symptoms. Furthermore, these studies did not assess whether the potential event was work-related, so these numbers show general PTSD prevalence rates which are usually higher.

Studies have shown that dysfunctional coping can predict complicated grief and PTSD severity.[37,38] Thus, it is important to educate physicians about coping mechanisms and stress management techniques to prevent adverse mental health conditions.[39] With social support being an important protective factor for PTSD[40], the role of educating health care professionals to address this problem and support their colleagues in peer-support groups is crucial.[7]

Job related consequences of depression, anxiety, adverse events and PTSD

This study shows that physicians consider quitting their job more often, after having experienced PTS-symptoms in the past or currently suffering from probable PTSD. Experiencing a PTE is also associated with more defensive medical practice. When looking at the prevalence of complaints at a disciplinary board, the orthopaedic surgeons reported the most complaints (table 1). This may be due

For peer review only - http://bmjopen.bmj.com/site/about/guidelines.xhtml

to the higher amount of elective work they do, which usually leads to more dissatisfied patients, compared to emergency work. More complaints was not associated with considering quitting work or increased rates of depression, anxiety or psychological distress.

Strengths

Little research has been done on PTSD in physicians, in particular with regard to mental health in relation to social and occupational functioning and clinical practice related consequences. We incorporated the A and F criterion in our probable PTSD diagnosis, therefore our prevalence is more likely to reflect the real prevalence of PTSD. Also, this study included a large number (n=1374) of physicians, which made it possible to analyse gender, position and age differences. The overall response rate among gynaecologists was high (42.9%), followed by the orthopaedic surgeons (29%) and the paediatricians (18.9%). The overall response rate was 27.7%, which is slightly higher than the average response rate for email surveys (25.0%)[41] and comparable to similar surveys in this field [42].

Limitations

There are several limitations to this study. The population of this study is limited to three specialties and one country in Europe and thus it is difficult to translate these results to physicians worldwide. However, it is known that working conditions and related stress levels are comparable with the rest of Europe and North-America. Also, avoidance is one of the key symptoms of PTSD and can therefore lead to non-response and underestimation of the PTSD-prevalence. On the other hand, this self-selection bias may also lead to overrepresentation of physicians with strong opinions. Due to the low numbers of PTSD, it was not possible to correlate the type of adverse event with more PTSD. Furthermore, the HADS and TSQ are screening instruments, and therefore only give an estimate of the prevalence rates of probable disorders. Only the prevalence of PTSD is work-related, the numbers for depression and anxiety are general prevalence rates.

For peer review only - http://bmjopen.bmj.com/site/about/guidelines.xhtml

CONCLUSION

Physicians are often exposed to PTEs over the course of their career with potentially high emotional impact. The prevalence rates for probable depression (6.4%) and anxiety (13.6%) were higher among physicians, compared to the general population with high income in the Netherlands. The point prevalence of clinical psychological distress was 19.0% and of probable PTSD was 1.5%. Female physicians are significantly more prone to develop depressive- and anxiety disorders than their male colleagues. PTEs were associated with more defensive practice and dissatisfaction about work. And importantly, the absence of a support protocol was associated with more probable PTSD. This once again shows the importance of support protocols and implicates that a well perceived support system may prevent posttraumatic stress complaints and unfit physicians.

For peer review only

For peer review only - http://bmjopen.bmj.com/site/about/guidelines.xhtml

ACKNOWLEDGEMENTS

We would like to thank the Dutch Society of Obstetrics & Gynaecology (NVOG), Dutch Society of Orthopaedic Surgeons (NOV), the Dutch Society of Paediatricians (NvK), and their members, for their cooperation in this study.

For peer review only

For peer review only - http://bmjopen.bmj.com/site/about/guidelines.xhtml

COMPETING INTERESTS

None of the authors declare a conflict of interest.

For peer review only

FUNDING

This research received no specific grant from any funding agency in the public, commercial or not-for-profit sectors.

For peer review only

CONTRIBUTORSHIP

KS, MvS, CS, MvP were involved in the design of this study. KS and HP analysed the data and made the first draft. All authors were involved in finalising the manuscript.

For peer review only

For peer review only - http://bmjopen.bmj.com/site/about/guidelines.xhtml

DATA AVAILABILITY

The data can be made available by the corresponding author upon reasonable request and for a well defined purpose. The request will be discussed in our research group. The data will be de-identified participant data. A statistical analysis plan is available.

For peer review only

For peer review only - http://bmjopen.bmj.com/site/about/guidelines.xhtml

REFERENCES

1 Yehuda R, Vermetten E, McFarlane AC, *et al.* PTSD in the military: Special considerations for understanding prevalence, pathophysiology and treatment following deployment. *Eur J Psychotraumatol* Published Online First: 2014. doi:10.3402/ejpt.v5.25322

2 van der Meer CAI, Bakker A, Schrieken BAL, *et al.* Screening for trauma-related symptoms via a smartphone app: The validity of Smart Assessment on your Mobile in referred police officers. *Int J Methods Psychiatr Res* Published Online First: 2017. doi:10.1002/mpr.1579

3 Mellotte H, Murphy D, Rafferty L, *et al.* Pathways into mental health care for UK veterans: a qualitative study. *Eur J Psychotraumatol* Published Online First: 2017. doi:10.1080/20008198.2017.1389207

4 Edrees H, Federico F. Supporting clinicians after medical error. *BMJ* 2015. doi:10.1136/bmj.h1982

5 Devi S. Doctors in distress. *Lancet* Published Online First: 2011. doi:10.1016/S0140-6736(11)60145-1

6 Frank JR, Snell L, Sherbino J E. CanMEDs 2015 Physician Competency Framework. CanMEDS 2015 Physician Competency Fram. Ottawa R. Coll. Physicians Surg. Canada. 2015;:1–30.

7 Hu YY, Fix ML, Hevelone ND, *et al.* Physicians' needs in coping with emotional stressors: The case for peer support. *Arch Surg* Published Online First: 2012. doi:10.1001/archsurg.2011.312

8 American Psychiatric Association. Diagnostic and Statistical Manual of Mental Disorders: DSM-5. *Am Psychiatr Assoc* 2013;:991. doi:10.1176/appi.books.9780890425596.744053

9 Alonso J, Angermeyer MC, Bernert S, *et al.* Prevalence of mental disorders in Europe: results from the European Study of the Epidemiology of Mental Disorders (ESEMeD) project. *Acta Psychiatr Scand Suppl* 2004;**109**:21–7. doi:10.1111/j.1600-0047.2004.00327.x

10 De Graaf R, Ten Have M, Van Gool C, *et al.* Prevalence of mental disorders and trends from 1996 to 2009. Results from the Netherlands Mental Health Survey and Incidence Study-2. Soc. Psychiatry Psychiatr. Epidemiol. 2012;**47**:203–13. doi:10.1007/s00127-010-0334-8

11 Mata DA, Ramos MA, Bansal N, *et al.* Prevalence of Depression and Depressive Symptoms Among Resident Physicians: A Systematic Review and Meta-analysis. *JAMA* 2015;**314**:2373–83. doi:10.1001/jama.2015.15845

12 Ruitenburg MM, Frings-Dresen MH, Sluiter JK. The prevalence of common mental disorders among hospital physicians and their association with self-reported work ability: a cross-sectional study. *BMC Health Serv Res* 2012;**12**:292. doi:10.1186/1472-6963-12-292

13 Wurm W, Vogel K, Holl A, *et al.* Depression-burnout overlap in physicians. *PLoS One* Published Online First: 2016. doi:10.1371/journal.pone.0149913

14 Hsu K, Marshall V. Prevalence of depression and distress in a large sample of Canadian residents, interns, and fellows. *Am J Psychiatry* Published Online First: 1987. doi:10.1176/ajp.144.12.1561

For peer review only - http://bmjopen.bmj.com/site/about/guidelines.xhtml

15 Tomioka K, Morita N, Saeki K, *et al.* Working hours, occupational stress and depression among physicians. *Occup Med (Chic Ill)* Published Online First: 2011. doi:10.1093/occmed/kqr004

16 O T, A A, D T, *et al.* Physician Suicide: A Silent Epidemic. In: *In: APA annual meeting, Poster no 227, New York City*. 2018. 123–4.

17 Kessler RC, Aguilar-Gaxiola S, Alonso J, *et al.* Trauma and PTSD in the WHO World Mental Health Surveys. Eur. J. Psychotraumatol. 2017. doi:10.1080/20008198.2017.1353383

18 Joseph B, Pandit V, Hadeed G, *et al.* Unveiling posttraumatic stress disorder in trauma surgeons. *J Trauma Acute Care Surg* 2014;**77**:148–54. doi:10.1097/TA.0000000000000271

19 Shi L, Wang L, Jia X, *et al.* Prevalence and correlates of symptoms of post-traumatic stress disorder among Chinese healthcare workers exposed to physical violence: a cross-sectional study. *BMJ Open* 2017;**7**:e016810. doi:10.1136/bmjopen-2017-016810

20 Chan AOM, Huak CY. Influence of work environment on emotional health in a health care setting. *Occup Med (Lond)* 2004;**54**:207–12. doi:10.1093/occmed/kqh062

21 Mills LD, Mills TJ. Symptoms of post-traumatic stress disorder among emergency medicine residents. J. Emerg. Med. 2005. doi:10.1016/j.jemermed.2004.05.009

22 Wahlberg, Andreen Sachs M, Johannesson K, *et al.* Post-traumatic stress symptoms in Swedish obstetricians and midwives after severe obstetric events: a cross-sectional retrospective survey. *BJOG An Int J Obstet Gynaecol* Published Online First: 2017. doi:10.1111/1471-0528.14259

23 Spinhoven P, Ormel J, Sloekers PP, *et al.* A validation study of the Hospital Anxiety and Depression Scale (HADS) in different groups of Dutch subjects. *Psychol Med* 1997;**27**:363–70. doi:10.1017/S0033291796004382

24 Bjelland I, Dahl AA, Haug TT, *et al.* The validity of the Hospital Anxiety and Depression Scale. An updated literature review. *J Psychosom Res* 2002.

25 Dekkers AMM, Olff M, Maring GWB. Identifying persons at risk for PTSD after trauma with TSQ in the Netherlands. *Community Ment Health J* 2010;**46**:20–5. doi:10.1007/s10597-009-9195-6

26 Baas MAM, Scheepstra KWF, Stramrood CAI, *et al.* Work-related adverse events leaving their mark: A cross-sectional study among Dutch gynecologists. *BMC Psychiatry* 2018;**18**. doi:10.1186/s12888-018-1659-1

27 van Steijn ME, Scheepstra KWF, Yasar G, *et al.* Occupational well-being in pediatricians—a survey about work-related posttraumatic stress, depression, and anxiety. *Eur J Pediatr* Published Online First: 2019. doi:10.1007/s00431-019-03334-7

28 Kessler RC, Chiu WT, Demler O, *et al.* Prevalence, severity, and comorbidity of 12-month DSM-IV disorders in the National Comorbidity Survey Replication. *Arch Gen Psychiatry* 2005;**62**:617–27. doi:10.1001/archpsyc.62.6.617

29 Substance Abuse and Mental Health Services Administration, Center for Behavioural Health Statistics and Quality. Results from the 2015 National Survey on Drug Use and Health: Detailed Tables,. 2015.

30 Hope V, Henderson M. Medical student depression, anxiety and distress outside North America: a systematic review. *Med Educ* 2014;**48**:963–79. doi:10.1111/medu.12512

31 Wall TD, Bolden RI, Borrill CS, *et al.* Minor psychiatric disorder in NHS trust staff:

For peer review only - http://bmjopen.bmj.com/site/about/guidelines.xhtml

Occupational and gender differences. *Br J Psychiatry* 1997;**171**:519–23. doi:10.1192/bjp.171.6.519

32 Capaciteitsorgaan. Capaciteitsplan 2016 (in Dutch). Utrecht: 2016. www.capaciteitsorgaan.nl accessed 2-7-2018

33 Seys D, Wu AW, Gerven E Van, *et al.* Health Care Professionals as Second Victims after Adverse Events. *Eval Health Prof* 2013;**36**:135–62. doi:10.1177/0163278712458918

34 De Vries GJ, Olff M. The lifetime prevalence of traumatic events and posttraumatic stress disorder in the Netherlands. *J Trauma Stress* Published Online First: 2009. doi:10.1002/jts.20429

35 Carlier I V, Lamberts RD, Gersons BP. Risk factors for posttraumatic stress symptomatology in police officers: a prospective analysis. *J Nerv Ment Dis* Published Online First: 1997. doi:10.1097/00005053-199708000-00004

36 Olff M, Langeland W, Draijer N, *et al.* Gender differences in posttraumatic stress disorder. [Review] [287 refs]. *Psychol Bull* Published Online First: 2007. doi:10.1037/0033-2909.133.2.183

37 Schnider KR, Elhai JD, Gray MJ. Coping style use predicts posttraumatic stress and complicated grief symptom severity among college students reporting a traumatic loss. *J Couns Psychol* 2007;**54**:344–50. doi:10.1037/0022-0167.54.3.344

38 Kerai SM, Khan UR, Islam M, *et al.* Post-traumatic stress disorder and its predictors in emergency medical service personnel: a cross-sectional study from Karachi, Pakistan. *BMC Emerg Med* 2017;**17**:26. doi:10.1016/j.brat.2003.07.010

39 Brosschot JF. Ever at the ready for events that never happen. *Eur J Psychotraumatol* Published Online First: 2017. doi:10.1080/20008198.2017.1309934

40 Olff M. Bonding after trauma: On the role of social support and the oxytocin system in traumatic stress. *Eur J Psychotraumatol* Published Online First: 2012. doi:10.3402/ejpt.v3i0.18597

41 Fluid Surveys University. Response Rate Statistics for Online Surveys - What Numbers Should You be Aiming For? 2014.

42 Shanafelt TD, Boone S, Tan L, *et al.* Burnout and satisfaction with work-life balance among US physicians relative to the general US population. *Arch Intern Med* Published Online First: 2012. doi:10.1001/archinternmed.2012.3199

For peer review only - http://bmjopen.bmj.com/site/about/guidelines.xhtml

Supplementary files

1. Questionnaire
2. Supplementary table: logistic regression work experience vs. HADS-D and HADS-A

For peer review only

For peer review only - http://bmjopen.bmj.com/site/about/guidelines.xhtml

Supplementary file 2: Questionnaires

Paediatricians

1 What is your gender?
1. Male
2. Female

2 What is your age?
1. 20-29 years
2. 30-39 years
3. 40-49 years
4. 50-59 years
5. 60-69 years
6. ≥ 70 years

3 Are you a member of the Dutch Society of Paediatricians (NvK)?
1. Yes
2. No

4 What is your current position?
1. Resident
2. Attending physician
3. Retired
4. Non-practicing/management

5 How many years have you been working in the paediatrics field as a physician? (Including as intern)
1. ≤ 5 years
2. 6-10 years
3. 11-15 years
4. 16-20 years
5. 21-25 years
6. 26-30 years
7. > 30 years

6 At the moment, are you working in a general hospital or in an academic referral centre?
1. General hospital
2. Academic referral centre
3. Both
4. I do not work anymore, but I used to work in a general hospital
5. I do not work anymore, but I used to work in an academic referral centre

7 At which department do you work the most? (If you are not practicing anymore, fill in where you used to work)
1. Neonatology
2. Emergency room
3. General paediatrician
4. NICU
5. Child intensive care
6. Policlinic
7. Children's oncology
8. Other…

8 What do you consider to be an adverse event on the work-floor? (More than one answer possible)
1. Not applicable

For peer review only

2. Bad news conversation/interview
3. (Critically) ill neonate
4. (Critically) ill children
5. When a patient dies
6. When you miss a diagnosis
7. When you have to refuse a patient
8. When you are in doubt whether you are making the right decision
9. When an inexperienced colleague is on call
10. When you decide on an abstaining course of treatment
11. Treatment of neonates
12. When you know a patient will remain severely handicapped
13. Handling the emotions of the parents
14. (Suspicion of) child abuse
15. Other …

9 How do you cope with adverse events on the work-floor? (More than one answer possible)

1. Not applicable / never experienced
2. Seeking professional help
3. Use new medication
4. Drink (more) alcohol
5. Smoke (more) cigarettes
6. Use (more) drugs
7. Work out (more)
8. Going home as soon as possible
9. Call in sick
10. Hide your emotions
11. Find a distraction
12. Praying or other religious activities
13. Talking to friends and/or family
14. Informally discussing the matter with peers/colleagues
15. Quitting
16. Work less
17. Not working any evening/night shifts
18. Other …

10 The current support organized by your institution after an adverse event is good:

1. Strongly disagree
2. Disagree
3. Agree
4. Strongly agree

11 There is plenty of room to informally discuss adverse events in the department/partnership:

1. Strongly disagree
2. Disagree
3. Agree
4. Strongly agree

12 There is plenty of room to informally discuss adverse events in the hospital:

1. Strongly disagree
2. Disagree
3. Agree
4. Strongly agree

13 There should be a culture change regarding support after an adverse event:

1. Strongly disagree

For peer review only

2. Disagree
3. Agree
4. Strongly agree

14 There is a protocol at your institution regarding support after an adverse event:
1. Yes
2. Yes, but nobody uses it
3. No, but there is one currently being created
4. No
5. I do not know

15 Support after an adverse event consisted of: (more than one answer possible)
1. I have never experienced an adverse event
2. There was none
3. Professionally organised peer-support
4. Self-initiated peer-support with direct colleagues (own department)
5. Self-initiated peer-support with indirect colleagues (different department)
6. Conversation(s) with a psychologist or coach
7. Evaluation with the present team
8. Help from the medical officer after a sick-leave
9. Other …

16 Your preferred support after an adverse event would be: (more than one answer possible)
1. Not applicable
2. Support is unnecessary
3. Professionally organised peer-support
4. Evaluation with the present team
5. Peer-support from direct colleagues (own department)
6. Peer-support from indirect colleagues (different department)
7. Mindfulness
8. Other …

17 It should be mandatory for the hospital to organise support after an adverse event:
1. Yes
2. No

18 You have learned to cope with adverse events through/during:
1. Med-school (without clerkships)
2. Clerkships
3. Internships
4. Residency
5. Attending physician
6. Other study
7. Psychological help
8. Specific course/training
9. Peer-review
10. Mindfulness
11. Never learnt
12. Other…

19 In the course of your career, you have become more defensive:
1. Strongly disagree
2. Disagree
3. Agree
4. Strongly agree

For peer review only

20 Have you changed your working-conditions (e.g. less shifts, more diagnostic tests) after experiencing a patient-related adverse event?
1. Yes
2. No

21 What did you change? (More than one answer possible)
1. Work less
2. Less evening/night-shifts
3. No evening/night-shifts anymore
4. Calling a colleague sooner
5. Quit my job
6. More diagnostic tests
7. Start treatment sooner
8. Other…

22 Sometimes people experience traumatic events, such as live-threatening situations as a cause of a natural disaster, high-impact trauma or fire; being attacked or raped; witness a murder, death or hear find out someone close to them experienced something terrible. As a physician, one can experience such events in patient-care: critical illness or death of a patients, severe injury, as well as violent behaviour from a patient or their family.
Have you ever, during your work AS A PHYSICIAN experienced such (adverse) events/incidents?
1. Yes
2. No

23 Can you briefly describe the adverse event(s)?
1. Yes, please describe it…
2. No

24 Did (some part of) this/these adverse event(s) take place more than 4 weeks ago?
1. Yes
2. No

Followed by the TSQ questionnaire.

35 It might be that you did not experience the reactions and emotions as described on the previous pages, but that you recognize them from a past period in your life. For example, having disturbing thoughts or dreams, reliving the event, getting upset. Do you recognize these reactions?
1. Yes
2. No

36 Which of these reactions do you recognize? (More than one answer possible)
1. Disturbing thoughts
2. Disturbing dreams
3. Reliving experiences
4. Getting upset
5. Physical responses
6. Insomnia
7. Irritability
8. Trouble focussing
9. Hyper arousal
10. Tensed

37 How long did these symptoms last?
1. < 4 weeks

For peer review only

2. > 4 weeks
3. > 6 months
4. > 1 year

38 Have you ever been diagnosed with PTSD (posttraumatic stress-disorder)?
1. Yes
2. No

Followed by the HADS questionnaire.

53 Have you ever seriously considered quitting your job as a paediatrician? (e.g. by looking for a different job, talking to human resources about ending your contract?
1. Yes, absolutely
2. Yes, sometimes
3. No, never

54 What was the reason to consider quitting? (More than one answer possible)
1. Bad collaboration (working together) with a co-worker
2. Not challenging enough within the field
3. A lot of complications
4. Afraid to make mistakes
5. Too much responsibility
6. Traumatic experience on the work-floor
7. Complaint(s) from patients
8. One-sided
9. A lot of stress
10. A high workload
11. Disbalance between work and private life
12. Disagreements with work providers
13. Different interests
14. New challenge
15. Too much administration
16. Too much bureaucracy
17. Patient-violence
18. Too many rules
19. Insufficient guidance from supervisors
20. Problems with the partnership
21. Private reasons
22. Disutility
23. Other…

55 Did you ever faced a complaint at the disciplinary board?
1. Yes
2. No

Gynaecologists

1 Gender
1. Female
2. Male

2 Age
1. 25-34 years
2. 35-44 years

For peer review only

3. 45-54 years
4. 55-64 years
5. 65 years and older

3 Current position
1. Resident
2. Attending / fellow
3. Retired gynaecologist
4. Non-practicing/management

4 How many years have you been working as a gynaecologist? (Including working as a resident)

5 I considered quitting my job as a gynaecologist:
1. Strongly disagree
2. Disagree
3. Agree
4. Strongly agree

6 In case you considered quitting, what was the most important reason?
1. Most important reason: …
2. Never considered quitting

7 Did you ever face a complaint at the disciplinary board?
1. Yes
2. No

8 What do you consider to be an adverse event on the work-floor? (More than one answer possible)
1. Not applicable
2. Bad news conversations/interview
3. The critical moments when a patient's life is at risk
4. When a patient dies
5. When you miss a diagnosis
6. When you feel like you cannot help the patients
7. When you are in doubt whether you are making the right decision
8. Other …

9 After experiencing adverse events on the work-floor, I do the following: (More than one answer possible)
1. Not applicable
2. Seeking professional help
3. Going home as soon as possible
4. Drink more alcohol, use more drugs and smoke more cigarettes than I normally would do
5. Use new medication I barely or normally not use
6. Trying not to think about it
7. Find a distraction
8. Praying or other religious activities
9. Formal case review
10. Work out or doing my hobby
11. Talking to friends and/or family
12. Informally discussing the matter with peers/colleagues
13. I developed symptoms of a burn-out (e.g. emotional exhaustion, depersonalisation, lower self-esteem)
14. Call in sick
15. Quit my job
16. Other …

For peer review only

10 The current support organized by your institution after an adverse event is good:
1. Strongly disagree
2. Disagree
3. Agree
4. Strongly agree

11 There is plenty of room to informally discuss adverse events in the department/partnership:
1. Strongly disagree
2. Disagree
3. Agree
4. Strongly agree

12 You have learned to cope with adverse events through/during:
1. Clerkships
2. Residency
3. Specific course/training
4. Intervision
5. Other …
6. Never really learned it

13 After experiencing an adverse event I changed my working conditions (e.g. no more shifts, no more vaginal breech births, not performing any surgeries alone, coming to the hospital sooner instead of supervision over the telephone)
1. Strongly disagree
2. Disagree
3. Agree
4. Strongly agree

14 In the course of your career, you have become more defensive:
1. Strongly disagree
2. Disagree
3. Agree
4. Strongly agree

15 Support after an adverse event consisted of… (fill in the box)

16 There is a protocol at your institution regarding support after an adverse event:
1. No
2. I do not know
3. Yes, consisting of…

17 Your preferred support after an adverse event would be: (more than one answer possible)
1. Evaluation with direct colleagues
2. Intervision with indirect colleague's (physicians with a different specialty)
3. Talking to a psychologist or coach
4. A buddy
5. Other …

18 When you lay awake at night, you are not made for this job:
1. Strongly disagree
2. Disagree
3. Agree
4. Strongly agree

For peer review only

19 There should be a culture change regarding support after an adverse event:

1. Strongly disagree
2. Disagree
3. Agree
4. Strongly agree

20 Sometimes people experience traumatic events, such as live-threatening situations as a cause of a natural disaster, high-impact trauma or fire; being attacked or raped; witness a murder, death or hear find out someone close to them experienced something terrible. As a physician, one can experience such events in patient-care: critical illness or death of a patients, severe injury, as well as violent behaviour from a patient or their family.
1) Have you ever, during your work AS A PHYSICIAN experienced such (adverse) events/incidents?
2) After at least one of those adverse events, did you experience intense fear, helplessness or abomination?
3) Did this/these event(s) happened at least 4 weeks ago?

1. I answered yes to all of these questions
2. No, I did not answer yes to all these questions

Followed by the TSQ

31 Can you briefly describe the adverse event(s)?

32 It might be that you did not experience the reactions and emotions as described on the previous pages, but that you recognize them from a past period in your life. For example, having disturbing thoughts or dreams, reliving the event, getting upset. Do you recognize these reactions?

1. Yes
2. No

Followed by the HADS.

Othopaedic surgeons

1 What is your gender?

1. Male
2. Female

2 What is your age?

1. 20-29 years
2. 30-39 years
3. 40-49 years
4. 50-59 years
5. 60-69 years
6. ≥ 70 years

3 Are you a member of the Dutch Orthopaedic Society (NOV)?

1. Yes
2. No

4 What is your current position?

1. Resident
2. Attending physician
3. Retired
4. Non-practicing/management

For peer review only

5 How many years have you been working in the orthopaedic field as a physician? (Including as intern)

1. ≤ 5 years
2. 6-10 years
3. 11-15 years
4. 16-20 years
5. 21-25 years
6. 26-30 years
7. > 30 years

6 What do you consider to be an adverse event on the work-floor? (More than one answer possible)

1. Not applicable
2. Bad news conversation/interview
3. (Critically) ill patients
4. When a patient dies
5. When you miss a diagnosis
6. When you have to refuse a patient
7. When you are in doubt whether you are making the right decision
8. When an inexperienced colleague is on call
9. When you decide on an abstaining course of treatment
10. Treatment of young patients
11. When you know a patient will remain severely handicapped
12. Other …

7 How do you cope with adverse events on the work-floor? (More than one answer possible)

1. Not applicable / never experienced
2. Seeking professional help
3. Use new medication
4. Drink (more) alcohol
5. Smoke (more) cigarettes
6. Use (more) drugs
7. Work out (more)
8. Going home as soon as possible
9. Call in sick
10. Hide your emotions
11. Find a distraction
12. Praying or other religious activities
13. Talking to friends and/or family
14. Informally discussing the matter with peers/colleagues
15. Quitting
16. Work less
17. Other …

8 The current support organized by your institution after an adverse event is good:

1. Strongly disagree
2. Disagree
3. Agree
4. Strongly agree

9 There is plenty of room to informally discuss adverse events in the department/partnership:

1. Strongly disagree
2. Disagree
3. Agree
4. Strongly agree

For peer review only

10 There is plenty of room to informally discuss adverse events in the hospital:
1. Strongly disagree
2. Disagree
3. Agree
4. Strongly agree

11 There should be a culture change regarding support after an adverse event:
1. Strongly disagree
2. Disagree
3. Agree
4. Strongly agree

12 There is a protocol at your institution regarding support after an adverse event:
1. Yes
2. Yes, but nobody uses it
3. No, but there is one currently being created
4. No
5. I do not know

13 Support after an adverse event consisted of: (more than one answer possible)
1. I have never experienced an adverse event
2. There was none
3. Professionally organised peer-support
4. Self-initiated peer-support with direct colleagues (own department)
5. Self-initiated peer-support with indirect colleagues (different department)
6. Conversation(s) with a psychologist or coach
7. Evaluation with the present team
8. Help from the medical officer after a sick-leave
9. Other …

14 Your preferred support after an adverse event would be: (more than one answer possible)
1. Not applicable
2. Support is unnecessary
3. Professionally organised peer-support
4. Evaluation with the present team
5. Peer-support from direct colleagues (own department)
6. Peer-support from indirect colleagues (different department)
7. Mindfulness
8. Other …

15 It should be mandatory for the hospital to organise support after an adverse event:
1. Yes
2. No

16 You have learned to cope with adverse events through/during:
1. Med-school (without clerkships)
2. Clerkships
3. Internships
4. Residency
5. Attending physician
6. Psychological help
7. Specific course/training
8. Peer-review
9. Mindfulness
10. Never learnt

For peer review only

17 In the course of your career, you have become more defensive:
1. Strongly disagree
2. Disagree
3. Agree
4. Strongly agree

18 Have you changed your working-conditions (e.g. less shifts, more diagnostic tests) after experiencing a patient-related adverse event?
1. Yes
2. No

19 What did you change? (More than one answer possible)
1. Work less
2. Less evening/night-shifts
3. No evening/night-shifts anymore
4. Calling a colleague sooner
5. Quit
6. Start treatment sooner
7. Other…

20 Have you ever been diagnosed with PTSD (posttraumatic stress-disorder)?
1. Yes
2. No

21 Sometimes people experience traumatic events, such as live-threatening situations as a cause of a natural disaster, high-impact trauma or fire; being attacked or raped; witness a murder, death or hear find out someone close to them experienced something terrible. As a physician, one can experience such events in patient-care: critical illness or death of a patients, severe injury, as well as violent behaviour from a patient or their family.
Have you ever, during your work AS A PHYSICIAN experienced such (adverse) events/incidents?
1. Yes
2. No

22 Did this/these take place less than 4 weeks ago?
1. Yes
2. No

23 Can you describe the event/incident?
1. Yes
2. No

Followed by the TSQ and HADS questionnaire.

51 Have you ever seriously considered quitting your job as an orthopaedic surgeon? (e.g. by looking for a different job, talking to human resources about ending your contract?
1. Yes
2. No

52 What was the reason to consider quitting? (More than one answer possible)
1. Bad collaboration (working together) with a co-worker
2. Not challenging enough within the field
3. A lot of complication
4. Afraid to make mistakes
5. Too much responsibility

For peer review only

6. Traumatic experience on the work-floor
7. Complaint(s) from patients
8. One-sided
9. A lot of stress
10. A high workload
11. Disbalance between work and private life
12. Disagreements with work providers
13. Different interests
14. New challenge
15. Too much administration
16. Too much bureaucracy
17. Patient-violence
18. Too many rules
19. Insufficient guidance from supervisors
20. Problems with the partnership
21. Private reasons
22. Disutility
23. Other…

53 Did you ever face a complaint at the disciplinary board?

1. Yes
2. No

For peer review only

Supplementary file 3: table 5 – logistic regression model and odds ratios 'work experience – HADS-D and HADS-A'

HADS-D

	Estimate	Standard Error	Odds Ratio	z	p
(Intercept)	-3.314	0.416	0.036	-7.974	< .001
Work experience (5-10 years)	0.302	0.510	1.352	0.592	0.554
Work experience (10-15 years)	0.849	0.486	2.337	1.746	0.081
Work experience (15-20 years)	1.023	0.484	2.781	2.115	0.034
Work experience (20-25 years)	0.979	0.496	2.661	1.974	0.048
Work experience (25-30 years)	1.081	0.497	2.946	2.176	0.030
Work experience (≥ 30 years)	-0.385	0.615	0.681	-0.626	0.531

Model summary

Model	Deviance	AIC	BIC	df	X^2	p	McFadden R^2	Nagelkerke R^2	Tjur R^2
H_0	653.6	655.648	660.872	1371					
H_1	635.5	649.472	686.040	1365	18.176	0.006	0.028	0.035	0.033

HADS-A

	Estimate	Standard Error	Odds Ratio	z	p
(Intercept)	-1.508	0.198	0.221	-7.595	< .001
Work experience (5-10 years)	-0.179	0.263	0.836	-0.680	0.496
Work experience (10-15 years)	-0.361	0.281	0.697	-1.284	0.199
Work experience (15-20 years)	-0.164	0.279	0.848	-0.589	0.556
Work experience (20-25 years)	-0.347	0.299	0.707	-1.160	0.246
Work experience (25-30 years)	-0.461	0.315	0.631	-1.461	0.144
Work experience (≥ 30 years)	-1.116	0.341	0.328	-3.276	0.001

Model summary

Model	Deviance	AIC	BIC	df	X^2	p	McFadden R^2	Nagelkerke R^2	Tjur R^2
H_0	1093	1094.619	1099.843	1371					
H_1	1078	1092.140	1128.708	1365	14.479	0.025	0.013	0.019	0.000

For peer review only

STROBE 2007 (v4) Statement—Checklist of items that should be included in reports of *cross-sectional studies*

Section/Topic	Item #	Recommendation	Reported on page #
**Title and abstract**	1	(*a*) Indicate the study's design with a commonly used term in the title or the abstract	1 - 2
		(*b*) Provide in the abstract an informative and balanced summary of what was done and what was found	1 – 2
**Introduction**			
Background/rationale	2	Explain the scientific background and rationale for the investigation being reported	3 – 4
Objectives	3	State specific objectives, including any prespecified hypotheses	4
**Methods**			
Study design	4	Present key elements of study design early in the paper	4 - 5
Setting	5	Describe the setting, locations, and relevant dates, including periods of recruitment, exposure, follow-up, and data collection	4
Participants	6	(*a*) Give the eligibility criteria, and the sources and methods of selection of participants	4
Variables	7	Clearly define all outcomes, exposures, predictors, potential confounders, and effect modifiers. Give diagnostic criteria, if applicable	5
Data sources/ measurement	8*	For each variable of interest, give sources of data and details of methods of assessment (measurement). Describe comparability of assessment methods if there is more than one group	4
Bias	9	Describe any efforts to address potential sources of bias	*5*
Study size	10	Explain how the study size was arrived at	-
Quantitative variables	11	Explain how quantitative variables were handled in the analyses. If applicable, describe which groupings were chosen and why	5
Statistical methods	12	(*a*) Describe all statistical methods, including those used to control for confounding	5-6
		(*b*) Describe any methods used to examine subgroups and interactions	5-6
		(*c*) Explain how missing data were addressed	5-6
		(*d*) If applicable, describe analytical methods taking account of sampling strategy	-
		(*e*) Describe any sensitivity analyses	-
**Results**			

For peer review only

Participants	13*	(a) Report numbers of individuals at each stage of study—eg numbers potentially eligible, examined for eligibility, confirmed eligible, included in the study, completing follow-up, and analysed	7
		(b) Give reasons for non-participation at each stage	7
		(c) Consider use of a flow diagram	-
Descriptive data	14*	(a) Give characteristics of study participants (eg demographic, clinical, social) and information on exposures and potential confounders	7
		(b) Indicate number of participants with missing data for each variable of interest	7
Outcome data	15*	Report numbers of outcome events or summary measures	8-10
Main results	16	(*a*) Give unadjusted estimates and, if applicable, confounder-adjusted estimates and their precision (eg, 95% confidence interval). Make clear which confounders were adjusted for and why they were included	8-10
		(*b*) Report category boundaries when continuous variables were categorized	-
		(*c*) If relevant, consider translating estimates of relative risk into absolute risk for a meaningful time period	-
Other analyses	17	Report other analyses done—eg analyses of subgroups and interactions, and sensitivity analyses	8-10
**Discussion**			
Key results	18	Summarise key results with reference to study objectives	10-12
Limitations	19	Discuss limitations of the study, taking into account sources of potential bias or imprecision. Discuss both direction and magnitude of any potential bias	13
Interpretation	20	Give a cautious overall interpretation of results considering objectives, limitations, multiplicity of analyses, results from similar studies, and other relevant evidence	14
Generalisability	21	Discuss the generalisability (external validity) of the study results	13
**Other information**			
Funding	22	Give the source of funding and the role of the funders for the present study and, if applicable, for the original study on which the present article is based	17

*Give information separately for cases and controls in case-control studies and, if applicable, for exposed and unexposed groups in cohort and cross-sectional studies.

Note: An Explanation and Elaboration article discusses each checklist item and gives methodological background and published examples of transparent reporting. The STROBE checklist is best used in conjunction with this article (freely available on the Web sites of PLoS Medicine at http://www.plosmedicine.org/, Annals of Internal Medicine at http://www.annals.org/, and Epidemiology at http://www.epidem.com/). Information on the STROBE Initiative is available at www.strobe-statement.org.

For peer review only

BMJ Open

Potential traumatic events in the workplace and depression, anxiety and posttraumatic stress: a cross-sectional study among Dutch gynaecologists, paediatricians and orthopaedic surgeons.

Journal:	*BMJ Open*
Manuscript ID	bmjopen-2019-033816.R1
Article Type:	Original research
Date Submitted by the Author:	07-Feb-2020

Complete List of Authors:	Scheepstra, Karel; Amsterdam UMC - Locatie AMC, Psychiatry<br>Pauw, Hannah; Amsterdam UMC - Locatie AMC, Faculty of Medicine<br>van Steijn, Minouk; Onze Lieve Vrouw Hosp, Obstetrics and Gynaecology; Amsterdam UMC - Locatie AMC, Obstetrics and Gynaecology<br>Stramrood, Claire; OLVG Locatie Oost, Obstetrics and Gynaecology<br>Olff, Miranda; Amsterdam UMC - Locatie AMC, Psychiatry; Arq Psychotrauma Research<br>van Pampus, Maria G.; Onze Lieve Vrouw Hosp, Obstetrics and Gynaecology

**Primary Subject Heading**:	Mental health
Secondary Subject Heading:	Occupational and environmental medicine, Health services research
Keywords:	OCCUPATIONAL & INDUSTRIAL MEDICINE, PSYCHIATRY, Depression & mood disorders < PSYCHIATRY, Anxiety disorders < PSYCHIATRY, Adverse events < THERAPEUTICS, Protocols & guidelines < HEALTH SERVICES ADMINISTRATION & MANAGEMENT

SCHOLARONE™
Manuscripts

I, the Submitting Author has the right to grant and does grant on behalf of all authors of the Work (as defined in the below author licence), an exclusive licence and/or a non-exclusive licence for contributions from authors who are: i) UK Crown employees; ii) where BMJ has agreed a CC-BY licence shall apply, and/or iii) in accordance with the terms applicable for US Federal Government officers or employees acting as part of their official duties; on a worldwide, perpetual, irrevocable, royalty-free basis to BMJ Publishing Group Ltd ("BMJ") its licensees and where the relevant Journal is co-owned by BMJ to the co-owners of the Journal, to publish the Work in this journal and any other BMJ products and to exploit all rights, as set out in our licence.

The Submitting Author accepts and understands that any supply made under these terms is made by BMJ to the Submitting Author unless you are acting as an employee on behalf of your employer or a postgraduate student of an affiliated institution which is paying any applicable article publishing charge ("APC") for Open Access articles. Where the Submitting Author wishes to make the Work available on an Open Access basis (and intends to pay the relevant APC), the terms of reuse of such Open Access shall be governed by a Creative Commons licence – details of these licences and which Creative Commons licence will apply to this Work are set out in our licence referred to above.

Other than as permitted in any relevant BMJ Author's Self Archiving Policies, I confirm this Work has not been accepted for publication elsewhere, is not being considered for publication elsewhere and does not duplicate material already published. I confirm all authors consent to publication of this Work and authorise the granting of this licence.

For peer review only

Potential traumatic events in the workplace and depression, anxiety and posttraumatic stress: a cross-sectional study among Dutch gynaecologists, paediatricians and orthopaedic surgeons.

For peer review only

Authors

Karel W. F. Scheepstra*, MD[1]
Hannah S. Pauw, BSc[2]
Minouk E. van Steijn, MD[3,4]
Claire A.I. Stramrood, MD PhD[4]
Miranda Olff, PhD[1,5]
Maria G. van Pampus, MD PhD[4]

1. Amsterdam UMC, University of Amsterdam, Department of Psychiatry, Meibergdreef 9, 1105 AZ, Amsterdam, the Netherlands.
2. Amsterdam UMC, University of Amsterdam, Faculty of Medicine, Meibergdreef 9, 1105 AZ, Amsterdam, the Netherlands.
3. Amsterdam UMC, University of Amsterdam, Department of Obstetrics and Gynaecology, Meibergdreef 9, 1105 AZ, Amsterdam, the Netherlands.
4. OLVG, Department of Obstetrics and Gynaecology, Oosterpark 9, 1090 HM, Amsterdam, the Netherlands
5. Arq Psychotrauma Expert Group, Nienoord 5, 1112 XE, Diemen, the Netherlands

***Corresponding author:**
K.W.F. Scheepstra
Department of Psychiatry
Meibergdreef 9
1105 AZ Amsterdam
The Netherlands
k.w.scheepstra@amsterdamumc.nl
+31 (0) 20 8913500

ABSTRACT

Objective: To compare the prevalence of work-related potential traumatic events, support protocols and mental health symptoms across Dutch gynaecologists, orthopaedic surgeons and paediatricians.

Design: Cross-sectional study, supplementary analysis of combined data.

Setting: Nationwide survey between 2014 and 2017.

Participants: An online questionnaire was sent to all Dutch gynaecologists, orthopaedic surgeons and paediatricians, including resident physicians (4959 physicians). 1374 questionnaires were eligible for analysis, corresponding with a response rate of 27.7%.

Outcome measures: Primary outcome measures are the prevalence of work-related potential traumatic events (PTEs), depression, anxiety, psychological distress and traumatic stress, measured with validated screening instruments (Hospital Anxiety and Depression Scale, HADS; Trauma Screening Questionnaire, TSQ). Secondary outcomes are the association of mental health and defensive practice to traumatic events and support protocols.

Results: Of the respondents, 20.8% experienced a work-related potential traumatic event at least four weeks ago. Prevalence rates indicative of depression, anxiety or posttraumatic stress disorder were respectively 6.4%, 13.6% and 1.5%. Comparing female versus male attendings, depression (9.2% vs. 5.2%, p=0.019), anxiety (18.2% vs. 8.2%, p<0.001) and psychological distress (22.8% vs. 12.5%, p<0.001), were significantly more prevalent in women. The absence of a support protocol was significantly associated with more probable posttraumatic stress disorder (p=0.022).

Conclusion: Physicians are frequently exposed to potential traumatic events with high emotional impact over the course of their career. Lacking a support protocol after adverse events was associated with more posttraumatic stress. Adverse events were associated with more dissatisfaction with work and a more defensive practice. More awareness must be created for the mental health of physicians as well as for the implementation of a well-organised support system after potential traumatic events.

Word count: 263

Keywords: Occupational & Industrial Medicine, Psychiatry, Depression & mood disorders, Anxiety disorders, Adverse Events, Protocols & guidelines

ARTICLE SUMMARY

Strengths and limitations of this study

- This is one of the few studies done on PTSD in physicians, in particular with regard to mental health in relation to social and occupational functioning and clinical practice related consequences.
- This study incorporated the A and F criterion in our probable PTSD diagnosis, therefore the prevalence is more likely to reflect the real prevalence of PTSD.
- One limitation is that the population of this study is limited to three specialties and one country in Europe and thus it is difficult to translate these results to physicians worldwide.

For peer review only - http://bmjopen.bmj.com/site/about/guidelines.xhtml

INTRODUCTION

Physicians are frequently exposed to medical adverse events, such as life-threatening situations, illnesses, grief, death, and patient violence and aggression. Dealing with such events makes the medical profession one with occupational health hazards, comparable with fire fighters, rescue workers, military and police personnel.[1–3] The support for physicians is, however, underdeveloped in most areas of the medical profession compared to other professions.[4,5] It is generally assumed that health care providers have adequate coping mechanisms to deal with such work stressors. However, our previous work showed that most gynaecologists, paediatricians and midwives report to experience support and protocols after work-related potential traumatic events as insufficient. Dutch paediatricians report ´missing a diagnosis´ as having the most emotional impact and ´aggressive behaviour from parents´ as the most common adverse event. [6] Midwives working in primary care reported higher levels of anxiety compared with their colleagues working in secondary or tertiary care.[7] Gynaecologists report that development of coping strategies with work-related stressors are not educated during- or part of medical and specialist training.[8] Physician health is one of the competencies described in the CanMEDS framework used in Dutch medical education.[9] This framework recognises that, to provide optimal patient care, physicians must take responsibility for their own well-being and that of their colleagues. Several hospitals have started peer-support programmes, as this was shown to help physicians dealing with adverse events.[10]

Depression, anxiety and posttraumatic stress disorder

Major depressive disorder (MDD) is a common affective disorder that is characterised by a depressed mood and/or loss of pleasure.[11] A review on the epidemiology of mental disorders in Europe describes a one-year prevalence of 6.9% in the general population[12]; the Nemesis-2 study showed a one-year prevalence of mood disorders of 3.0% among Dutch citizens with a high income.[13] A meta-analysis on depressive symptoms and MDD among resident physicians showed high prevalence rates (20.9% to 43.2%).[14] Fewer studies were done on depressive symptoms among medical specialists, showing prevalence rates from 10.3 to 28.0%.[15–18] A recent literature review showed

For peer review only - http://bmjopen.bmj.com/site/about/guidelines.xhtml

that as many as 40 per 100,000 physicians die by suicide each year in the United States of America, more than double the rate of the general population.[19]

Anxiety disorders include many different disorders, ranging from a specific phobia to a generalised anxiety disorder. The one-year prevalence for anxiety disorders in the Netherlands is 6.0% among citizens with a high income and 10.1% among citizens with a low income.[13]
PTSD is a highly debilitating disorder that can develop after exposure to a psychotraumatic event.[20] Symptoms of PTSD include intrusions, avoidance of stimuli related to the trauma, alterations in cognition or mood, and hyperarousal. The point prevalence of posttraumatic stress symptoms among physicians ranges between 3.8% and 15.0.[15,21–25]

The main objective of this supplementary analysis is to compare the prevalence of potential traumatic events (PTEs) and related mental health between a large group of gynaecologists, paediatricians and orthopaedic surgeons and show differences between gender, age and position. Additionally, this study investigates mental health in relation to social and occupational functioning and clinical practice related consequences. Combining the data of previous work allows us to gain statistical power to explore the association between support protocols and mental health symptoms in a larger group.

METHODS

Population and procedure

Each physician received one invitation for an online questionnaire (supplementary file 1) and two reminders over an 8-week period. The questionnaire was sent through Thesistools® or Surveymonkey®, creating an anonymous (non-traceable) e-mail link. The questionnaire was sent to 1589 gynaecologists, 2160 paediatricians and 1210 orthopaedic surgeons, with a total of 4959 physicians, including residents. The invitation to participate in the questionnaire for the gynaecologists was sent in 2014, orthopaedic surgeons in 2016 and paediatricians in 2017. This is an additional analysis of combining data of previous work.

For peer review only - http://bmjopen.bmj.com/site/about/guidelines.xhtml

The membership databases of the Dutch Society of Obstetrics & Gynaecology (NVOG), Dutch Society of Orthopaedic Surgeons (NOV) and the Dutch Society of Paediatricians (NvK) were used. Registration as a medical specialist or resident in one of these societies is obligatory and so all Dutch medical specialists in these fields were contacted. These databases contained residents, attending, retired and non-practicing physicians.

This study was exempted from ethical approval by the Medical Research Ethics Committees United (MEC-U), and registered under numbers W17.169 and W18.096.

For peer review only

Patient and public involvement

Engagement with the described medical societies was vital to ensure participation and a high response rate. The qualitative questions were piloted among several medical specialists of each specific medical specialty.

Measurements

The instruments included the Hospital Anxiety and Depression Scale (HADS) and the Trauma Screening Questionnaire (TSQ) as validated screening instruments. The HADS is a 14-item screening instrument for depression and anxiety, where the subscales contain seven questions each. The cut-off value of the Dutch version of the depression (HADS-D) and anxiety subscale (HADS-A) is equal to or higher than eight. The total HADS cut-off value for general distress is equal or higher than 12, corresponding to clinically relevant psychological distress; this is a rather non-specific but sensitive measurement to screen for mental disorders.[26] The sensitivity and specificity for both HADS-A and HADS-D are approximately 0.80, with Cronbach's alphas for HADS-A from 0.68 to 0.93 (mean 0.83) and for HADS-D from 0.67 to 0.90, in different populations. [27]

The TSQ is a ten-item screening instrument for a probable diagnosis of PTSD according to the DSM-IV, with a sensitivity of 0.94 and specificity of 0.56 when using a cut-off of six and higher. The Dutch

For peer review only - http://bmjopen.bmj.com/site/about/guidelines.xhtml

psychometric properties are validated with Cronbach's alphas from 0.71 to 0.91.[28] Only respondents who answered 'yes' to experiencing a work-related traumatic event at least four weeks ago were asked to fill out the TSQ.

Additionally, the survey contained questions about demographics, personal experiences, coping and support strategies after adverse events in the workplace. Regarding the latter, multiple-choice options were given as well as an open field where respondents were able to add their individual answers or comment on their experience. These qualitative questions were piloted among several medical specialists of each specific medical specialty, to increase face value of the questions (supplementary file 1).

Statistical analysis

Statistical analysis was performed using IBM Statistical Package for the Social Sciences (SPSS) version 22 and JASP version 0.8.5.1. Demographic data and multiple-choice questions were analysed using descriptive statistics and exported as frequency tables and bar charts. Differences in outcomes (in various groups) for categorical variables were tested using either a Chi-squared test or a Fisher's exact test where applicable. A two-sided p-value equal to or less than 0.05 was considered statistically significant. A multivariable logistic regression analysis was performed to estimate the effects of age, work-experience, gender and position on the likelihood that participants develop mental health outcomes, a p-value equal to or less than 0.05 was considered statistically significant. Multiple testing corrections were not performed.

For peer review only - http://bmjopen.bmj.com/site/about/guidelines.xhtml

RESULTS

A total of 1374 questionnaires were collected with a response rate of 27.7%. Table 1 shows the respondents characteristics by specialty.

Table 1: Respondent characteristics

	Total (n=4959)	Gynaecologists (n=1589)	Orthopaedic surgeons (n=1210)	Paediatricians (n=2160)
**Response rate**	27.7	48.9	21.3	29.8
**Gender**				
Male	44.9	34.7	85.6	32.7
Female	55.1	65.3	14.4	67.3
**Position**				
Resident	23.0	26.9	20.9	18.0
Attending physician	67.2	64.7	62.3	74.9
Retired	7.1	5.4	13.4	5.6
Non-practicing	2.6	3.0	3.4	1.5
**Years in practice**				
≤ 5	12.4	14.3	12.0	9.8
6-10	18.6	19.0	19.5	17.3
11-15	15.8	17.7	14.0	13.9
16-20	14.3	11.6	13.4	19.3
21-25	12.4	12.8	9.2	13.9
26-30	11.3	9.5	14.4	12.0
>30	15.1	14.7 *	17.5	13.9
**Complaints disciplinary board**	19.9	21.0	29.8	11.2
**Support protocol**				
Yes	19.6	15.8	18.8	26.3
No	42.4	52.7	30.1	34.1
I do not know	38.0	31.4	51.0	39.5

All values shown as %
*=2 missing

The majority of the orthopaedic surgeons were male (85.6%) while in the other specialties the majority were female. In all specialties the majority of the respondents were attending physicians. The gender and position distributions were compared with the membership databases of the societies, and for none of the specialties the distribution in the sample was significantly different from the population. The orthopaedic surgeons reported to have had the most complaints at a disciplinary board (29.8%). One hundred eight (26.3%) paediatricians reported to have a support protocol, whereas only 18.8% of the orthopaedic surgeons and 15.8% of the gynaecologists report to have a protocol after adverse events.

Outcomes of the HADS by specialty are shown in table 1.

For peer review only - http://bmjopen.bmj.com/site/about/guidelines.xhtml

Table 2: Depression, anxiety, psychological distress and PTSD by specialty

	Total (n=1374)	Gynaecologists (n=672)	Orthopaedic Surgeons (n=292)	Paediatricians (n=410)
**Depression**	6.4	6.5	4.8	7.3
**Anxiety**	13.6	15.8	8.2	14.1
**Psychological distress**	17.1	18.2	12.0	19.3
**PTSD**				
PTE ≥ 4 weeks ago	20.8	12.8	19.5	34.9
PTS symptoms earlier	13.4	8.2	8.6	25.4
TSQ	1.5	1.5	0.3	2.2

All values shown in % scored above HADS or TSQ cut off
Abbreviations: Potential Traumatic Event (PTE); Trauma Screening Questionnaire (TSQ); Hospital Anxiety and Depression Scale (HADS); Posttraumatic stress (PTS); Posttraumatic stress disorder (PTSD).

Table 3: Depression, anxiety and psychological distress by position (resident vs. attending) and gender

	Total (n=1240)	Depression	p	χ^2	Anxiety	p	χ^2	Psycho-logical distress	p	χ^2
**Gender**										
Male	40.8	5.5	0.075	3.2	10.1	**<0.001**	**17.2**	14.2	**<0.001**	**12.8**
Female	59.2	8.2			18.7			22.3		
**Position**										
Resident	25.4	4.7	0.112	2.5	17.1	0.125	2.3	18.4	0.887	0.0
Attending	74.6	7.4			13.6			18.1		
**Residents**										
Male	25.6	3.7	0.346	0.9	13.6	0.053	3.7	14.8	0.058	3.6
Female	74.4	5.1			18.3			19.6		
**Attending**										
Male	45.9	5.2	**0.008**	**7.1**	8.2	**<0.001**	**25.7**	12.5	**<0.001**	**22.2**
Female	54.1	9.2			18.2			22.8		

All values shown as % scored above cut-off HADS
Abbreviations: Hospital Anxiety and Depression Scale (HADS)

Eighty eight (6.4%) respondents scored above the cut-off value for depression and one hundred and eighty eight (13.6%) respondents scored above the cut-off value for anxiety. Two hundred and thirty six (17.1%) scored above the cut-off value of the total HADS, corresponding to clinically relevant psychological distress. When comparing specialties, there is no significant difference in depression.

For peer review only - http://bmjopen.bmj.com/site/about/guidelines.xhtml

However, looking at the prevalence of anxiety and psychological distress, the orthopaedic surgeons scored significantly lower compared to both gynaecologists and paediatricians.

As seen in table 3, no significant difference was found between male and female residents. When comparing all male versus female physicians, the prevalence rates of anxiety (10.1% vs. 18.7%, p<0.001) and the total HADS indicating psychological distress (14.2% vs. 22.3%, p<0.001) were significantly higher among female physicians. When comparing male and female attendings, the rates of depression (5.2% vs. 9.2%, p=0.019), anxiety (8.2% vs. 18.2%, p<0.001) and the total HADS (12.5·0% vs. 22.8%, p<0.001) were significantly higher among female attendings. The highest rate of respondents with a score above the cut-off value for anxiety were found among physicians with 6-10 years in practice (supplementary file 2). There were no differences in primary outcomes when comparing residents with attendings. There was a significant increase in the prevalence of depression in the groups with more years in practice (OR 2.95, p=0.006, supplementary file 2), but not with increased age. We found a significant decrease in the prevalence of anxiety with more years in practice (OR 0.33, p=0.025, supplementary file 2).

Posttraumatic stress disorder

Two hundred eighty six respondents (20.8%) reported having experienced at least one potential traumatic event (PTE) during their work, at least four weeks ago, thereby meeting DSM-IV criterion A and F. Of these respondents, 183 reported recognising posttraumatic stress (PTS) symptoms after this event (13.4% of the total group, 64.3% of the potentially traumatised group) and 20 screened positive for probable PTSD (1.5% of the total group, 7.0% of the potentially traumatised group, table 2). Of the paediatricians 34.9% reported having experienced a PTE at least four weeks ago. This is significantly higher than the percentages of the gynaecologists (12.8%, p<0.0001) and orthopaedic surgeons (19.5%, p<0.0001). When comparing the prevalence of probable PTSD, the paediatricians had a significant higher prevalence compared to the orthopaedic surgeons (2.2% vs. 0.3%, p=0.041). Both the paediatricians and gynaecologists report to recognise more PTS symptoms. When comparing gender and position (resident vs. attending, table 4), residents are less likely to have experienced a

For peer review only - http://bmjopen.bmj.com/site/about/guidelines.xhtml

PTE (16.8% vs. 22.6%, p=0.030) and thus also report significantly less PTS symptoms earlier (7.9% vs. 15.6%, p<0.001).

For peer review only

For peer review only - http://bmjopen.bmj.com/site/about/guidelines.xhtml

Table 4: Posttraumatic stress outcomes by position (resident vs. attending) and gender

	Total (n=1240)	PTE	p	χ^2	PTS symptoms earlier	p	χ^2	PTSD	p	χ^2
**Gender**										
Male	40.8	21.3	0.878	0.0	11.3	**0.044**	**4.1**	1.2	0.516	0.4
Female	59.2	21.0			15.3			1.6		
**Position**										
Resident	25.4	16.8	**0.030**	**4.7**	7.9	**<0.001**	**11.6**	0.9	0.391	0.7
Attending	74.6	22.6			15.6			1.6		
**Residents**										
Male	25.6	21.0	0.245	1.3	4.9	0.246	1.3	0.0	0.306	1.0
Female	74.4	15.3			8.9			1.3		
**Attending**										
Male	45.9	21.4	0.377	0.8	12.5	**0.006**	**7.6**	1.4	0.371	0.8
Female	54.1	23.6			18.2			1.8		

All values shown as %, PTSD is shown as scored above cut-off TSQ
Abbreviations: Potential Traumatic Event (PTE); Trauma Screening Questionnaire (TSQ); Hospital Anxiety and Depression Scale (HADS); Posttraumatic stress (PTS); Posttraumatic stress disorder (PTSD).

The highest rate of probable PTSD was found among attendings with 15 respondents (1.6%) compared to 3 of the residents (0.9%). When comparing gender, male and female physicians report the same number of PTEs, but female physicians are more likely to report earlier PTS-symptoms (15.3% vs. 11.3%, p=0.044). Those that witnessed a PTE, reported significantly more defensive work changes after an incident (28.0% vs 20.5%, p=0.007), but they did not consider to quit as a physician more often (37.5% vs 35.0%, p=0.45). However, those that recognised PTS-symptoms after an incident did consider quitting work more often (42.6% vs. 29.2%, p=0.029). Those with current probable PTSD became more defensive (45.0% vs. 26.7%, trend, p=0.079) and significantly considered quitting medical work more often (60.0% vs. 35.8%, p=0.032).

When evaluating the presence of a support protocol, this was found not to influence the primary mental health outcomes, except for probable PTSD. Participants who responded that there was no

For peer review only - http://bmjopen.bmj.com/site/about/guidelines.xhtml

support protocol at their work-place after an adverse event, were significantly more likely to report probable PTSD after a PTE (p=0.022).

DISCUSSION

Depression and anxiety

This additional analysis combined data from previous work to compare the point prevalence rates of probable depression, anxiety, psychological distress, work-related PTE and PTSD among gynaecologists, orthopaedic surgeons and paediatricians. It is part of the Work-related Adverse and Traumatic Events Research (WATER) and this paper combines the results of previous studies [6,8], to gain statistical power and show differences between support protocols, specialties, gender and position. Our point prevalence rates of depression (6.4%) and anxiety (13.6%), are high compared to 12-month prevalence rates of mood disorders (3.0%) and anxiety (6.0%) in high income populations in the Netherlands, and compared to European 12-month prevalence rate of anxiety (6.4%).[12,13] The point prevalence rates in this study are comparable to American 12-month general prevalence rates of depression (6.7%) and anxiety (18.1%).[29,30] This indicates that physicians are more depressed and anxious compared to the general population and the population with high income in the Netherlands. This high prevalence is in line with current literature, where even higher prevalence rates for depression and anxiety were found among medical students, residents and specialists.[14,15,31] One of the relevant findings of this study is the high prevalence of psychological distress (17.6%). Scoring above this cut-off is not specific for one diagnosis, but does indicate a high level of distress and a risk for potential unfit physicians. We found a significant increase in the prevalence of depression corresponding with more years in practice. Furthermore, this study showed a significantly higher prevalence of depression and anxiety among female compared to male attendings. This finding correlates with previous study results,[32] where prevalence rates for depression and anxiety were higher among British female doctors compared to males. These findings are also in line with epidemiologic data on the gender distribution of mood and anxiety disorders in the general population.[12,13] This finding is relevant, because of the feminization of medical specialties globally, and an increase in female physicians of 1.0 to 2.0 % each year in the last ten years in the

For peer review only - http://bmjopen.bmj.com/site/about/guidelines.xhtml

Netherlands.[33] These results may implicate that a change is needed in support after adverse events, that is sensitive to the increase in women working in the field of medicine.

Posttraumatic stress disorder

In this sample, 285 respondents (20.8%) reported to have experienced a potential traumatic event (PTE) that took place longer than four weeks ago. This is within the range of the estimated prevalence of second victims of 10.4% and 43.3%, reported by Seys et al.[34] Our prevalence for work-related PTSD (1.5%) is slightly higher, compared to the general Dutch prevalence of PTSD (1.2%).[35] The Dutch prevalence of only work-related PTSD is not known, however assumed to be lower. Of the potential traumatised respondents, 7.0% screened positive for PTSD, which is comparable to other occupations with mental health hazards, such as police officers after critical police incidents (7.0%).[36]

One of the key findings of this supplementary analysis, is that after experiencing a PTE, the absence of a support protocol is significantly associated with more probable PTSD (p=0.022). This once again shows the importance of support protocols and implicates that a well perceived support system may prevent posttraumatic stress complaints. This survey did however not explore the types of support protocols (e.g. debriefing versus peer-support) in place, as they may differ across hospitals and even within one hospital across specialties. Therefore nothing can be concluded of the type of support protocol that may help prevent PTS symptoms. However, some form of formalised support may lead to seeking- and finding professional help earlier. Studies have shown that the threshold to look for professional mental health as a physician is high. Physicians report a reluctance to seek care, due to barriers such as too little time, fear of losing their medical license and stigma.[37] A (semi) compulsory support protocol after a PTE could potentially increase awareness, lower this threshold and guide physicians in need towards professional support or treatment. Debriefing and hospital based peer-support have shown useful and effective after PTEs in several small studies. [10,38] Also, this awareness of more workplace support may lead to perceiving adverse events less psychotraumatic, however having a support protocol was not correlated with less PTEs.

For peer review only - http://bmjopen.bmj.com/site/about/guidelines.xhtml

Of the residents who filled out the TSQ, three screened positive for PTSD (all female respondents). These findings are in line with the known gender distribution, as women are more likely to develop PTSD than men.[39] Other studies found higher prevalence rates of PTSD (i.e. Joseph[21] = 15%, Mills[24] = 11.9%, Ruitenburg[15] =15%, and Shi[22] = 3.8%), but they used different (non-) validated screening instruments and methods. None of these screening instruments assessed when the PTE happened (for a diagnosis of PTSD this needs to be at least four weeks ago), meaning that their respondents may not meet criterion A of PTSD and could also be suffering from acute stress symptoms. Furthermore, these studies did not assess whether the potential event was work-related, so these numbers show general PTSD prevalence rates which are usually higher.

Studies have shown that dysfunctional coping can predict complicated grief and PTSD severity.[40,41] Thus, it is important to educate physicians about coping mechanisms and stress management techniques to prevent adverse mental health conditions.[42] With social support being an important protective factor for PTSD[43], the role of educating health care professionals to address this problem and support their colleagues in peer-support groups is crucial.[10]

Job related consequences of depression, anxiety, adverse events and PTSD

This study shows that physicians consider quitting their job more often, after having experienced PTS-symptoms in the past or currently suffering from probable PTSD. Experiencing a PTE is also associated with more defensive medical practice. When looking at the prevalence of complaints at a disciplinary board, the orthopaedic surgeons reported the most complaints (table 1). This may be due to the higher amount of elective work they do, which usually leads to more dissatisfied patients, compared to emergency work. More complaints was not associated with considering quitting work or increased rates of depression, anxiety or psychological distress.

For peer review only

For peer review only - http://bmjopen.bmj.com/site/about/guidelines.xhtml

Strengths

Little research has been done on PTSD in physicians, in particular with regard to mental health in relation to social and occupational functioning and clinical practice related consequences. We incorporated the A and F criterion in our probable PTSD diagnosis, therefore our prevalence is more likely to reflect the real prevalence of PTSD. Also, this study included a large number (n=1374) of physicians, which made it possible to analyse gender, position and age differences. The overall response rate among gynaecologists was high (42.9%), followed by the orthopaedic surgeons (29%) and the paediatricians (18.9%).

Limitations

There are several limitations to this study. The population of this study is limited to three specialties and one country in Europe and thus it is difficult to translate these results to physicians worldwide. However, it is known that working conditions and related stress levels are comparable with the rest of Europe and North-America. Also, prevalence rates reported in this study are possibly not comparable to other studies on mental health, as many different samples and measurements are used in mental health studies. Furthermore, avoidance is one of the key symptoms of PTSD and can therefore lead to non-response and non-completion of the survey, as it may trigger symptoms of PTSD. Therefore the prevalence rates found in psychotrauma studies are often an underestimation of the PTSD-prevalence. On the other hand, this self-selection bias may also lead to overrepresentation of physicians with strong opinions. One suggestion to decrease this non-completion bias, is to give respondents the opportunity to finish the survey at a later time point and receive a reminder. The overall response rate was 27.7%, which is slightly higher than the average response rate for email surveys (25.0%)[44] and comparable to similar surveys in this field [45]. Due to the low numbers of PTSD, it was not possible to correlate the type of adverse event with more PTSD. Furthermore, the HADS and TSQ are validated screening instruments, however they only give an estimate of the prevalence rates of probable disorders as they do not cover all the symptom domains of the disorders. Only the prevalence of PTSD is work-related, the numbers for depression and anxiety are general prevalence rates.

For peer review only - http://bmjopen.bmj.com/site/about/guidelines.xhtml

For peer review only

CONCLUSION

Physicians are often exposed to PTEs over the course of their career with potentially high emotional impact. The prevalence rates for probable depression (6.4%) and anxiety (13.6%) were higher among physicians, compared to the general population with high income in the Netherlands. The point prevalence of clinical psychological distress was 19.0% and of probable PTSD was 1.5%. Female physicians are significantly more prone to develop depressive- and anxiety disorders than their male colleagues. PTEs were associated with more defensive practice and dissatisfaction about work. And importantly, the absence of a support protocol was associated with more probable PTSD. This once again shows the importance of support protocols and implicates that a well perceived support system, such as peer support and debriefings, may prevent posttraumatic stress complaints and unfit physicians.

For peer review only

For peer review only - http://bmjopen.bmj.com/site/about/guidelines.xhtml

ACKNOWLEDGEMENTS

We would like to thank the Dutch Society of Obstetrics & Gynaecology (NVOG), Dutch Society of Orthopaedic Surgeons (NOV), the Dutch Society of Paediatricians (NvK), and their members, for their cooperation in this study.

For peer review only

COMPETING INTERESTS

None of the authors declare a conflict of interest.

For peer review only

FUNDING

This research received no specific grant from any funding agency in the public, commercial or not-for-profit sectors.

For peer review only

For peer review only - http://bmjopen.bmj.com/site/about/guidelines.xhtml

CONTRIBUTORSHIP

KS, MvS, CS, MvP were involved in the design of this study. KS and HP analysed the data and made the first draft. KS, HP, MvS, CS, MO and MvP were involved in finalising the manuscript.

For peer review only

For peer review only - http://bmjopen.bmj.com/site/about/guidelines.xhtml

DATA AVAILABILITY

The data can be made available by the corresponding author upon reasonable request and for a well defined purpose. The request will be discussed in our research group. The data will be de-identified participant data. A statistical analysis plan is available.

For peer review only

For peer review only - http://bmjopen.bmj.com/site/about/guidelines.xhtml

REFERENCES

1 Yehuda R, Vermetten E, McFarlane AC, *et al.* PTSD in the military: Special considerations for understanding prevalence, pathophysiology and treatment following deployment. *Eur J Psychotraumatol* Published Online First: 2014. doi:10.3402/ejpt.v5.25322

2 van der Meer CAI, Bakker A, Schrieken BAL, *et al.* Screening for trauma-related symptoms via a smartphone app: The validity of Smart Assessment on your Mobile in referred police officers. *Int J Methods Psychiatr Res* Published Online First: 2017. doi:10.1002/mpr.1579

3 Mellotte H, Murphy D, Rafferty L, *et al.* Pathways into mental health care for UK veterans: a qualitative study. *Eur J Psychotraumatol* Published Online First: 2017. doi:10.1080/20008198.2017.1389207

4 Edrees H, Federico F. Supporting clinicians after medical error. *BMJ* 2015. doi:10.1136/bmj.h1982

5 Devi S. Doctors in distress. *Lancet* Published Online First: 2011. doi:10.1016/S0140-6736(11)60145-1

6 van Steijn ME, Scheepstra KWF, Yasar G, *et al.* Occupational well-being in pediatricians—a survey about work-related posttraumatic stress, depression, and anxiety. *Eur J Pediatr* Published Online First: 2019. doi:10.1007/s00431-019-03334-7

7 Kerkman T, Dijksman LM, Baas MAM, *et al.* Traumatic Experiences and the Midwifery Profession: A Cross-Sectional Study Among Dutch Midwives. *J Midwifery Women's Heal* Published Online First: 2019. doi:10.1111/jmwh.12946

8 Baas MAM, Scheepstra KWF, Stramrood CAI, *et al.* Work-related adverse events leaving their mark: A cross-sectional study among Dutch gynecologists. *BMC Psychiatry* 2018;**18**. doi:10.1186/s12888-018-1659-1

9 Frank JR, Snell L, Sherbino J E. CanMEDs 2015 Physician Competency Framework. CanMEDS 2015 Physician Competency Fram. Ottawa R. Coll. Physicians Surg. Canada. 2015;:1–30.

10 Hu YY, Fix ML, Hevelone ND, *et al.* Physicians' needs in coping with emotional stressors: The case for peer support. *Arch Surg* Published Online First: 2012. doi:10.1001/archsurg.2011.312

11 American Psychiatric Association. Diagnostic and Statistical Manual of Mental Disorders: DSM-5. *Am Psychiatr Assoc* 2013;:991. doi:10.1176/appi.books.9780890425596.744053

12 Alonso J, Angermeyer MC, Bernert S, *et al.* Prevalence of mental disorders in Europe: results from the European Study of the Epidemiology of Mental Disorders (ESEMeD) project. *Acta Psychiatr Scand Suppl* 2004;**109**:21–7. doi:10.1111/j.1600-0047.2004.00327.x

13 De Graaf R, Ten Have M, Van Gool C, *et al.* Prevalence of mental disorders and trends from 1996 to 2009. Results from the Netherlands Mental Health Survey and Incidence Study-2. Soc. Psychiatry Psychiatr. Epidemiol. 2012;**47**:203–13. doi:10.1007/s00127-010-0334-8

14 Mata DA, Ramos MA, Bansal N, *et al.* Prevalence of Depression and Depressive Symptoms Among Resident Physicians: A Systematic Review and Meta-analysis. *JAMA* 2015;**314**:2373–83. doi:10.1001/jama.2015.15845

For peer review only

For peer review only - http://bmjopen.bmj.com/site/about/guidelines.xhtml

15 Ruitenburg MM, Frings-Dresen MH, Sluiter JK. The prevalence of common mental disorders among hospital physicians and their association with self-reported work ability: a cross-sectional study. *BMC Health Serv Res* 2012;**12**:292. doi:10.1186/1472-6963-12-292

16 Wurm W, Vogel K, Holl A, *et al.* Depression-burnout overlap in physicians. *PLoS One* Published Online First: 2016. doi:10.1371/journal.pone.0149913

17 Hsu K, Marshall V. Prevalence of depression and distress in a large sample of Canadian residents, interns, and fellows. *Am J Psychiatry* Published Online First: 1987. doi:10.1176/ajp.144.12.1561

18 Tomioka K, Morita N, Saeki K, *et al.* Working hours, occupational stress and depression among physicians. *Occup Med (Chic Ill)* Published Online First: 2011. doi:10.1093/occmed/kqr004

19 O T, A A, D T, *et al.* Physician Suicide: A Silent Epidemic. In: *In: APA annual meeting, Poster no 227, New York City*. 2018. 123–4.

20 Kessler RC, Aguilar-Gaxiola S, Alonso J, *et al.* Trauma and PTSD in the WHO World Mental Health Surveys. Eur. J. Psychotraumatol. 2017. doi:10.1080/20008198.2017.1353383

21 Joseph B, Pandit V, Hadeed G, *et al.* Unveiling posttraumatic stress disorder in trauma surgeons. *J Trauma Acute Care Surg* 2014;**77**:148–54. doi:10.1097/TA.0000000000000271

22 Shi L, Wang L, Jia X, *et al.* Prevalence and correlates of symptoms of post-traumatic stress disorder among Chinese healthcare workers exposed to physical violence: a cross-sectional study. *BMJ Open* 2017;**7**:e016810. doi:10.1136/bmjopen-2017-016810

23 Chan AOM, Huak CY. Influence of work environment on emotional health in a health care setting. *Occup Med (Lond)* 2004;**54**:207–12. doi:10.1093/occmed/kqh062

24 Mills LD, Mills TJ. Symptoms of post-traumatic stress disorder among emergency medicine residents. J. Emerg. Med. 2005. doi:10.1016/j.jemermed.2004.05.009

25 Wahlberg, Andreen Sachs M, Johannesson K, *et al.* Post-traumatic stress symptoms in Swedish obstetricians and midwives after severe obstetric events: a cross-sectional retrospective survey. *BJOG An Int J Obstet Gynaecol* Published Online First: 2017. doi:10.1111/1471-0528.14259

26 Spinhoven P, Ormel J, Sloekers PP, *et al.* A validation study of the Hospital Anxiety and Depression Scale (HADS) in different groups of Dutch subjects. *Psychol Med* 1997;**27**:363–70. doi:10.1017/S0033291796004382

27 Bjelland I, Dahl AA, Haug TT, *et al.* The validity of the Hospital Anxiety and Depression Scale. An updated literature review. *J Psychosom Res* 2002.

28 Dekkers AMM, Olff M, Maring GWB. Identifying persons at risk for PTSD after trauma with TSQ in the Netherlands. *Community Ment Health J* 2010;**46**:20–5. doi:10.1007/s10597-009-9195-6

29 Kessler RC, Chiu WT, Demler O, *et al.* Prevalence, severity, and comorbidity of 12-month DSM-IV disorders in the National Comorbidity Survey Replication. *Arch Gen Psychiatry* 2005;**62**:617–27. doi:10.1001/archpsyc.62.6.617

30 Substance Abuse and Mental Health Services Administration, Center for Behavioural Health Statistics and Quality. Results from the 2015 National Survey on Drug Use and Health: Detailed Tables,. 2015.

31 Hope V, Henderson M. Medical student depression, anxiety and distress outside North

For peer review only - http://bmjopen.bmj.com/site/about/guidelines.xhtml

America: a systematic review. *Med Educ* 2014;**48**:963–79. doi:10.1111/medu.12512

32 Wall TD, Bolden RI, Borrill CS, *et al.* Minor psychiatric disorder in NHS trust staff: Occupational and gender differences. *Br J Psychiatry* 1997;**171**:519–23. doi:10.1192/bjp.171.6.519

33 Capaciteitsorgaan. Capaciteitsplan 2016 (in Dutch). Utrecht: 2016. www.capaciteitsorgaan.nl accessed 2-7-2018

34 Seys D, Wu AW, Gerven E Van, *et al.* Health Care Professionals as Second Victims after Adverse Events. *Eval Health Prof* 2013;**36**:135–62. doi:10.1177/0163278712458918

35 De Vries GJ, Olff M. The lifetime prevalence of traumatic events and posttraumatic stress disorder in the Netherlands. *J Trauma Stress* Published Online First: 2009. doi:10.1002/jts.20429

36 Carlier I V, Lamberts RD, Gersons BP. Risk factors for posttraumatic stress symptomatology in police officers: a prospective analysis. *J Nerv Ment Dis* Published Online First: 1997. doi:10.1097/00005053-199708000-00004

37 Dyrbye LN, West CP, Sinsky CA, *et al.* Medical Licensure Questions and Physician Reluctance to Seek Care for Mental Health Conditions. *Mayo Clin Proc* Published Online First: 2017. doi:10.1016/j.mayocp.2017.06.020

38 Baverstock A, Finlay F. Specialist registrars' emotional responses to a patient's death. *Arch Dis Child* Published Online First: 2006. doi:10.1136/adc.2005.076760

39 Olff M, Langeland W, Draijer N, *et al.* Gender differences in posttraumatic stress disorder. [Review] [287 refs]. *Psychol Bull* Published Online First: 2007. doi:10.1037/0033-2909.133.2.183

40 Schnider KR, Elhai JD, Gray MJ. Coping style use predicts posttraumatic stress and complicated grief symptom severity among college students reporting a traumatic loss. *J Couns Psychol* 2007;**54**:344–50. doi:10.1037/0022-0167.54.3.344

41 Kerai SM, Khan UR, Islam M, *et al.* Post-traumatic stress disorder and its predictors in emergency medical service personnel: a cross-sectional study from Karachi, Pakistan. *BMC Emerg Med* 2017;**17**:26. doi:10.1016/j.brat.2003.07.010

42 Brosschot JF. Ever at the ready for events that never happen. *Eur J Psychotraumatol* Published Online First: 2017. doi:10.1080/20008198.2017.1309934

43 Olff M. Bonding after trauma: On the role of social support and the oxytocin system in traumatic stress. *Eur J Psychotraumatol* Published Online First: 2012. doi:10.3402/ejpt.v3i0.18597

44 Fluid Surveys University. Response Rate Statistics for Online Surveys - What Numbers Should You be Aiming For? 2014.

45 Shanafelt TD, Boone S, Tan L, *et al.* Burnout and satisfaction with work-life balance among US physicians relative to the general US population. *Arch Intern Med* Published Online First: 2012. doi:10.1001/archinternmed.2012.3199

For peer review only - http://bmjopen.bmj.com/site/about/guidelines.xhtml

For peer review only

For peer review only - http://bmjopen.bmj.com/site/about/guidelines.xhtml

Supplementary files

1. Questionnaire
2. Supplementary table: logistic regression work experience vs. HADS-D and HADS-A

For peer review only

For peer review only - http://bmjopen.bmj.com/site/about/guidelines.xhtml

Supplementary file 1: Questionnaires

Paediatricians

1 What is your gender?
1. Male
2. Female

2 What is your age?
1. 20-29 years
2. 30-39 years
3. 40-49 years
4. 50-59 years
5. 60-69 years
6. ≥ 70 years

3 Are you a member of the Dutch Society of Paediatricians (NvK)?
1. Yes
2. No

4 What is your current position?
1. Resident
2. Attending physician
3. Retired
4. Non-practicing/management

5 How many years have you been working in the paediatrics field as a physician? (Including as intern)
1. ≤ 5 years
2. 6-10 years
3. 11-15 years
4. 16-20 years
5. 21-25 years
6. 26-30 years
7. > 30 years

6 At the moment, are you working in a general hospital or in an academic referral centre?
1. General hospital
2. Academic referral centre
3. Both
4. I do not work anymore, but I used to work in a general hospital
5. I do not work anymore, but I used to work in an academic referral centre

7 At which department do you work the most? (If you are not practicing anymore, fill in where you used to work)
1. Neonatology
2. Emergency room
3. General paediatrician
4. NICU
5. Child intensive care
6. Policlinic
7. Children's oncology
8. Other…

8 What do you consider to be an adverse event on the work-floor? (More than one answer possible)
1. Not applicable

For peer review only

2. Bad news conversation/interview
3. (Critically) ill neonate
4. (Critically) ill children
5. When a patient dies
6. When you miss a diagnosis
7. When you have to refuse a patient
8. When you are in doubt whether you are making the right decision
9. When an inexperienced colleague is on call
10. When you decide on an abstaining course of treatment
11. Treatment of neonates
12. When you know a patient will remain severely handicapped
13. Handling the emotions of the parents
14. (Suspicion of) child abuse
15. Other …

9 How do you cope with adverse events on the work-floor? (More than one answer possible)

1. Not applicable / never experienced
2. Seeking professional help
3. Use new medication
4. Drink (more) alcohol
5. Smoke (more) cigarettes
6. Use (more) drugs
7. Work out (more)
8. Going home as soon as possible
9. Call in sick
10. Hide your emotions
11. Find a distraction
12. Praying or other religious activities
13. Talking to friends and/or family
14. Informally discussing the matter with peers/colleagues
15. Quitting
16. Work less
17. Not working any evening/night shifts
18. Other …

10 The current support organized by your institution after an adverse event is good:

1. Strongly disagree
2. Disagree
3. Agree
4. Strongly agree

11 There is plenty of room to informally discuss adverse events in the department/partnership:

1. Strongly disagree
2. Disagree
3. Agree
4. Strongly agree

12 There is plenty of room to informally discuss adverse events in the hospital:

1. Strongly disagree
2. Disagree
3. Agree
4. Strongly agree

13 There should be a culture change regarding support after an adverse event:

1. Strongly disagree

For peer review only

2. Disagree
3. Agree
4. Strongly agree

14 There is a protocol at your institution regarding support after an adverse event:
1. Yes
2. Yes, but nobody uses it
3. No, but there is one currently being created
4. No
5. I do not know

15 Support after an adverse event consisted of: (more than one answer possible)
1. I have never experienced an adverse event
2. There was none
3. Professionally organised peer-support
4. Self-initiated peer-support with direct colleagues (own department)
5. Self-initiated peer-support with indirect colleagues (different department)
6. Conversation(s) with a psychologist or coach
7. Evaluation with the present team
8. Help from the medical officer after a sick-leave
9. Other …

16 Your preferred support after an adverse event would be: (more than one answer possible)
1. Not applicable
2. Support is unnecessary
3. Professionally organised peer-support
4. Evaluation with the present team
5. Peer-support from direct colleagues (own department)
6. Peer-support from indirect colleagues (different department)
7. Mindfulness
8. Other …

17 It should be mandatory for the hospital to organise support after an adverse event:
1. Yes
2. No

18 You have learned to cope with adverse events through/during:
1. Med-school (without clerkships)
2. Clerkships
3. Internships
4. Residency
5. Attending physician
6. Other study
7. Psychological help
8. Specific course/training
9. Peer-review
10. Mindfulness
11. Never learnt
12. Other…

19 In the course of your career, you have become more defensive:
1. Strongly disagree
2. Disagree
3. Agree
4. Strongly agree

For peer review only

20 Have you changed your working-conditions (e.g. less shifts, more diagnostic tests) after experiencing a patient-related adverse event?

1. Yes
2. No

21 What did you change? (More than one answer possible)

1. Work less
2. Less evening/night-shifts
3. No evening/night-shifts anymore
4. Calling a colleague sooner
5. Quit my job
6. More diagnostic tests
7. Start treatment sooner
8. Other…

22 Sometimes people experience traumatic events, such as live-threatening situations as a cause of a natural disaster, high-impact trauma or fire; being attacked or raped; witness a murder, death or hear find out someone close to them experienced something terrible. As a physician, one can experience such events in patient-care: critical illness or death of a patients, severe injury, as well as violent behaviour from a patient or their family.
Have you ever, during your work AS A PHYSICIAN experienced such (adverse) events/incidents?

1. Yes
2. No

23 Can you briefly describe the adverse event(s)?

1. Yes, please describe it…
2. No

24 Did (some part of) this/these adverse event(s) take place more than 4 weeks ago?

1. Yes
2. No

Followed by the TSQ questionnaire.

35 It might be that you did not experience the reactions and emotions as described on the previous pages, but that you recognize them from a past period in your life. For example, having disturbing thoughts or dreams, reliving the event, getting upset. Do you recognize these reactions?

1. Yes
2. No

36 Which of these reactions do you recognize? (More than one answer possible)

1. Disturbing thoughts
2. Disturbing dreams
3. Reliving experiences
4. Getting upset
5. Physical responses
6. Insomnia
7. Irritability
8. Trouble focussing
9. Hyper arousal
10. Tensed

37 How long did these symptoms last?

1. < 4 weeks

For peer review only

2. > 4 weeks
3. > 6 months
4. > 1 year

38 Have you ever been diagnosed with PTSD (posttraumatic stress-disorder)?
1. Yes
2. No

Followed by the HADS questionnaire.

53 Have you ever seriously considered quitting your job as a paediatrician? (e.g. by looking for a different job, talking to human resources about ending your contract?
1. Yes, absolutely
2. Yes, sometimes
3. No, never

54 What was the reason to consider quitting? (More than one answer possible)
1. Bad collaboration (working together) with a co-worker
2. Not challenging enough within the field
3. A lot of complications
4. Afraid to make mistakes
5. Too much responsibility
6. Traumatic experience on the work-floor
7. Complaint(s) from patients
8. One-sided
9. A lot of stress
10. A high workload
11. Disbalance between work and private life
12. Disagreements with work providers
13. Different interests
14. New challenge
15. Too much administration
16. Too much bureaucracy
17. Patient-violence
18. Too many rules
19. Insufficient guidance from supervisors
20. Problems with the partnership
21. Private reasons
22. Disutility
23. Other…

55 Did you ever faced a complaint at the disciplinary board?
1. Yes
2. No

Gynaecologists

1 Gender
1. Female
2. Male

2 Age
1. 25-34 years
2. 35-44 years

For peer review only

3. 45-54 years
4. 55-64 years
5. 65 years and older

3 Current position
1. Resident
2. Attending / fellow
3. Retired gynaecologist
4. Non-practicing/management

4 How many years have you been working as a gynaecologist? (Including working as a resident)

5 I considered quitting my job as a gynaecologist:
1. Strongly disagree
2. Disagree
3. Agree
4. Strongly agree

6 In case you considered quitting, what was the most important reason?
1. Most important reason: …
2. Never considered quitting

7 Did you ever face a complaint at the disciplinary board?
1. Yes
2. No

8 What do you consider to be an adverse event on the work-floor? (More than one answer possible)
1. Not applicable
2. Bad news conversations/interview
3. The critical moments when a patient's life is at risk
4. When a patient dies
5. When you miss a diagnosis
6. When you feel like you cannot help the patients
7. When you are in doubt whether you are making the right decision
8. Other …

9 After experiencing adverse events on the work-floor, I do the following: (More than one answer possible)
1. Not applicable
2. Seeking professional help
3. Going home as soon as possible
4. Drink more alcohol, use more drugs and smoke more cigarettes than I normally would do
5. Use new medication I barely or normally not use
6. Trying not to think about it
7. Find a distraction
8. Praying or other religious activities
9. Formal case review
10. Work out or doing my hobby
11. Talking to friends and/or family
12. Informally discussing the matter with peers/colleagues
13. I developed symptoms of a burn-out (e.g. emotional exhaustion, depersonalisation, lower self-esteem)
14. Call in sick
15. Quit my job
16. Other …

For peer review only

10 The current support organized by your institution after an adverse event is good:
1. Strongly disagree
2. Disagree
3. Agree
4. Strongly agree

11 There is plenty of room to informally discuss adverse events in the department/partnership:
1. Strongly disagree
2. Disagree
3. Agree
4. Strongly agree

12 You have learned to cope with adverse events through/during:
1. Clerkships
2. Residency
3. Specific course/training
4. Intervision
5. Other …
6. Never really learned it

13 After experiencing an adverse event I changed my working conditions (e.g. no more shifts, no more vaginal breech births, not performing any surgeries alone, coming to the hospital sooner instead of supervision over the telephone)
1. Strongly disagree
2. Disagree
3. Agree
4. Strongly agree

14 In the course of your career, you have become more defensive:
1. Strongly disagree
2. Disagree
3. Agree
4. Strongly agree

15 Support after an adverse event consisted of… (fill in the box)

16 There is a protocol at your institution regarding support after an adverse event:
1. No
2. I do not know
3. Yes, consisting of…

17 Your preferred support after an adverse event would be: (more than one answer possible)
1. Evaluation with direct colleagues
2. Intervision with indirect colleague's (physicians with a different specialty)
3. Talking to a psychologist or coach
4. A buddy
5. Other …

18 When you lay awake at night, you are not made for this job:
1. Strongly disagree
2. Disagree
3. Agree
4. Strongly agree

For peer review only

19 There should be a culture change regarding support after an adverse event:
1. Strongly disagree
2. Disagree
3. Agree
4. Strongly agree

20 Sometimes people experience traumatic events, such as live-threatening situations as a cause of a natural disaster, high-impact trauma or fire; being attacked or raped; witness a murder, death or hear find out someone close to them experienced something terrible. As a physician, one can experience such events in patient-care: critical illness or death of a patients, severe injury, as well as violent behaviour from a patient or their family.
1) Have you ever, during your work AS A PHYSICIAN experienced such (adverse) events/incidents?
2) After at least one of those adverse events, did you experience intense fear, helplessness or abomination?
3) Did this/these event(s) happened at least 4 weeks ago?
1. I answered yes to all of these questions
2. No, I did not answer yes to all these questions

Followed by the TSQ

31 Can you briefly describe the adverse event(s)?

32 It might be that you did not experience the reactions and emotions as described on the previous pages, but that you recognize them from a past period in your life. For example, having disturbing thoughts or dreams, reliving the event, getting upset. Do you recognize these reactions?
1. Yes
2. No

Followed by the HADS.

Othopaedic surgeons

1 What is your gender?
1. Male
2. Female

2 What is your age?
1. 20-29 years
2. 30-39 years
3. 40-49 years
4. 50-59 years
5. 60-69 years
6. ≥ 70 years

3 Are you a member of the Dutch Orthopaedic Society (NOV)?
1. Yes
2. No

4 What is your current position?
1. Resident
2. Attending physician
3. Retired
4. Non-practicing/management

For peer review only

5 How many years have you been working in the orthopaedic field as a physician? (Including as intern)

1. ≤ 5 years
2. 6-10 years
3. 11-15 years
4. 16-20 years
5. 21-25 years
6. 26-30 years
7. > 30 years

6 What do you consider to be an adverse event on the work-floor? (More than one answer possible)

1. Not applicable
2. Bad news conversation/interview
3. (Critically) ill patients
4. When a patient dies
5. When you miss a diagnosis
6. When you have to refuse a patient
7. When you are in doubt whether you are making the right decision
8. When an inexperienced colleague is on call
9. When you decide on an abstaining course of treatment
10. Treatment of young patients
11. When you know a patient will remain severely handicapped
12. Other …

7 How do you cope with adverse events on the work-floor? (More than one answer possible)

1. Not applicable / never experienced
2. Seeking professional help
3. Use new medication
4. Drink (more) alcohol
5. Smoke (more) cigarettes
6. Use (more) drugs
7. Work out (more)
8. Going home as soon as possible
9. Call in sick
10. Hide your emotions
11. Find a distraction
12. Praying or other religious activities
13. Talking to friends and/or family
14. Informally discussing the matter with peers/colleagues
15. Quitting
16. Work less
17. Other …

8 The current support organized by your institution after an adverse event is good:

1. Strongly disagree
2. Disagree
3. Agree
4. Strongly agree

9 There is plenty of room to informally discuss adverse events in the department/partnership:

1. Strongly disagree
2. Disagree
3. Agree
4. Strongly agree

For peer review only

10 There is plenty of room to informally discuss adverse events in the hospital:
1. Strongly disagree
2. Disagree
3. Agree
4. Strongly agree

11 There should be a culture change regarding support after an adverse event:
1. Strongly disagree
2. Disagree
3. Agree
4. Strongly agree

12 There is a protocol at your institution regarding support after an adverse event:
1. Yes
2. Yes, but nobody uses it
3. No, but there is one currently being created
4. No
5. I do not know

13 Support after an adverse event consisted of: (more than one answer possible)
1. I have never experienced an adverse event
2. There was none
3. Professionally organised peer-support
4. Self-initiated peer-support with direct colleagues (own department)
5. Self-initiated peer-support with indirect colleagues (different department)
6. Conversation(s) with a psychologist or coach
7. Evaluation with the present team
8. Help from the medical officer after a sick-leave
9. Other …

14 Your preferred support after an adverse event would be: (more than one answer possible)
1. Not applicable
2. Support is unnecessary
3. Professionally organised peer-support
4. Evaluation with the present team
5. Peer-support from direct colleagues (own department)
6. Peer-support from indirect colleagues (different department)
7. Mindfulness
8. Other …

15 It should be mandatory for the hospital to organise support after an adverse event:
1. Yes
2. No

16 You have learned to cope with adverse events through/during:
1. Med-school (without clerkships)
2. Clerkships
3. Internships
4. Residency
5. Attending physician
6. Psychological help
7. Specific course/training
8. Peer-review
9. Mindfulness
10. Never learnt

For peer review only

17 In the course of your career, you have become more defensive:
1. Strongly disagree
2. Disagree
3. Agree
4. Strongly agree

18 Have you changed your working-conditions (e.g. less shifts, more diagnostic tests) after experiencing a patient-related adverse event?
1. Yes
2. No

19 What did you change? (More than one answer possible)
1. Work less
2. Less evening/night-shifts
3. No evening/night-shifts anymore
4. Calling a colleague sooner
5. Quit
6. Start treatment sooner
7. Other…

20 Have you ever been diagnosed with PTSD (posttraumatic stress-disorder)?
1. Yes
2. No

21 Sometimes people experience traumatic events, such as live-threatening situations as a cause of a natural disaster, high-impact trauma or fire; being attacked or raped; witness a murder, death or hear find out someone close to them experienced something terrible. As a physician, one can experience such events in patient-care: critical illness or death of a patients, severe injury, as well as violent behaviour from a patient or their family.
Have you ever, during your work AS A PHYSICIAN experienced such (adverse) events/incidents?
1. Yes
2. No

22 Did this/these take place less than 4 weeks ago?
1. Yes
2. No

23 Can you describe the event/incident?
1. Yes
2. No

Followed by the TSQ and HADS questionnaire.

51 Have you ever seriously considered quitting your job as an orthopaedic surgeon? (e.g. by looking for a different job, talking to human resources about ending your contract?
1. Yes
2. No

52 What was the reason to consider quitting? (More than one answer possible)
1. Bad collaboration (working together) with a co-worker
2. Not challenging enough within the field
3. A lot of complication
4. Afraid to make mistakes
5. Too much responsibility

For peer review only

6. Traumatic experience on the work-floor
7. Complaint(s) from patients
8. One-sided
9. A lot of stress
10. A high workload
11. Disbalance between work and private life
12. Disagreements with work providers
13. Different interests
14. New challenge
15. Too much administration
16. Too much bureaucracy
17. Patient-violence
18. Too many rules
19. Insufficient guidance from supervisors
20. Problems with the partnership
21. Private reasons
22. Disutility
23. Other…

53 Did you ever face a complaint at the disciplinary board?

1. Yes
2. No

For peer review only

Supplementary file 2: logistic regression model and odds ratios 'work experience – HADS-D and HADS-A'

HADS-D

	Estimate	Standard Error	Odds Ratio	z	p
(Intercept)	-3.314	0.416	0.036	-7.974	< .001
Work experience (5-10 years)	0.302	0.510	1.352	0.592	0.554
Work experience (10-15 years)	0.849	0.486	2.337	1.746	0.081
Work experience (15-20 years)	1.023	0.484	2.781	2.115	0.034
Work experience (20-25 years)	0.979	0.496	2.661	1.974	0.048
Work experience (25-30 years)	1.081	0.497	2.946	2.176	0.030
Work experience (≥ 30 years)	-0.385	0.615	0.681	-0.626	0.531

Model summary

Model	Deviance	AIC	BIC	df	X^2	p	McFadden R^2	Nagelkerke R^2	Tjur R^2
H_0	653.6	655.648	660.872	1371					
H_1	635.5	649.472	686.040	1365	18.176	0.006	0.028	0.035	0.033

HADS-A

	Estimate	Standard Error	Odds Ratio	z	p
(Intercept)	-1.508	0.198	0.221	-7.595	< .001
Work experience (5-10 years)	-0.179	0.263	0.836	-0.680	0.496
Work experience (10-15 years)	-0.361	0.281	0.697	-1.284	0.199
Work experience (15-20 years)	-0.164	0.279	0.848	-0.589	0.556
Work experience (20-25 years)	-0.347	0.299	0.707	-1.160	0.246
Work experience (25-30 years)	-0.461	0.315	0.631	-1.461	0.144
Work experience (≥ 30 years)	-1.116	0.341	0.328	-3.276	0.001

Model summary

Model	Deviance	AIC	BIC	df	X^2	p	McFadden R^2	Nagelkerke R^2	Tjur R^2
H_0	1093	1094.619	1099.843	1371					
H_1	1078	1092.140	1128.708	1365	14.479	0.025	0.013	0.019	0.000

For peer review only

STROBE 2007 (v4) Statement—Checklist of items that should be included in reports of *cross-sectional studies*

**Section/Topic**	**Item #**	**Recommendation**	**Reported on page #**
**Title and abstract**	1	(*a*) Indicate the study's design with a commonly used term in the title or the abstract	1 - 2
		(*b*) Provide in the abstract an informative and balanced summary of what was done and what was found	1 – 2
**Introduction**			
Background/rationale	2	Explain the scientific background and rationale for the investigation being reported	3 – 4
Objectives	3	State specific objectives, including any prespecified hypotheses	4
**Methods**			
Study design	4	Present key elements of study design early in the paper	4 - 5
Setting	5	Describe the setting, locations, and relevant dates, including periods of recruitment, exposure, follow-up, and data collection	4
Participants	6	(*a*) Give the eligibility criteria, and the sources and methods of selection of participants	4
Variables	7	Clearly define all outcomes, exposures, predictors, potential confounders, and effect modifiers. Give diagnostic criteria, if applicable	5
Data sources/ measurement	8*	For each variable of interest, give sources of data and details of methods of assessment (measurement). Describe comparability of assessment methods if there is more than one group	4
Bias	9	Describe any efforts to address potential sources of bias	*5*
Study size	10	Explain how the study size was arrived at	-
Quantitative variables	11	Explain how quantitative variables were handled in the analyses. If applicable, describe which groupings were chosen and why	5
Statistical methods	12	(*a*) Describe all statistical methods, including those used to control for confounding	5-6
		(*b*) Describe any methods used to examine subgroups and interactions	5-6
		(*c*) Explain how missing data were addressed	5-6
		(*d*) If applicable, describe analytical methods taking account of sampling strategy	-
		(*e*) Describe any sensitivity analyses	-
**Results**			

For peer review only

Participants	13*	(a) Report numbers of individuals at each stage of study—eg numbers potentially eligible, examined for eligibility, confirmed eligible, included in the study, completing follow-up, and analysed	7
		(b) Give reasons for non-participation at each stage	7
		(c) Consider use of a flow diagram	-
Descriptive data	14*	(a) Give characteristics of study participants (eg demographic, clinical, social) and information on exposures and potential confounders	7
		(b) Indicate number of participants with missing data for each variable of interest	7
Outcome data	15*	Report numbers of outcome events or summary measures	8-10
Main results	16	(*a*) Give unadjusted estimates and, if applicable, confounder-adjusted estimates and their precision (eg, 95% confidence interval). Make clear which confounders were adjusted for and why they were included	8-10
		(*b*) Report category boundaries when continuous variables were categorized	-
		(*c*) If relevant, consider translating estimates of relative risk into absolute risk for a meaningful time period	-
Other analyses	17	Report other analyses done—eg analyses of subgroups and interactions, and sensitivity analyses	8-10
**Discussion**			
Key results	18	Summarise key results with reference to study objectives	10-12
Limitations	19	Discuss limitations of the study, taking into account sources of potential bias or imprecision. Discuss both direction and magnitude of any potential bias	13
Interpretation	20	Give a cautious overall interpretation of results considering objectives, limitations, multiplicity of analyses, results from similar studies, and other relevant evidence	14
Generalisability	21	Discuss the generalisability (external validity) of the study results	13
**Other information**			
Funding	22	Give the source of funding and the role of the funders for the present study and, if applicable, for the original study on which the present article is based	17

*Give information separately for cases and controls in case-control studies and, if applicable, for exposed and unexposed groups in cohort and cross-sectional studies.

Note: An Explanation and Elaboration article discusses each checklist item and gives methodological background and published examples of transparent reporting. The STROBE checklist is best used in conjunction with this article (freely available on the Web sites of PLoS Medicine at http://www.plosmedicine.org/, Annals of Internal Medicine at http://www.annals.org/, and Epidemiology at http://www.epidem.com/). Information on the STROBE Initiative is available at www.strobe-statement.org.

For peer review only

BMJ Open

Potential traumatic events in the workplace and depression, anxiety and posttraumatic stress: a cross-sectional study among Dutch gynaecologists, paediatricians and orthopaedic surgeons.

Journal:	*BMJ Open*
Manuscript ID	bmjopen-2019-033816.R2
Article Type:	Original research
Date Submitted by the Author:	27-Mar-2020

Complete List of Authors:	Scheepstra, Karel; Amsterdam UMC - Locatie AMC, Psychiatry<br>Pauw, Hannah; Amsterdam UMC - Locatie AMC, Faculty of Medicine<br>van Steijn, Minouk; Onze Lieve Vrouw Hosp, Obstetrics and Gynaecology; Amsterdam UMC - Locatie AMC, Obstetrics and Gynaecology<br>Stramrood, Claire; OLVG Locatie Oost, Obstetrics and Gynaecology<br>Olff, Miranda; Amsterdam UMC - Locatie AMC, Psychiatry; Arq Psychotrauma Research<br>van Pampus, Maria G.; Onze Lieve Vrouw Hosp, Obstetrics and Gynaecology

**Primary Subject Heading**:	Mental health
Secondary Subject Heading:	Occupational and environmental medicine, Health services research
Keywords:	OCCUPATIONAL & INDUSTRIAL MEDICINE, PSYCHIATRY, Depression & mood disorders < PSYCHIATRY, Anxiety disorders < PSYCHIATRY, Adverse events < THERAPEUTICS, Protocols & guidelines < HEALTH SERVICES ADMINISTRATION & MANAGEMENT

SCHOLARONE™
Manuscripts

I, the Submitting Author has the right to grant and does grant on behalf of all authors of the Work (as defined in the below author licence), an exclusive licence and/or a non-exclusive licence for contributions from authors who are: i) UK Crown employees; ii) where BMJ has agreed a CC-BY licence shall apply, and/or iii) in accordance with the terms applicable for US Federal Government officers or employees acting as part of their official duties; on a worldwide, perpetual, irrevocable, royalty-free basis to BMJ Publishing Group Ltd ("BMJ") its licensees and where the relevant Journal is co-owned by BMJ to the co-owners of the Journal, to publish the Work in this journal and any other BMJ products and to exploit all rights, as set out in our licence.

The Submitting Author accepts and understands that any supply made under these terms is made by BMJ to the Submitting Author unless you are acting as an employee on behalf of your employer or a postgraduate student of an affiliated institution which is paying any applicable article publishing charge ("APC") for Open Access articles. Where the Submitting Author wishes to make the Work available on an Open Access basis (and intends to pay the relevant APC), the terms of reuse of such Open Access shall be governed by a Creative Commons licence – details of these licences and which Creative Commons licence will apply to this Work are set out in our licence referred to above.

Other than as permitted in any relevant BMJ Author's Self Archiving Policies, I confirm this Work has not been accepted for publication elsewhere, is not being considered for publication elsewhere and does not duplicate material already published. I confirm all authors consent to publication of this Work and authorise the granting of this licence.

For peer review only

Potential traumatic events in the workplace and depression, anxiety and posttraumatic stress: a cross-sectional study among Dutch gynaecologists, paediatricians and orthopaedic surgeons.

For peer review only

Authors

Karel W. F. Scheepstra*, MD[1]
Hannah S. Pauw, BSc[2]
Minouk E. van Steijn, MD[3,4]
Claire A.I. Stramrood, MD PhD[4]
Miranda Olff, PhD[1,5]
Maria G. van Pampus, MD PhD[4]

1. Amsterdam UMC, University of Amsterdam, Department of Psychiatry, Meibergdreef 9, 1105 AZ, Amsterdam, the Netherlands.
2. Amsterdam UMC, University of Amsterdam, Faculty of Medicine, Meibergdreef 9, 1105 AZ, Amsterdam, the Netherlands.
3. Amsterdam UMC, University of Amsterdam, Department of Obstetrics and Gynaecology, Meibergdreef 9, 1105 AZ, Amsterdam, the Netherlands.
4. OLVG, Department of Obstetrics and Gynaecology, Oosterpark 9, 1090 HM, Amsterdam, the Netherlands
5. Arq Psychotrauma Expert Group, Nienoord 5, 1112 XE, Diemen, the Netherlands

***Corresponding author:**
K.W.F. Scheepstra
Department of Psychiatry
Meibergdreef 9
1105 AZ Amsterdam
The Netherlands
k.w.scheepstra@amsterdamumc.nl
+31 (0) 20 8913500

ABSTRACT

Objective: To compare the prevalence of work-related potential traumatic events, support protocols and mental health symptoms across Dutch gynaecologists, orthopaedic surgeons and paediatricians.

Design: Cross-sectional study, supplementary analysis of combined data.

Setting: Nationwide survey between 2014 and 2017.

Participants: An online questionnaire was sent to all Dutch gynaecologists, orthopaedic surgeons and paediatricians, including resident physicians (4959 physicians). 1374 questionnaires were eligible for analysis, corresponding with a response rate of 27.7%.

Outcome measures: Primary outcome measures were the prevalence of work-related potential traumatic events (PTEs), depression, anxiety, psychological distress and traumatic stress, measured with validated screening instruments (Hospital Anxiety and Depression Scale, HADS; Trauma Screening Questionnaire, TSQ). Secondary outcomes were the association of mental health and defensive practice to traumatic events and support protocols.

Results: Of the respondents, 20.8% experienced a work-related potential traumatic event at least four weeks ago. Prevalence rates indicative of depression, anxiety or posttraumatic stress disorder were 6.4%, 13.6% and 1.5% respectively. Depression (9.2% vs. 5.2%, p=0.019), anxiety (18.2% vs. 8.2%, p<0.001) and psychological distress (22.8% vs. 12.5%, p<0.001), were significantly more prevalent in female compared to male attendings. The absence of a support protocol was significantly associated with more probable posttraumatic stress disorder (p=0.022).

Conclusion: Physicians are frequently exposed to potential traumatic events with high emotional impact over the course of their career. Lacking a support protocol after adverse events was associated with more posttraumatic stress. Adverse events were associated with considering to quit medical practice and a more defensive practice. More awareness must be created for the mental health of physicians as well as for the implementation of a well-organised support system after potential traumatic events.

Word count: 263

Keywords: Occupational & Industrial Medicine, Psychiatry, Depression & mood disorders, Anxiety disorders, Adverse Events, Protocols & guidelines

ARTICLE SUMMARY

Strengths and limitations of this study

- This is one of the few studies done on PTSD in physicians, in particular with regard to mental health in relation to social and occupational functioning and clinical practice related consequences.
- This study incorporated the A and E criterion in our probable PTSD diagnosis, therefore the prevalence is more likely to reflect the real prevalence of PTSD.
- One limitation is that the population of this study is limited to three specialties and one country in Europe and thus it is difficult to translate these results to physicians worldwide.

For peer review only - http://bmjopen.bmj.com/site/about/guidelines.xhtml

INTRODUCTION

Physicians are frequently exposed to medical adverse events, such as life-threatening situations, illnesses, grief, death, and patient violence and aggression. Dealing with such events makes the medical profession one with occupational health hazards, comparable with fire fighters, rescue workers, military and police personnel.[1–3] The support for physicians is, however, underdeveloped in most areas of the medical profession compared to other professions.[4,5] It is generally assumed that health care providers have adequate coping mechanisms to deal with such work stressors. However, adequate coping mechanisms do not warrant successful coping with severe potential traumatic events. Our previous work showed that most gynaecologists, paediatricians and midwives experience support and protocols after work-related potential traumatic events as insufficient. Dutch paediatricians reported ´missing a diagnosis´ as having the most emotional impact and ´aggressive behaviour from parents´ as the most common adverse event. [6] Midwives working in primary care reported higher levels of anxiety compared with their colleagues working in secondary or tertiary care.[7] Gynaecologists reported that development of coping strategies with work-related stressors are neither taught during graduate nor in specialist training.[8] Physician health is one of the competencies described in the CanMEDS framework used in Dutch medical education.[9] This framework recognises that, to provide optimal patient care, physicians must take responsibility for their own well-being and that of their colleagues. Several hospitals have started peer-support programmes, as this was shown to help physicians dealing with adverse events.[10]

For peer review only

Depression, anxiety and posttraumatic stress disorder

Major depressive disorder (MDD) is a common affective disorder that is characterised by a depressed mood and/or loss of pleasure.[11] A review on the epidemiology of mental disorders in Europe described a one-year prevalence of 6.9% in the general population[12]; the Nemesis-2 study showed a one-year prevalence of mood disorders of 3.0% among Dutch citizens with a high income.[13] A meta-analysis on depressive symptoms and MDD among resident physicians showed high prevalence rates (20.9% to 43.2%).[14] Fewer studies were done on depressive symptoms among medical

For peer review only - http://bmjopen.bmj.com/site/about/guidelines.xhtml

specialists, showing prevalence rates from 10.3 to 28.0%.[15–18] A recent literature review showed that as many as 40 per 100,000 physicians die by suicide each year in the United States of America, more than double the rate of the general population.[19]

Anxiety disorders include many different disorders, ranging from a specific phobia to a generalised anxiety disorder. The one-year prevalence for anxiety disorders in the Netherlands is 6.0% among citizens with a high income and 10.1% among citizens with a low income.[13]
PTSD is a highly debilitating disorder that can develop after exposure to a psychotraumatic event.[20] Symptoms of PTSD include intrusions, avoidance of stimuli related to the trauma, alterations in cognition or mood, and hyperarousal. The point prevalence of posttraumatic stress symptoms among physicians ranges between 3.8% and 15.0.[15,21–25]

The main objective of this supplementary analysis was to compare the prevalence of potential traumatic events (PTEs) and related mental health disorders between a large group of gynaecologists, paediatricians and orthopaedic surgeons. Additionally, this study investigates potential traumatic events and clinical practice related consequences. Combining the data of previous work allows us to gain statistical power to explore the association between support protocols and mental health symptoms in a larger group.

METHODS

Population and procedure

Each physician received one invitation for an online questionnaire (supplementary file 1) and two reminders over a period of 8 weeks. The questionnaire was sent through Thesistools® or Surveymonkey®, creating an anonymous (non-traceable) e-mail link. The questionnaire was sent to 1589 gynaecologists, 2160 paediatricians and 1210 orthopaedic surgeons, with a total of 4959 physicians, including residents. The invitation to participate in the questionnaire for the gynaecologists was sent in 2014, orthopaedic surgeons in 2016 and paediatricians in 2017. This is an additional analysis of combining data of previous work.

For peer review only - http://bmjopen.bmj.com/site/about/guidelines.xhtml

The membership databases of the Dutch Society of Obstetrics & Gynaecology (NVOG), Dutch Society of Orthopaedic Surgeons (NOV) and the Dutch Society of Paediatricians (NvK) were used for the invitation. Registration as a medical specialist or resident in one of these societies is obligatory and therefore most Dutch medical specialists in these fields were contacted. These databases contained residents, attending, retired and non-practicing physicians.

This study was exempted from ethical approval by the Medical Research Ethics Committees United (MEC-U), and registered under numbers W17.169 and W18.096.

Patient and public involvement

Engagement with the described medical societies was vital to ensure participation and a high response rate. The qualitative questions were piloted among several medical specialists of each specific medical specialty.

Measurements

The instruments included the Hospital Anxiety and Depression Scale (HADS) and the Trauma Screening Questionnaire (TSQ) as validated screening instruments. The HADS is a screening instrument for depression and anxiety with 14 items (4-point likert scale), where the subscales contain seven questions each. The cut-off value of the Dutch version of the depression (HADS-D) and anxiety subscale (HADS-A) is equal to or higher than eight. The total HADS cut-off value for general distress is equal or higher than 12, corresponding to clinically relevant psychological distress; this is a rather non-specific but sensitive measurement to screen for mental disorders.[26] Cut-off scores were used according to these validation studies. The sensitivity and specificity for both HADS-A and HADS-D are approximately 0.80, with Cronbach's alphas for HADS-A from 0.68 to 0.93 (mean 0.83) and for HADS-D from 0.67 to 0.90, in different populations. [27]

For peer review only

The TSQ is a ten-item screening instrument for a probable diagnosis of PTSD according to the DSM-IV, with a sensitivity of 0.94 and specificity of 0.56 when using a cut-off of equal to six or higher. The Dutch psychometric properties are validated with Cronbach's alphas from 0.71 to 0.91.[28] Only respondents who answered 'yes' to experiencing a work-related traumatic event at least four weeks ago were asked to fill out the TSQ.

Additionally, the survey contained questions about demographics, personal experiences, coping and support strategies after adverse events in the workplace. Regarding the latter, multiple-choice options were given as well as an open field where respondents were able to add their individual answers or comment on their experience. These qualitative questions were piloted among several medical specialists of all three medical specialties, to increase face value of the questions. To quantify the number of PTEs and previous posttraumatic stress symptoms, questions were added based on the criteria of the diagnosis of PTSD in the DSM-IV (questions 22 and 35, supplementary file 1).

Statistical analysis

Statistical analysis was performed using IBM Statistical Package for the Social Sciences (SPSS) version 22 and JASP version 0.8.5.1. Demographic data and multiple-choice questions were analysed using descriptive statistics and exported as frequency tables and bar charts. Differences in outcomes (in various groups) for categorical variables were tested using either a Chi-squared test or a Fisher's exact test where applicable. A two-sided p-value equal to or less than 0.05 was considered statistically significant. A multivariable logistic regression analysis was performed to estimate the effects of age, work-experience, gender and position on the likelihood that participants develop mental health outcomes, a p-value equal to or less than 0.05 was considered statistically significant. Multiple testing corrections were not performed.

For peer review only - http://bmjopen.bmj.com/site/about/guidelines.xhtml

RESULTS

A total of 1374 questionnaires were collected with a response rate of 27.7%. Table 1 shows the respondents characteristics by specialty.

Table 1: Respondent characteristics

	Total (n=4959)	Gynaecologists (n=1589)	Orthopaedic surgeons (n=1210)	Paediatricians (n=2160)
**Response rate**	27.7	48.9	21.3	29.8
**Gender**				
Male	44.9	34.7	85.6	32.7
Female	55.1	65.3	14.4	67.3
**Position**				
Resident	23.0	26.9	20.9	18.0
Attending physician	67.2	64.7	62.3	74.9
Retired	7.1	5.4	13.4	5.6
Non-practicing	2.6	3.0	3.4	1.5
**Years in practice**				
≤ 5	12.4	14.3	12.0	9.8
6-10	18.6	19.0	19.5	17.3
11-15	15.8	17.7	14.0	13.9
16-20	14.3	11.6	13.4	19.3
21-25	12.4	12.8	9.2	13.9
26-30	11.3	9.5	14.4	12.0
>30	15.1	14.7 *	17.5	13.9
**Complaints disciplinary board**	19.9	21.0	29.8	11.2
**Support protocol**				
Yes	19.6	15.8	18.8	26.3
No	42.4	52.7	30.1	34.1
I do not know	38.0	31.4	51.0	39.5

All values shown as %
*=2 missing

The majority of the orthopaedic surgeons were male (85.6%) while in the other specialties the majority were female. In all specialties most of the respondents (62.3 – 74.9%) were attending physicians, as seen in table 1. The gender and position distributions were compared with the membership databases of the societies, and for none of the specialties the distribution in the sample was significantly different from the population. The orthopaedic surgeons reported to have had the most complaints at a disciplinary board (29.8%). One hundred eight (26.3%) paediatricians reported to have a support protocol, whereas only 18.8% of the orthopaedic surgeons and 15.8% of the gynaecologists reported to have a protocol after adverse events.

For peer review only - http://bmjopen.bmj.com/site/about/guidelines.xhtml

Outcomes of the HADS and TSQ by specialty are shown in table 2.

Table 2: Depression, anxiety, psychological distress and PTSD by specialty

	Total (n=1374)	Gynaecologists (n=672)	Orthopaedic Surgeons (n=292)	Paediatricians (n=410)
**Depression**	6.4	6.5	4.8	7.3
**Anxiety**	13.6	15.8	8.2	14.1
**Psychological distress**	17.1	18.2	12.0	19.3
**PTSD**				
PTE ≥ 4 weeks ago	20.8	12.8	19.5	34.9
PTS symptoms earlier	13.4	8.2	8.6	25.4
TSQ	1.5	1.5	0.3	2.2

All values shown in % scored above HADS or TSQ cut off
Abbreviations: Potential Traumatic Event (PTE); Trauma Screening Questionnaire (TSQ); Hospital Anxiety and Depression Scale (HADS); Posttraumatic stress (PTS); Posttraumatic stress disorder (PTSD).

Table 3: Depression, anxiety and psychological distress by position (resident vs. attending) and gender

	Total (n=1240)	Depression	p	χ^2	Anxiety	P	χ^2	Psycho-logical distress	p	χ^2
**Gender**										
Male	40.8	5.5	0.075	3.2	10.1	**<0.001**	**17.2**	14.2	**<0.001**	**12.8**
Female	59.2	8.2			18.7			22.3		
**Position**										
Resident	25.4	4.7	0.112	2.5	17.1	0.125	2.3	18.4	0.887	0.0
Attending	74.6	7.4			13.6			18.1		
**Residents**										
Male	25.6	3.7	0.346	0.9	13.6	0.053	3.7	14.8	0.058	3.6
Female	74.4	5.1			18.3			19.6		
**Attending**										
Male	45.9	5.2	**0.008**	**7.1**	8.2	**<0.001**	**25.7**	12.5	**<0.001**	**22.2**
Female	54.1	9.2			18.2			22.8		

All values shown as % scored above cut-off HADS
Abbreviations: Hospital Anxiety and Depression Scale (HADS)

Eighty eight (6.4%) respondents scored above the cut-off value for depression and one hundred and eighty eight (13.6%) respondents scored above the cut-off value for anxiety. Two hundred and thirty six (17.1%) scored above the cut-off value of the total HADS, corresponding to clinically relevant

For peer review only - http://bmjopen.bmj.com/site/about/guidelines.xhtml

psychological distress. When comparing specialties, there is no significant difference in depression. However, looking at the prevalence of anxiety the orthopaedic surgeons scored significantly lower compared to both gynaecologists (15.8% vs. 8.2%, p<0.01) and paediatricians (14.1% vs. 8.2%, p<0.05). Also psychological distress was significantly lower compared to gynaecologists (18.2% vs. 12.0%, p<0.05) and paediatricians (19.3% vs. 12.0%, p<0.01).

As seen in table 3, no significant difference was found between male and female residents in rates of depression, anxiety and psychological distress. When comparing gender among attendings, the rates of depression (5.2% vs. 9.2%, p=0.019), anxiety (8.2% vs. 18.2%, p<0.001) and the total HADS (12.5·0% vs. 22.8%, p<0.001) were significantly higher among female attendings.When comparing among all respondents, the prevalence rates of anxiety (10.1% vs. 18.7%, p<0.001) and psychological distress (14.2% vs. 22.3%, p<0.001) were significantly higher among female physicians There were no differences in primary outcomes when comparing residents with attendings. Turning to the results of the logistic regression analysis, there was a significant increase in the prevalence of depression in the groups with more years in practice (OR 2.95, p=0.030, 95% CI 0.107 - 2.054, supplementary file 2), but not with increased age. We found a significant decrease in the prevalence of anxiety with more years in practice (OR 0.33, p=0.001, 95% CI -1.784 - 0.448, supplementary file 2). The highest rate of respondents with a score above the cut-off value for anxiety were found among physicians with 6-10 years in practice (supplementary file 2).

Posttraumatic stress disorder

Two hundred eighty six respondents (20.8%) reported having experienced at least one potential traumatic event (PTE) during their work, at least four weeks ago, thereby meeting DSM-IV criterion A and E. Of these respondents, 183 reported recognising posttraumatic stress (PTS) symptoms after this event (13.4% of the total group, 64.3% of the potentially traumatised group) and 20 screened positive for probable PTSD (1.5% of the total group, 7.0% of the potentially traumatised group, table 2). Of the paediatricians 34.9% reported having experienced a PTE at least four weeks ago. This is significantly higher than the percentages of the gynaecologists (12.8%, p<0.001) and orthopaedic surgeons (19.5%,

For peer review only - http://bmjopen.bmj.com/site/about/guidelines.xhtml

p<0.001), as seen in table 2. When comparing the prevalence of probable PTSD, the paediatricians had a significant higher prevalence compared to the orthopaedic surgeons (2.2% vs. 0.3%, p=0.041). Both paediatricians and gynaecologists report to recognise more PTS symptoms.

When comparing gender and position (resident vs. attending, table 4), residents are less likely to have experienced a PTE (16.8% vs. 22.6%, p=0.030) and thus also report significantly fewer PTS symptoms earlier (7.9% vs. 15.6%, p<0.001).

For peer review only

For peer review only - http://bmjopen.bmj.com/site/about/guidelines.xhtml

Table 4: Posttraumatic stress outcomes by position (resident vs. attending) and gender

	Total (n=1240)	PTE	p	χ^2	PTS symptoms earlier	p	χ^2	PTSD	p	χ^2
**Gender**										
Male	40.8	21.3	0.878	0.0	11.3	**0.044**	**4.1**	1.2	0.516	0.4
Female	59.2	21.0			15.3			1.6		
**Position**										
Resident	25.4	16.8	**0.030**	**4.7**	7.9	**<0.001**	**11.6**	0.9	0.391	0.7
Attending	74.6	22.6			15.6			1.6		
**Residents**										
Male	25.6	21.0	0.245	1.3	4.9	0.246	1.3	0.0	0.306	1.0
Female	74.4	15.3			8.9			1.3		
**Attending**										
Male	45.9	21.4	0.377	0.8	12.5	**0.006**	**7.6**	1.4	0.371	0.8
Female	54.1	23.6			18.2			1.8		

All values shown as %, PTSD is shown as scored above cut-off TSQ
Abbreviations: Potential Traumatic Event (PTE); Trauma Screening Questionnaire (TSQ); Hospital Anxiety and Depression Scale (HADS); Posttraumatic stress (PTS); Posttraumatic stress disorder (PTSD).

The highest rate of probable PTSD was found among attendings (n=15, 1.6%) compared to three of the residents (0.9%). When comparing gender, male and female physicians report the same number of PTEs, but female physicians are more likely to report earlier PTS-symptoms (15.3% vs. 11.3%, p=0.044).

Those who witnessed a PTE, reported significantly more defensive work changes after an incident (28.0% vs 20.5%, p=0.007). Respondents who recognised PTS-symptoms after an incident considered quitting medical work more often (42.6% vs. 29.2%, p=0.029). Those with current probable PTSD significantly considered quitting medical work more often (60.0% vs. 35.8%, p=0.032).
When evaluating the presence of a support protocol, this was found not to influence the primary mental health outcomes, except for probable PTSD. Participants who responded that there was no

For peer review only - http://bmjopen.bmj.com/site/about/guidelines.xhtml

support protocol at their work-place after an adverse event, were significantly more likely to report probable PTSD after a PTE (p=0.022).

DISCUSSION

Depression and anxiety

This additional analysis combined data from previous work to compare the point prevalence rates of probable depression, anxiety, psychological distress, work-related PTE and PTSD among gynaecologists, orthopaedic surgeons and paediatricians. It is part of the Work-related Adverse and Traumatic Events Research (WATER) and this paper combines the results of previous studies [6,8], to gain statistical power and show differences between support protocols, specialties, gender and position. Our point prevalence rates of depression (6.4%) and anxiety (13.6%), are high compared to 12-month prevalence rates of mood disorders (3.0%) and anxiety (6.0%) in high income populations in the Netherlands, and compared to European 12-month prevalence rate of anxiety (6.4%).[12,13] The point prevalence rates in this study are comparable to American 12-month general prevalence rates of depression (6.7%) and anxiety (18.1%).[29,30] This indicates that physicians are more depressed and anxious compared to the general population and the population with high income in the Netherlands. This high prevalence is in line with current literature, where even higher prevalence rates for depression and anxiety were found among medical students, residents and specialists.[14,15,31] One of the relevant findings of this study is the high prevalence of psychological distress (17.6%). Scoring above this cut-off is not specific for one diagnosis, but does indicate a high level of distress and a risk for potential unfit physicians. We found a significant increase in the prevalence of depression corresponding with more years in practice. Furthermore, this study showed a significantly higher prevalence of depression and anxiety among female compared to male attendings. This finding correlates with previous study results,[32] where prevalence rates for depression and anxiety were higher among British female doctors compared to males. These findings are also in line with epidemiologic data on the gender distribution of mood and anxiety disorders in the general population.[12,13] This finding is relevant, because of the feminization of medical specialties globally, and an increase in female physicians of 1.0 to 2.0 % each year in the last ten years in the

For peer review only - http://bmjopen.bmj.com/site/about/guidelines.xhtml

Netherlands.[33] These results may implicate that a change is needed in support after adverse events, that is sensitive to the increase in women working in the field of medicine.

Posttraumatic stress disorder

In this sample, 285 respondents (20.8%) reported to have experienced a potential traumatic event (PTE) that took place longer than four weeks ago. This is within the range of the estimated prevalence of second victims of 10.4% and 43.3%, reported by Seys et al.[34] Our prevalence for work-related PTSD (1.5%) is slightly higher, compared to the general Dutch prevalence of PTSD (1.2%).[35] The Dutch prevalence of only work-related PTSD is not known, however assumed to be lower. Of the potential traumatised respondents, 7.0% screened positive for PTSD, which is comparable to other occupations with mental health hazards, such as police officers after critical police incidents (7.0%).[36]

One of the key findings of this supplementary analysis, is that after experiencing a PTE, the absence of a support protocol is significantly associated with more probable PTSD (p=0.022). This once again shows the importance of support protocols and implicates that a well perceived support system may prevent posttraumatic stress complaints. This survey did however not explore the types of support protocols (e.g. debriefing versus peer-support) in place, as they may differ across hospitals and even within one hospital across specialties. Therefore nothing can be concluded of the type of support protocol that may help prevent PTS symptoms. However, some form of formalised support may lead to seeking- and finding professional help earlier. Studies have shown that the threshold to look for professional mental health as a physician is high. Physicians report a reluctance to seek care, due to barriers such as too little time, fear of losing their medical license and stigma.[37] A (semi) compulsory support protocol after a PTE could potentially increase awareness, lower this threshold and guide physicians in need towards professional support or treatment. Debriefing and hospital based peer-support have shown useful and effective after PTEs in several small studies. [10,38] Also, this awareness of more workplace support may lead to perceiving adverse events less psychotraumatic, however having a support protocol was not correlated with less PTEs.

For peer review only - http://bmjopen.bmj.com/site/about/guidelines.xhtml

Of the residents who filled out the TSQ, three screened positive for PTSD (all female respondents). These findings are in line with the known gender distribution, as women are more likely to develop PTSD than men.[39] Other studies found higher prevalence rates of PTSD (i.e. Joseph[21] = 15%, Mills[24] = 11.9%, Ruitenburg[15] =15%, and Shi[22] = 3.8%), but they used different (non-) validated screening instruments and methods. None of these screening instruments assessed when the PTE happened (for a diagnosis of PTSD this needs to be at least four weeks ago), meaning that their respondents may not meet criterion A of PTSD and could also be suffering from acute stress symptoms. Furthermore, these studies did not assess whether the potential event was work-related, so these numbers show general PTSD prevalence rates which are usually higher.

Studies have shown that dysfunctional coping can predict complicated grief and PTSD severity.[40,41] Thus, it is important to educate physicians about coping mechanisms and stress management techniques to prevent adverse mental health conditions.[42] With social support being an important protective factor for PTSD[43], the role of educating health care professionals to address this problem and support their colleagues in peer-support groups is crucial.[10]

Job related consequences of depression, anxiety, adverse events and PTSD

This study shows that physicians consider quitting their job more often, after having experienced PTS-symptoms in the past or currently suffering from probable PTSD. Experiencing a PTE is also associated with more defensive medical practice. When looking at the prevalence of complaints at a disciplinary board, the orthopaedic surgeons reported the most complaints (table 1). This may be due to the higher amount of elective work they do, which usually leads to more dissatisfied patients, compared to emergency work. More complaints was not associated with considering quitting work or increased rates of depression, anxiety or psychological distress.

For peer review only - http://bmjopen.bmj.com/site/about/guidelines.xhtml

Strengths

Little research has been done on PTSD in physicians, in particular with regard to mental health in relation to social and occupational functioning and clinical practice related consequences. We incorporated the A and E criterion in our probable PTSD diagnosis, therefore our prevalence is more likely to reflect the real prevalence of PTSD. Also, this study included a large number (n=1374) of physicians, which made it possible to analyse gender, position and age differences. The overall response rate among gynaecologists was high (42.9%), followed by the orthopaedic surgeons (29%) and the paediatricians (18.9%).

Limitations

There are several limitations to this study. The population of this study is limited to three specialties and one country in Europe and thus it is difficult to translate these results to physicians worldwide. However, it is known that working conditions and related stress levels are comparable with the rest of Europe and North-America. Also, prevalence rates reported in this study are possibly not comparable to other studies on mental health, as many different samples and measurements are used in mental health studies. Furthermore, avoidance is one of the key symptoms of PTSD and can therefore lead to non-response and non-completion of the survey, as it may trigger symptoms of PTSD. Therefore the prevalence rates found in psychotrauma studies are often an underestimation of the PTSD-prevalence. On the other hand, this self-selection bias may also lead to overrepresentation of physicians with strong opinions. One suggestion to decrease this non-completion bias, is to give respondents the opportunity to finish the survey at a later time point and receive a reminder. The overall response rate was 27.7%, which is slightly higher than the average response rate for email surveys (25.0%)[44] and comparable to similar surveys in this field [45]. Due to the low numbers of PTSD, it was not possible to correlate the type of adverse event with more PTSD. Furthermore, the HADS and TSQ are validated screening instruments, however they only give an estimate of the prevalence rates of probable disorders as they do not cover all the symptom domains of the disorders. Only the prevalence of PTSD is work-related, the numbers for depression and anxiety are general prevalence rates.

For peer review only - http://bmjopen.bmj.com/site/about/guidelines.xhtml

For peer review only

CONCLUSION

Physicians are often exposed to PTEs over the course of their career with potentially high emotional impact. The prevalence rates for probable depression (6.4%) and anxiety (13.6%) were higher among physicians, compared to the general population with high income in the Netherlands. The point prevalence of clinical psychological distress was 19.0% and of probable PTSD was 1.5%. Female physicians are significantly more prone to develop depressive- and anxiety disorders than their male colleagues. PTEs were associated with more defensive practice and dissatisfaction about work. And importantly, the absence of a support protocol was associated with more probable PTSD. This once again shows the importance of support protocols and implicates that a well perceived support system, such as peer support and debriefings, may prevent posttraumatic stress complaints and unfit physicians.

For peer review only

For peer review only - http://bmjopen.bmj.com/site/about/guidelines.xhtml

ACKNOWLEDGEMENTS

We would like to thank the Dutch Society of Obstetrics & Gynaecology (NVOG), Dutch Society of Orthopaedic Surgeons (NOV), the Dutch Society of Paediatricians (NvK), and their members, for their cooperation in this study.

For peer review only

For peer review only - http://bmjopen.bmj.com/site/about/guidelines.xhtml

COMPETING INTERESTS

None of the authors declare a conflict of interest.

For peer review only

FUNDING

This research received no specific grant from any funding agency in the public, commercial or not-for-profit sectors.

For peer review only

For peer review only - http://bmjopen.bmj.com/site/about/guidelines.xhtml

CONTRIBUTORSHIP

KS, MvS, CS, MvP were involved in the design of this study. KS and HP analysed the data and made the first draft. KS, HP, MvS, CS, MO and MvP were involved in finalising the manuscript.

For peer review only

For peer review only - http://bmjopen.bmj.com/site/about/guidelines.xhtml

DATA AVAILABILITY

The data can be made available by the corresponding author upon reasonable request and for a well defined purpose. The request will be discussed in our research group. The data will be de-identified participant data. A statistical analysis plan is available.

For peer review only

For peer review only - http://bmjopen.bmj.com/site/about/guidelines.xhtml

REFERENCES

1 Yehuda R, Vermetten E, McFarlane AC, *et al.* PTSD in the military: Special considerations for understanding prevalence, pathophysiology and treatment following deployment. *Eur J Psychotraumatol* Published Online First: 2014. doi:10.3402/ejpt.v5.25322

2 van der Meer CAI, Bakker A, Schrieken BAL, *et al.* Screening for trauma-related symptoms via a smartphone app: The validity of Smart Assessment on your Mobile in referred police officers. *Int J Methods Psychiatr Res* Published Online First: 2017. doi:10.1002/mpr.1579

3 Mellotte H, Murphy D, Rafferty L, *et al.* Pathways into mental health care for UK veterans: a qualitative study. *Eur J Psychotraumatol* Published Online First: 2017. doi:10.1080/20008198.2017.1389207

4 Edrees H, Federico F. Supporting clinicians after medical error. *BMJ* 2015. doi:10.1136/bmj.h1982

5 Devi S. Doctors in distress. *Lancet* Published Online First: 2011. doi:10.1016/S0140-6736(11)60145-1

6 van Steijn ME, Scheepstra KWF, Yasar G, *et al.* Occupational well-being in pediatricians—a survey about work-related posttraumatic stress, depression, and anxiety. *Eur J Pediatr* Published Online First: 2019. doi:10.1007/s00431-019-03334-7

7 Kerkman T, Dijksman LM, Baas MAM, *et al.* Traumatic Experiences and the Midwifery Profession: A Cross-Sectional Study Among Dutch Midwives. *J Midwifery Women's Heal* Published Online First: 2019. doi:10.1111/jmwh.12946

8 Baas MAM, Scheepstra KWF, Stramrood CAI, *et al.* Work-related adverse events leaving their mark: A cross-sectional study among Dutch gynecologists. *BMC Psychiatry* 2018;**18**. doi:10.1186/s12888-018-1659-1

9 Frank JR, Snell L, Sherbino J E. CanMEDs 2015 Physician Competency Framework. CanMEDS 2015 Physician Competency Fram. Ottawa R. Coll. Physicians Surg. Canada. 2015;:1–30.

10 Hu YY, Fix ML, Hevelone ND, *et al.* Physicians' needs in coping with emotional stressors: The case for peer support. *Arch Surg* Published Online First: 2012. doi:10.1001/archsurg.2011.312

11 American Psychiatric Association. Diagnostic and Statistical Manual of Mental Disorders: DSM-5. *Am Psychiatr Assoc* 2013;:991. doi:10.1176/appi.books.9780890425596.744053

12 Alonso J, Angermeyer MC, Bernert S, *et al.* Prevalence of mental disorders in Europe: results from the European Study of the Epidemiology of Mental Disorders (ESEMeD) project. *Acta Psychiatr Scand Suppl* 2004;**109**:21–7. doi:10.1111/j.1600-0047.2004.00327.x

13 De Graaf R, Ten Have M, Van Gool C, *et al.* Prevalence of mental disorders and trends from 1996 to 2009. Results from the Netherlands Mental Health Survey and Incidence Study-2. Soc. Psychiatry Psychiatr. Epidemiol. 2012;**47**:203–13. doi:10.1007/s00127-010-0334-8

14 Mata DA, Ramos MA, Bansal N, *et al.* Prevalence of Depression and Depressive Symptoms Among Resident Physicians: A Systematic Review and Meta-analysis. *JAMA* 2015;**314**:2373–83. doi:10.1001/jama.2015.15845

For peer review only - http://bmjopen.bmj.com/site/about/guidelines.xhtml

15 Ruitenburg MM, Frings-Dresen MH, Sluiter JK. The prevalence of common mental disorders among hospital physicians and their association with self-reported work ability: a cross-sectional study. *BMC Health Serv Res* 2012;**12**:292. doi:10.1186/1472-6963-12-292

16 Wurm W, Vogel K, Holl A, *et al.* Depression-burnout overlap in physicians. *PLoS One* Published Online First: 2016. doi:10.1371/journal.pone.0149913

17 Hsu K, Marshall V. Prevalence of depression and distress in a large sample of Canadian residents, interns, and fellows. *Am J Psychiatry* Published Online First: 1987. doi:10.1176/ajp.144.12.1561

18 Tomioka K, Morita N, Saeki K, *et al.* Working hours, occupational stress and depression among physicians. *Occup Med (Chic Ill)* Published Online First: 2011. doi:10.1093/occmed/kqr004

19 O T, A A, D T, *et al.* Physician Suicide: A Silent Epidemic. In: *In: APA annual meeting, Poster no 227, New York City*. 2018. 123–4.

20 Kessler RC, Aguilar-Gaxiola S, Alonso J, *et al.* Trauma and PTSD in the WHO World Mental Health Surveys. Eur. J. Psychotraumatol. 2017. doi:10.1080/20008198.2017.1353383

21 Joseph B, Pandit V, Hadeed G, *et al.* Unveiling posttraumatic stress disorder in trauma surgeons. *J Trauma Acute Care Surg* 2014;**77**:148–54. doi:10.1097/TA.0000000000000271

22 Shi L, Wang L, Jia X, *et al.* Prevalence and correlates of symptoms of post-traumatic stress disorder among Chinese healthcare workers exposed to physical violence: a cross-sectional study. *BMJ Open* 2017;**7**:e016810. doi:10.1136/bmjopen-2017-016810

23 Chan AOM, Huak CY. Influence of work environment on emotional health in a health care setting. *Occup Med (Lond)* 2004;**54**:207–12. doi:10.1093/occmed/kqh062

24 Mills LD, Mills TJ. Symptoms of post-traumatic stress disorder among emergency medicine residents. J. Emerg. Med. 2005. doi:10.1016/j.jemermed.2004.05.009

25 Wahlberg, Andreen Sachs M, Johannesson K, *et al.* Post-traumatic stress symptoms in Swedish obstetricians and midwives after severe obstetric events: a cross-sectional retrospective survey. *BJOG An Int J Obstet Gynaecol* Published Online First: 2017. doi:10.1111/1471-0528.14259

26 Spinhoven P, Ormel J, Sloekers PP, *et al.* A validation study of the Hospital Anxiety and Depression Scale (HADS) in different groups of Dutch subjects. *Psychol Med* 1997;**27**:363–70. doi:10.1017/S0033291796004382

27 Bjelland I, Dahl AA, Haug TT, *et al.* The validity of the Hospital Anxiety and Depression Scale. An updated literature review. *J Psychosom Res* 2002.

28 Dekkers AMM, Olff M, Maring GWB. Identifying persons at risk for PTSD after trauma with TSQ in the Netherlands. *Community Ment Health J* 2010;**46**:20–5. doi:10.1007/s10597-009-9195-6

29 Kessler RC, Chiu WT, Demler O, *et al.* Prevalence, severity, and comorbidity of 12-month DSM-IV disorders in the National Comorbidity Survey Replication. *Arch Gen Psychiatry* 2005;**62**:617–27. doi:10.1001/archpsyc.62.6.617

30 Substance Abuse and Mental Health Services Administration, Center for Behavioural Health Statistics and Quality. Results from the 2015 National Survey on Drug Use and Health: Detailed Tables,. 2015.

31 Hope V, Henderson M. Medical student depression, anxiety and distress outside North

For peer review only - http://bmjopen.bmj.com/site/about/guidelines.xhtml

America: a systematic review. *Med Educ* 2014;**48**:963–79. doi:10.1111/medu.12512

32 Wall TD, Bolden RI, Borrill CS, *et al.* Minor psychiatric disorder in NHS trust staff: Occupational and gender differences. *Br J Psychiatry* 1997;**171**:519–23. doi:10.1192/bjp.171.6.519

33 Capaciteitsorgaan. Capaciteitsplan 2016 (in Dutch). Utrecht: 2016. www.capaciteitsorgaan.nl accessed 2-7-2018

34 Seys D, Wu AW, Gerven E Van, *et al.* Health Care Professionals as Second Victims after Adverse Events. *Eval Health Prof* 2013;**36**:135–62. doi:10.1177/0163278712458918

35 De Vries GJ, Olff M. The lifetime prevalence of traumatic events and posttraumatic stress disorder in the Netherlands. *J Trauma Stress* Published Online First: 2009. doi:10.1002/jts.20429

36 Carlier I V, Lamberts RD, Gersons BP. Risk factors for posttraumatic stress symptomatology in police officers: a prospective analysis. *J Nerv Ment Dis* Published Online First: 1997. doi:10.1097/00005053-199708000-00004

37 Dyrbye LN, West CP, Sinsky CA, *et al.* Medical Licensure Questions and Physician Reluctance to Seek Care for Mental Health Conditions. *Mayo Clin Proc* Published Online First: 2017. doi:10.1016/j.mayocp.2017.06.020

38 Baverstock A, Finlay F. Specialist registrars' emotional responses to a patient's death. *Arch Dis Child* Published Online First: 2006. doi:10.1136/adc.2005.076760

39 Olff M, Langeland W, Draijer N, *et al.* Gender differences in posttraumatic stress disorder. [Review] [287 refs]. *Psychol Bull* Published Online First: 2007. doi:10.1037/0033-2909.133.2.183

40 Schnider KR, Elhai JD, Gray MJ. Coping style use predicts posttraumatic stress and complicated grief symptom severity among college students reporting a traumatic loss. *J Couns Psychol* 2007;**54**:344–50. doi:10.1037/0022-0167.54.3.344

41 Kerai SM, Khan UR, Islam M, *et al.* Post-traumatic stress disorder and its predictors in emergency medical service personnel: a cross-sectional study from Karachi, Pakistan. *BMC Emerg Med* 2017;**17**:26. doi:10.1016/j.brat.2003.07.010

42 Brosschot JF. Ever at the ready for events that never happen. *Eur J Psychotraumatol* Published Online First: 2017. doi:10.1080/20008198.2017.1309934

43 Olff M. Bonding after trauma: On the role of social support and the oxytocin system in traumatic stress. *Eur J Psychotraumatol* Published Online First: 2012. doi:10.3402/ejpt.v3i0.18597

44 Fluid Surveys University. Response Rate Statistics for Online Surveys - What Numbers Should You be Aiming For? 2014.

45 Shanafelt TD, Boone S, Tan L, *et al.* Burnout and satisfaction with work-life balance among US physicians relative to the general US population. *Arch Intern Med* Published Online First: 2012. doi:10.1001/archinternmed.2012.3199

For peer review only - http://bmjopen.bmj.com/site/about/guidelines.xhtml

For peer review only

For peer review only - http://bmjopen.bmj.com/site/about/guidelines.xhtml

Supplementary files

1. Questionnaire
2. Supplementary table: logistic regression work experience vs. HADS-D and HADS-A

For peer review only

For peer review only - http://bmjopen.bmj.com/site/about/guidelines.xhtml

Supplementary file 1: Questionnaires

Paediatricians

1 What is your gender?
1. Male
2. Female

2 What is your age?
1. 20-29 years
2. 30-39 years
3. 40-49 years
4. 50-59 years
5. 60-69 years
6. ≥ 70 years

3 Are you a member of the Dutch Society of Paediatricians (NvK)?
1. Yes
2. No

4 What is your current position?
1. Resident
2. Attending physician
3. Retired
4. Non-practicing/management

5 How many years have you been working in the paediatrics field as a physician? (Including as intern)
1. ≤ 5 years
2. 6-10 years
3. 11-15 years
4. 16-20 years
5. 21-25 years
6. 26-30 years
7. > 30 years

6 At the moment, are you working in a general hospital or in an academic referral centre?
1. General hospital
2. Academic referral centre
3. Both
4. I do not work anymore, but I used to work in a general hospital
5. I do not work anymore, but I used to work in an academic referral centre

7 At which department do you work the most? (If you are not practicing anymore, fill in where you used to work)
1. Neonatology
2. Emergency room
3. General paediatrician
4. NICU
5. Child intensive care
6. Policlinic
7. Children's oncology
8. Other…

8 What do you consider to be an adverse event on the work-floor? (More than one answer possible)
1. Not applicable

For peer review only

2. Bad news conversation/interview
3. (Critically) ill neonate
4. (Critically) ill children
5. When a patient dies
6. When you miss a diagnosis
7. When you have to refuse a patient
8. When you are in doubt whether you are making the right decision
9. When an inexperienced colleague is on call
10. When you decide on an abstaining course of treatment
11. Treatment of neonates
12. When you know a patient will remain severely handicapped
13. Handling the emotions of the parents
14. (Suspicion of) child abuse
15. Other …

9 How do you cope with adverse events on the work-floor? (More than one answer possible)

1. Not applicable / never experienced
2. Seeking professional help
3. Use new medication
4. Drink (more) alcohol
5. Smoke (more) cigarettes
6. Use (more) drugs
7. Work out (more)
8. Going home as soon as possible
9. Call in sick
10. Hide your emotions
11. Find a distraction
12. Praying or other religious activities
13. Talking to friends and/or family
14. Informally discussing the matter with peers/colleagues
15. Quitting
16. Work less
17. Not working any evening/night shifts
18. Other …

10 The current support organized by your institution after an adverse event is good:

1. Strongly disagree
2. Disagree
3. Agree
4. Strongly agree

11 There is plenty of room to informally discuss adverse events in the department/partnership:

1. Strongly disagree
2. Disagree
3. Agree
4. Strongly agree

12 There is plenty of room to informally discuss adverse events in the hospital:

1. Strongly disagree
2. Disagree
3. Agree
4. Strongly agree

13 There should be a culture change regarding support after an adverse event:

1. Strongly disagree

For peer review only

2. Disagree
3. Agree
4. Strongly agree

14 There is a protocol at your institution regarding support after an adverse event:
1. Yes
2. Yes, but nobody uses it
3. No, but there is one currently being created
4. No
5. I do not know

15 Support after an adverse event consisted of: (more than one answer possible)
1. I have never experienced an adverse event
2. There was none
3. Professionally organised peer-support
4. Self-initiated peer-support with direct colleagues (own department)
5. Self-initiated peer-support with indirect colleagues (different department)
6. Conversation(s) with a psychologist or coach
7. Evaluation with the present team
8. Help from the medical officer after a sick-leave
9. Other …

16 Your preferred support after an adverse event would be: (more than one answer possible)
1. Not applicable
2. Support is unnecessary
3. Professionally organised peer-support
4. Evaluation with the present team
5. Peer-support from direct colleagues (own department)
6. Peer-support from indirect colleagues (different department)
7. Mindfulness
8. Other …

17 It should be mandatory for the hospital to organise support after an adverse event:
1. Yes
2. No

18 You have learned to cope with adverse events through/during:
1. Med-school (without clerkships)
2. Clerkships
3. Internships
4. Residency
5. Attending physician
6. Other study
7. Psychological help
8. Specific course/training
9. Peer-review
10. Mindfulness
11. Never learnt
12. Other…

19 In the course of your career, you have become more defensive:
1. Strongly disagree
2. Disagree
3. Agree
4. Strongly agree

For peer review only

20 Have you changed your working-conditions (e.g. less shifts, more diagnostic tests) after experiencing a patient-related adverse event?
1. Yes
2. No

21 What did you change? (More than one answer possible)
1. Work less
2. Less evening/night-shifts
3. No evening/night-shifts anymore
4. Calling a colleague sooner
5. Quit my job
6. More diagnostic tests
7. Start treatment sooner
8. Other…

22 Sometimes people experience traumatic events, such as live-threatening situations as a cause of a natural disaster, high-impact trauma or fire; being attacked or raped; witness a murder, death or hear find out someone close to them experienced something terrible. As a physician, one can experience such events in patient-care: critical illness or death of a patients, severe injury, as well as violent behaviour from a patient or their family.
Have you ever, during your work AS A PHYSICIAN experienced such (adverse) events/incidents?
1. Yes
2. No

23 Can you briefly describe the adverse event(s)?
1. Yes, please describe it…
2. No

24 Did (some part of) this/these adverse event(s) take place more than 4 weeks ago?
1. Yes
2. No

Followed by the TSQ questionnaire.

35 It might be that you did not experience the reactions and emotions as described on the previous pages, but that you recognize them from a past period in your life. For example, having disturbing thoughts or dreams, reliving the event, getting upset. Do you recognize these reactions?
1. Yes
2. No

36 Which of these reactions do you recognize? (More than one answer possible)
1. Disturbing thoughts
2. Disturbing dreams
3. Reliving experiences
4. Getting upset
5. Physical responses
6. Insomnia
7. Irritability
8. Trouble focussing
9. Hyper arousal
10. Tensed

37 How long did these symptoms last?
1. < 4 weeks

For peer review only

2. > 4 weeks
3. > 6 months
4. > 1 year

38 Have you ever been diagnosed with PTSD (posttraumatic stress-disorder)?

1. Yes
2. No

Followed by the HADS questionnaire.

53 Have you ever seriously considered quitting your job as a paediatrician? (e.g. by looking for a different job, talking to human resources about ending your contract?

1. Yes, absolutely
2. Yes, sometimes
3. No, never

54 What was the reason to consider quitting? (More than one answer possible)

1. Bad collaboration (working together) with a co-worker
2. Not challenging enough within the field
3. A lot of complications
4. Afraid to make mistakes
5. Too much responsibility
6. Traumatic experience on the work-floor
7. Complaint(s) from patients
8. One-sided
9. A lot of stress
10. A high workload
11. Disbalance between work and private life
12. Disagreements with work providers
13. Different interests
14. New challenge
15. Too much administration
16. Too much bureaucracy
17. Patient-violence
18. Too many rules
19. Insufficient guidance from supervisors
20. Problems with the partnership
21. Private reasons
22. Disutility
23. Other…

55 Did you ever faced a complaint at the disciplinary board?

1. Yes
2. No

Gynaecologists

1 Gender

1. Female
2. Male

2 Age

1. 25-34 years
2. 35-44 years

For peer review only

3. 45-54 years
4. 55-64 years
5. 65 years and older

3 Current position
1. Resident
2. Attending / fellow
3. Retired gynaecologist
4. Non-practicing/management

4 How many years have you been working as a gynaecologist? (Including working as a resident)

5 I considered quitting my job as a gynaecologist:
1. Strongly disagree
2. Disagree
3. Agree
4. Strongly agree

6 In case you considered quitting, what was the most important reason?
1. Most important reason: …
2. Never considered quitting

7 Did you ever face a complaint at the disciplinary board?
1. Yes
2. No

8 What do you consider to be an adverse event on the work-floor? (More than one answer possible)
1. Not applicable
2. Bad news conversations/interview
3. The critical moments when a patient's life is at risk
4. When a patient dies
5. When you miss a diagnosis
6. When you feel like you cannot help the patients
7. When you are in doubt whether you are making the right decision
8. Other …

9 After experiencing adverse events on the work-floor, I do the following: (More than one answer possible)
1. Not applicable
2. Seeking professional help
3. Going home as soon as possible
4. Drink more alcohol, use more drugs and smoke more cigarettes than I normally would do
5. Use new medication I barely or normally not use
6. Trying not to think about it
7. Find a distraction
8. Praying or other religious activities
9. Formal case review
10. Work out or doing my hobby
11. Talking to friends and/or family
12. Informally discussing the matter with peers/colleagues
13. I developed symptoms of a burn-out (e.g. emotional exhaustion, depersonalisation, lower self-esteem)
14. Call in sick
15. Quit my job
16. Other …

For peer review only

10 The current support organized by your institution after an adverse event is good:
1. Strongly disagree
2. Disagree
3. Agree
4. Strongly agree

11 There is plenty of room to informally discuss adverse events in the department/partnership:
1. Strongly disagree
2. Disagree
3. Agree
4. Strongly agree

12 You have learned to cope with adverse events through/during:
1. Clerkships
2. Residency
3. Specific course/training
4. Intervision
5. Other …
6. Never really learned it

13 After experiencing an adverse event I changed my working conditions (e.g. no more shifts, no more vaginal breech births, not performing any surgeries alone, coming to the hospital sooner instead of supervision over the telephone)
1. Strongly disagree
2. Disagree
3. Agree
4. Strongly agree

14 In the course of your career, you have become more defensive:
1. Strongly disagree
2. Disagree
3. Agree
4. Strongly agree

15 Support after an adverse event consisted of… (fill in the box)

16 There is a protocol at your institution regarding support after an adverse event:
1. No
2. I do not know
3. Yes, consisting of…

17 Your preferred support after an adverse event would be: (more than one answer possible)
1. Evaluation with direct colleagues
2. Intervision with indirect colleague's (physicians with a different specialty)
3. Talking to a psychologist or coach
4. A buddy
5. Other …

18 When you lay awake at night, you are not made for this job:
1. Strongly disagree
2. Disagree
3. Agree
4. Strongly agree

For peer review only

19 There should be a culture change regarding support after an adverse event:
1. Strongly disagree
2. Disagree
3. Agree
4. Strongly agree

20 Sometimes people experience traumatic events, such as live-threatening situations as a cause of a natural disaster, high-impact trauma or fire; being attacked or raped; witness a murder, death or hear find out someone close to them experienced something terrible. As a physician, one can experience such events in patient-care: critical illness or death of a patients, severe injury, as well as violent behaviour from a patient or their family.
1) Have you ever, during your work AS A PHYSICIAN experienced such (adverse) events/incidents?
2) After at least one of those adverse events, did you experience intense fear, helplessness or abomination?
3) Did this/these event(s) happened at least 4 weeks ago?
1. I answered yes to all of these questions
2. No, I did not answer yes to all these questions

Followed by the TSQ

31 Can you briefly describe the adverse event(s)?

32 It might be that you did not experience the reactions and emotions as described on the previous pages, but that you recognize them from a past period in your life. For example, having disturbing thoughts or dreams, reliving the event, getting upset. Do you recognize these reactions?
1. Yes
2. No

Followed by the HADS.

Othopaedic surgeons

1 What is your gender?
1. Male
2. Female

2 What is your age?
1. 20-29 years
2. 30-39 years
3. 40-49 years
4. 50-59 years
5. 60-69 years
6. ≥ 70 years

3 Are you a member of the Dutch Orthopaedic Society (NOV)?
1. Yes
2. No

4 What is your current position?
1. Resident
2. Attending physician
3. Retired
4. Non-practicing/management

For peer review only

5 How many years have you been working in the orthopaedic field as a physician? (Including as intern)

1. ≤ 5 years
2. 6-10 years
3. 11-15 years
4. 16-20 years
5. 21-25 years
6. 26-30 years
7. > 30 years

6 What do you consider to be an adverse event on the work-floor? (More than one answer possible)

1. Not applicable
2. Bad news conversation/interview
3. (Critically) ill patients
4. When a patient dies
5. When you miss a diagnosis
6. When you have to refuse a patient
7. When you are in doubt whether you are making the right decision
8. When an inexperienced colleague is on call
9. When you decide on an abstaining course of treatment
10. Treatment of young patients
11. When you know a patient will remain severely handicapped
12. Other …

For peer review only

7 How do you cope with adverse events on the work-floor? (More than one answer possible)

1. Not applicable / never experienced
2. Seeking professional help
3. Use new medication
4. Drink (more) alcohol
5. Smoke (more) cigarettes
6. Use (more) drugs
7. Work out (more)
8. Going home as soon as possible
9. Call in sick
10. Hide your emotions
11. Find a distraction
12. Praying or other religious activities
13. Talking to friends and/or family
14. Informally discussing the matter with peers/colleagues
15. Quitting
16. Work less
17. Other …

8 The current support organized by your institution after an adverse event is good:

1. Strongly disagree
2. Disagree
3. Agree
4. Strongly agree

9 There is plenty of room to informally discuss adverse events in the department/partnership:

1. Strongly disagree
2. Disagree
3. Agree
4. Strongly agree

10 There is plenty of room to informally discuss adverse events in the hospital:
1. Strongly disagree
2. Disagree
3. Agree
4. Strongly agree

11 There should be a culture change regarding support after an adverse event:
1. Strongly disagree
2. Disagree
3. Agree
4. Strongly agree

12 There is a protocol at your institution regarding support after an adverse event:
1. Yes
2. Yes, but nobody uses it
3. No, but there is one currently being created
4. No
5. I do not know

13 Support after an adverse event consisted of: (more than one answer possible)
1. I have never experienced an adverse event
2. There was none
3. Professionally organised peer-support
4. Self-initiated peer-support with direct colleagues (own department)
5. Self-initiated peer-support with indirect colleagues (different department)
6. Conversation(s) with a psychologist or coach
7. Evaluation with the present team
8. Help from the medical officer after a sick-leave
9. Other …

14 Your preferred support after an adverse event would be: (more than one answer possible)
1. Not applicable
2. Support is unnecessary
3. Professionally organised peer-support
4. Evaluation with the present team
5. Peer-support from direct colleagues (own department)
6. Peer-support from indirect colleagues (different department)
7. Mindfulness
8. Other …

15 It should be mandatory for the hospital to organise support after an adverse event:
1. Yes
2. No

16 You have learned to cope with adverse events through/during:
1. Med-school (without clerkships)
2. Clerkships
3. Internships
4. Residency
5. Attending physician
6. Psychological help
7. Specific course/training
8. Peer-review
9. Mindfulness
10. Never learnt

For peer review only

17 In the course of your career, you have become more defensive:
1. Strongly disagree
2. Disagree
3. Agree
4. Strongly agree

18 Have you changed your working-conditions (e.g. less shifts, more diagnostic tests) after experiencing a patient-related adverse event?
1. Yes
2. No

19 What did you change? (More than one answer possible)
1. Work less
2. Less evening/night-shifts
3. No evening/night-shifts anymore
4. Calling a colleague sooner
5. Quit
6. Start treatment sooner
7. Other…

20 Have you ever been diagnosed with PTSD (posttraumatic stress-disorder)?
1. Yes
2. No

21 Sometimes people experience traumatic events, such as live-threatening situations as a cause of a natural disaster, high-impact trauma or fire; being attacked or raped; witness a murder, death or hear find out someone close to them experienced something terrible. As a physician, one can experience such events in patient-care: critical illness or death of a patients, severe injury, as well as violent behaviour from a patient or their family.
Have you ever, during your work AS A PHYSICIAN experienced such (adverse) events/incidents?
1. Yes
2. No

22 Did this/these take place less than 4 weeks ago?
1. Yes
2. No

23 Can you describe the event/incident?
1. Yes
2. No

Followed by the TSQ and HADS questionnaire.

51 Have you ever seriously considered quitting your job as an orthopaedic surgeon? (e.g. by looking for a different job, talking to human resources about ending your contract?
1. Yes
2. No

52 What was the reason to consider quitting? (More than one answer possible)
1. Bad collaboration (working together) with a co-worker
2. Not challenging enough within the field
3. A lot of complication
4. Afraid to make mistakes
5. Too much responsibility

For peer review only

6. Traumatic experience on the work-floor
7. Complaint(s) from patients
8. One-sided
9. A lot of stress
10. A high workload
11. Disbalance between work and private life
12. Disagreements with work providers
13. Different interests
14. New challenge
15. Too much administration
16. Too much bureaucracy
17. Patient-violence
18. Too many rules
19. Insufficient guidance from supervisors
20. Problems with the partnership
21. Private reasons
22. Disutility
23. Other…

53 Did you ever face a complaint at the disciplinary board?

1. Yes
2. No

For peer review only

Supplementary file 2: logistic regression model and odds ratios 'work experience – HADS-D and HADS-A'

HADS-D

	Estimate	Standard Error	Odds Ratio	z	p
(Intercept)	-3.314	0.416	0.036	-7.974	< .001
Work experience (5-10 years)	0.302	0.510	1.352	0.592	0.554
Work experience (10-15 years)	0.849	0.486	2.337	1.746	0.081
Work experience (15-20 years)	1.023	0.484	2.781	2.115	0.034
Work experience (20-25 years)	0.979	0.496	2.661	1.974	0.048
Work experience (25-30 years)	1.081	0.497	2.946	2.176	0.030
Work experience (≥ 30 years)	-0.385	0.615	0.681	-0.626	0.531

Model summary

Model	Deviance	AIC	BIC	df	X^2	p	McFadden R^2	Nagelkerke R^2	Tjur R^2
H_0	653.6	655.648	660.872	1371					
H_1	635.5	649.472	686.040	1365	18.176	0.006	0.028	0.035	0.033

HADS-A

	Estimate	Standard Error	Odds Ratio	z	p
(Intercept)	-1.508	0.198	0.221	-7.595	< .001
Work experience (5-10 years)	-0.179	0.263	0.836	-0.680	0.496
Work experience (10-15 years)	-0.361	0.281	0.697	-1.284	0.199
Work experience (15-20 years)	-0.164	0.279	0.848	-0.589	0.556
Work experience (20-25 years)	-0.347	0.299	0.707	-1.160	0.246
Work experience (25-30 years)	-0.461	0.315	0.631	-1.461	0.144
Work experience (≥ 30 years)	-1.116	0.341	0.328	-3.276	0.001

Model summary

Model	Deviance	AIC	BIC	df	X^2	p	McFadden R^2	Nagelkerke R^2	Tjur R^2
H_0	1093	1094.619	1099.843	1371					
H_1	1078	1092.140	1128.708	1365	14.479	0.025	0.013	0.019	0.000

For peer review only

STROBE 2007 (v4) Statement—Checklist of items that should be included in reports of *cross-sectional studies*

Section/Topic	Item #	Recommendation	Reported on page #
**Title and abstract**	1	(*a*) Indicate the study's design with a commonly used term in the title or the abstract	1 - 2
		(*b*) Provide in the abstract an informative and balanced summary of what was done and what was found	1 – 2
**Introduction**			
Background/rationale	2	Explain the scientific background and rationale for the investigation being reported	3 – 4
Objectives	3	State specific objectives, including any prespecified hypotheses	4
**Methods**			
Study design	4	Present key elements of study design early in the paper	4 - 5
Setting	5	Describe the setting, locations, and relevant dates, including periods of recruitment, exposure, follow-up, and data collection	4
Participants	6	(*a*) Give the eligibility criteria, and the sources and methods of selection of participants	4
Variables	7	Clearly define all outcomes, exposures, predictors, potential confounders, and effect modifiers. Give diagnostic criteria, if applicable	5
Data sources/ measurement	8*	For each variable of interest, give sources of data and details of methods of assessment (measurement). Describe comparability of assessment methods if there is more than one group	4
Bias	9	Describe any efforts to address potential sources of bias	*5*
Study size	10	Explain how the study size was arrived at	-
Quantitative variables	11	Explain how quantitative variables were handled in the analyses. If applicable, describe which groupings were chosen and why	5
Statistical methods	12	(*a*) Describe all statistical methods, including those used to control for confounding	5-6
		(*b*) Describe any methods used to examine subgroups and interactions	5-6
		(*c*) Explain how missing data were addressed	5-6
		(*d*) If applicable, describe analytical methods taking account of sampling strategy	-
		(*e*) Describe any sensitivity analyses	-
**Results**			

For peer review only

Participants	13*	(a) Report numbers of individuals at each stage of study—eg numbers potentially eligible, examined for eligibility, confirmed eligible, included in the study, completing follow-up, and analysed	7
		(b) Give reasons for non-participation at each stage	7
		(c) Consider use of a flow diagram	-
Descriptive data	14*	(a) Give characteristics of study participants (eg demographic, clinical, social) and information on exposures and potential confounders	7
		(b) Indicate number of participants with missing data for each variable of interest	7
Outcome data	15*	Report numbers of outcome events or summary measures	8-10
Main results	16	(*a*) Give unadjusted estimates and, if applicable, confounder-adjusted estimates and their precision (eg, 95% confidence interval). Make clear which confounders were adjusted for and why they were included	8-10
		(*b*) Report category boundaries when continuous variables were categorized	-
		(*c*) If relevant, consider translating estimates of relative risk into absolute risk for a meaningful time period	-
Other analyses	17	Report other analyses done—eg analyses of subgroups and interactions, and sensitivity analyses	8-10
**Discussion**			
Key results	18	Summarise key results with reference to study objectives	10-12
Limitations	19	Discuss limitations of the study, taking into account sources of potential bias or imprecision. Discuss both direction and magnitude of any potential bias	13
Interpretation	20	Give a cautious overall interpretation of results considering objectives, limitations, multiplicity of analyses, results from similar studies, and other relevant evidence	14
Generalisability	21	Discuss the generalisability (external validity) of the study results	13
**Other information**			
Funding	22	Give the source of funding and the role of the funders for the present study and, if applicable, for the original study on which the present article is based	17

*Give information separately for cases and controls in case-control studies and, if applicable, for exposed and unexposed groups in cohort and cross-sectional studies.

Note: An Explanation and Elaboration article discusses each checklist item and gives methodological background and published examples of transparent reporting. The STROBE checklist is best used in conjunction with this article (freely available on the Web sites of PLoS Medicine at http://www.plosmedicine.org/, Annals of Internal Medicine at http://www.annals.org/, and Epidemiology at http://www.epidem.com/). Information on the STROBE Initiative is available at www.strobe-statement.org.

For peer review only

BMJ Open

Potential traumatic events in the workplace and depression, anxiety and posttraumatic stress: a cross-sectional study among Dutch gynaecologists, paediatricians and orthopaedic surgeons.

Journal:	*BMJ Open*
Manuscript ID	bmjopen-2019-033816.R3
Article Type:	Original research
Date Submitted by the Author:	13-May-2020

Complete List of Authors:	Scheepstra, Karel; Amsterdam UMC - Locatie AMC, Psychiatry<br>Pauw, Hannah; Amsterdam UMC - Locatie AMC, Faculty of Medicine<br>van Steijn, Minouk; Onze Lieve Vrouw Hosp, Obstetrics and Gynaecology; Amsterdam UMC - Locatie AMC, Obstetrics and Gynaecology<br>Stramrood, Claire; OLVG Locatie Oost, Obstetrics and Gynaecology<br>Olff, Miranda; Amsterdam UMC - Locatie AMC, Psychiatry; Arq Psychotrauma Research<br>van Pampus, Maria G.; Onze Lieve Vrouw Hosp, Obstetrics and Gynaecology

**Primary Subject Heading**:	Mental health
Secondary Subject Heading:	Occupational and environmental medicine, Health services research
Keywords:	OCCUPATIONAL & INDUSTRIAL MEDICINE, PSYCHIATRY, Depression & mood disorders < PSYCHIATRY, Anxiety disorders < PSYCHIATRY, Adverse events < THERAPEUTICS, Protocols & guidelines < HEALTH SERVICES ADMINISTRATION & MANAGEMENT

SCHOLARONE™
Manuscripts

I, the Submitting Author has the right to grant and does grant on behalf of all authors of the Work (as defined in the below author licence), an exclusive licence and/or a non-exclusive licence for contributions from authors who are: i) UK Crown employees; ii) where BMJ has agreed a CC-BY licence shall apply, and/or iii) in accordance with the terms applicable for US Federal Government officers or employees acting as part of their official duties; on a worldwide, perpetual, irrevocable, royalty-free basis to BMJ Publishing Group Ltd ("BMJ") its licensees and where the relevant Journal is co-owned by BMJ to the co-owners of the Journal, to publish the Work in this journal and any other BMJ products and to exploit all rights, as set out in our licence.

The Submitting Author accepts and understands that any supply made under these terms is made by BMJ to the Submitting Author unless you are acting as an employee on behalf of your employer or a postgraduate student of an affiliated institution which is paying any applicable article publishing charge ("APC") for Open Access articles. Where the Submitting Author wishes to make the Work available on an Open Access basis (and intends to pay the relevant APC), the terms of reuse of such Open Access shall be governed by a Creative Commons licence – details of these licences and which Creative Commons licence will apply to this Work are set out in our licence referred to above.

Other than as permitted in any relevant BMJ Author's Self Archiving Policies, I confirm this Work has not been accepted for publication elsewhere, is not being considered for publication elsewhere and does not duplicate material already published. I confirm all authors consent to publication of this Work and authorise the granting of this licence.

For peer review only

Potential traumatic events in the workplace and depression, anxiety and posttraumatic stress: a cross-sectional study among Dutch gynaecologists, paediatricians and orthopaedic surgeons.

Authors

Karel W. F. Scheepstra*, MD[1]
Hannah S. Pauw, BSc[2]
Minouk E. van Steijn, MD[3,4]
Claire A.I. Stramrood, MD PhD[4]
Miranda Olff, PhD[1,5]
Maria G. van Pampus, MD PhD[4]

1. Amsterdam UMC, University of Amsterdam, Department of Psychiatry, Meibergdreef 9, 1105 AZ, Amsterdam, the Netherlands.
2. Amsterdam UMC, University of Amsterdam, Faculty of Medicine, Meibergdreef 9, 1105 AZ, Amsterdam, the Netherlands.
3. Amsterdam UMC, University of Amsterdam, Department of Obstetrics and Gynaecology, Meibergdreef 9, 1105 AZ, Amsterdam, the Netherlands.
4. OLVG, Department of Obstetrics and Gynaecology, Oosterpark 9, 1090 HM, Amsterdam, the Netherlands
5. Arq Psychotrauma Expert Group, Nienoord 5, 1112 XE, Diemen, the Netherlands

***Corresponding author:**
K.W.F. Scheepstra
Department of Psychiatry
Meibergdreef 9
1105 AZ Amsterdam
The Netherlands
k.w.scheepstra@amsterdamumc.nl
+31 (0) 20 8913500

For peer review only

ABSTRACT

Objective: To compare the prevalence of work-related potential traumatic events, support protocols and mental health symptoms across Dutch gynaecologists, orthopaedic surgeons and paediatricians.

Design: Cross-sectional study, supplementary analysis of combined data.

Setting: Nationwide survey between 2014 and 2017.

Participants: An online questionnaire was sent to all Dutch gynaecologists, orthopaedic surgeons and paediatricians, including resident physicians (4959 physicians). 1374 questionnaires were eligible for analysis, corresponding with a response rate of 27.7%.

Outcome measures: Primary outcome measures were the prevalence of work-related potential traumatic events (PTEs), depression, anxiety, psychological distress and traumatic stress, measured with validated screening instruments (Hospital Anxiety and Depression Scale, HADS; Trauma Screening Questionnaire, TSQ). Secondary outcomes were the association of mental health and defensive practice to traumatic events and support protocols.

Results: Of the respondents, 20.8% experienced a work-related potential traumatic event at least four weeks ago. Prevalence rates indicative of depression, anxiety or posttraumatic stress disorder (PTSD) were 6.4%, 13.6% and 1.5% respectively. Depression (9.2% vs. 5.2%, p=0.019), anxiety (18.2% vs. 8.2%, p<0.001) and psychological distress (22.8% vs. 12.5%, p<0.001), were significantly more prevalent in female compared to male attendings. The absence of a support protocol was significantly associated with more probable PTSD (p=0.022). Those who witnessed a PTE, reported more defensive work changes (28.0% vs 20.5%, p=0.007) and those with probable PTSD considered to quit medical work more often (60.0% vs. 35.8%, p=0.032).

Conclusion: Physicians are frequently exposed to potential traumatic events with high emotional impact over the course of their career. Lacking a support protocol after adverse events was associated with more posttraumatic stress. Adverse events were associated with considering to quit medical practice and a more defensive practice. More awareness must be created for the mental health of physicians as well as for the implementation of a well-organised support system after potential traumatic events.

Word count: 300

Keywords: Occupational & Industrial Medicine, Psychiatry, Depression & mood disorders, Anxiety disorders, Adverse Events, Protocols & guidelines

ARTICLE SUMMARY

Strengths and limitations of this study

- This is one of the few studies done on PTSD in physicians, in particular with regard to mental health in relation to social and occupational functioning and clinical practice related consequences.
- This study incorporated the A and E criterion in our probable PTSD diagnosis, therefore the prevalence is more likely to reflect the real prevalence of PTSD.

For peer review only - http://bmjopen.bmj.com/site/about/guidelines.xhtml

- One limitation is that the population of this study is limited to three specialties and one country in Europe and thus it is difficult to translate these results to physicians worldwide.

INTRODUCTION

Physicians are frequently exposed to medical adverse events, such as life-threatening situations, illnesses, grief, death, and patient violence and aggression. Dealing with such events makes the medical profession one with occupational health hazards, comparable with fire fighters, rescue workers, military and police personnel.[1–3] The support for physicians is, however, underdeveloped in most areas of the medical profession compared to other professions.[4,5] It is generally assumed that health care providers have adequate coping mechanisms to deal with such work stressors. However, adequate coping mechanisms do not warrant successful coping with severe potential traumatic events. Our previous work showed that most gynaecologists, paediatricians and midwives experience support and protocols after work-related potential traumatic events as insufficient. [6,7] Dutch gynaecologists reported that development of coping strategies with work-related stressors are neither taught during graduate nor in specialist training.[8] Physician health is one of the competencies described in the CanMEDS framework used in Dutch medical education.[9] This framework recognises that, to provide optimal patient care, physicians must take responsibility for their own well-being and that of their colleagues. Several hospitals have started peer-support programmes, as this was shown to help physicians dealing with adverse events.[10]

Depression, anxiety and posttraumatic stress disorder

Major depressive disorder (MDD) is a common affective disorder that is characterised by a depressed mood and/or loss of pleasure.[11] A review on the epidemiology of mental disorders in Europe described a one-year prevalence of 6.9% in the general population[12]; the Nemesis-2 study showed a one-year prevalence of mood disorders of 3.0% among Dutch citizens with a high income.[13] A meta-analysis on depressive symptoms and MDD among resident physicians showed high prevalence rates (20.9% to 43.2%).[14] Fewer studies were done on depressive symptoms among medical

For peer review only - http://bmjopen.bmj.com/site/about/guidelines.xhtml

specialists, showing prevalence rates from 10.3 to 28.0%.[15–18] A recent literature review showed that as many as 40 per 100,000 physicians die by suicide each year in the United States of America, more than double the rate of the general population.[19]

Anxiety disorders include many different disorders, ranging from a specific phobia to a generalised anxiety disorder. The one-year prevalence for anxiety disorders in the Netherlands is 6.0% among citizens with a high income and 10.1% among citizens with a low income.[13]
PTSD is a highly debilitating disorder that can develop after exposure to a psychotraumatic event.[20] Symptoms of PTSD include intrusions, avoidance of stimuli related to the trauma, alterations in cognition or mood, and hyperarousal. The point prevalence of posttraumatic stress symptoms among physicians ranges between 3.8% and 15.0.[15,21–25]

The main objective of this supplementary analysis was to compare the prevalence of potential traumatic events (PTEs) and related mental health disorders between a large group of gynaecologists, paediatricians and orthopaedic surgeons. Additionally, this study investigates potential traumatic events and clinical practice related consequences. Combining the data of previous work allows us to gain statistical power to explore the association between support protocols and mental health symptoms in a larger group.

METHODS

Population and procedure

Each physician received one invitation for an online questionnaire (supplementary file 1) and two reminders over a period of 8 weeks. The questionnaire was sent through Thesistools® or Surveymonkey®, creating an anonymous (non-traceable) e-mail link. The questionnaire was sent to 1589 gynaecologists, 2160 paediatricians and 1210 orthopaedic surgeons, with a total of 4959 physicians, including residents. The invitation to participate in the questionnaire for the gynaecologists was sent in 2014, orthopaedic surgeons in 2016 and paediatricians in 2017. This is an additional analysis of combining data of previous work.

For peer review only - http://bmjopen.bmj.com/site/about/guidelines.xhtml

The membership databases of the Dutch Society of Obstetrics & Gynaecology (NVOG), Dutch Society of Orthopaedic Surgeons (NOV) and the Dutch Society of Paediatricians (NvK) were used for the invitation. Registration as a medical specialist or resident in one of these societies is obligatory and therefore most Dutch medical specialists in these fields were contacted. These databases contained residents, attending, retired and non-practicing physicians.

This study was exempted from ethical approval by the Medical Research Ethics Committees United (MEC-U), and registered under numbers W17.169 and W18.096.

For peer review only

Patient and public involvement

Engagement with the described medical societies was vital to ensure participation and a high response rate. The qualitative questions were piloted among several medical specialists of each specific medical specialty.

Measurements

The instruments included the Hospital Anxiety and Depression Scale (HADS) and the Trauma Screening Questionnaire (TSQ) as validated screening instruments. The HADS is a screening instrument for depression and anxiety with 14 items (4-point likert scale), where the subscales contain seven questions each. The cut-off value of the Dutch version of the depression (HADS-D) and anxiety subscale (HADS-A) is equal to or higher than eight. The total HADS cut-off value for general distress is equal or higher than 12, corresponding to clinically relevant psychological distress; this is a rather non-specific but sensitive measurement to screen for mental disorders.[26] Cut-off scores were used according to these validation studies. The sensitivity and specificity for both HADS-A and HADS-D are approximately 0.80, with Cronbach's alphas for HADS-A from 0.68 to 0.93 (mean 0.83) and for HADS-D from 0.67 to 0.90, in different populations. [27]

For peer review only - http://bmjopen.bmj.com/site/about/guidelines.xhtml

The TSQ is a ten-item screening instrument for a probable diagnosis of PTSD according to the DSM-IV, with a sensitivity of 0.94 and specificity of 0.56 when using a cut-off of equal to six or higher. The Dutch psychometric properties are validated with Cronbach's alphas from 0.71 to 0.91.[28] Only respondents who answered 'yes' to experiencing a work-related traumatic event at least four weeks ago were asked to fill out the TSQ.

Additionally, the survey contained questions about demographics, personal experiences, coping and support strategies after adverse events in the workplace. Regarding the latter, multiple-choice options were given as well as an open field where respondents were able to add their individual answers or comment on their experience. These qualitative questions were piloted among several medical specialists of all three medical specialties, to increase face value of the questions. To quantify the number of PTEs and previous posttraumatic stress symptoms, questions were added based on the criteria of the diagnosis of PTSD in the DSM-IV (questions 22 and 35, supplementary file 1).

Statistical analysis

Statistical analysis was performed using IBM Statistical Package for the Social Sciences (SPSS) version 22 and JASP version 0.8.5.1. Demographic data and multiple-choice questions were analysed using descriptive statistics and exported as frequency tables and bar charts. Differences in outcomes (in various groups) for categorical variables were tested using either a Chi-squared test or a Fisher's exact test where applicable. A two-sided p-value equal to or less than 0.05 was considered statistically significant. A multivariable logistic regression analysis was performed to estimate the effects of age, work-experience, gender and position on the likelihood that participants develop mental health outcomes, a p-value equal to or less than 0.05 was considered statistically significant. Multiple testing corrections were not performed.

For peer review only - http://bmjopen.bmj.com/site/about/guidelines.xhtml

RESULTS

A total of 1374 questionnaires were collected with a response rate of 27.7%. Table 1 shows the respondents characteristics by specialty.

Table 1: Respondent characteristics

	Total (n=4959)	Gynaecologists (n=1589)	Orthopaedic surgeons (n=1210)	Paediatricians (n=2160)
**Response rate**	27.7	48.9	21.3	29.8
**Gender**				
Male	44.9	34.7	85.6	32.7
Female	55.1	65.3	14.4	67.3
**Position**				
Resident	23.0	26.9	20.9	18.0
Attending physician	67.2	64.7	62.3	74.9
Retired	7.1	5.4	13.4	5.6
Non-practicing	2.6	3.0	3.4	1.5
**Years in practice**				
≤ 5	12.4	14.3	12.0	9.8
6-10	18.6	19.0	19.5	17.3
11-15	15.8	17.7	14.0	13.9
16-20	14.3	11.6	13.4	19.3
21-25	12.4	12.8	9.2	13.9
26-30	11.3	9.5	14.4	12.0
>30	15.1	14.7 *	17.5	13.9
**Complaints disciplinary board**	19.9	21.0	29.8	11.2
**Support protocol**				
Yes	19.6	15.8	18.8	26.3
No	42.4	52.7	30.1	34.1
I do not know	38.0	31.4	51.0	39.5

All values shown as %
*=2 missing

The majority of the orthopaedic surgeons were male (85.6%) while in the other specialties the majority were female. In all specialties most of the respondents (62.3 – 74.9%) were attending physicians, as seen in table 1. The gender and position distributions were compared with the membership databases of the societies, and for none of the specialties the distribution in the sample was significantly different from the population. The orthopaedic surgeons reported to have had the most complaints at a disciplinary board (29.8%). One hundred eight (26.3%) paediatricians reported to have a support protocol, whereas only 18.8% of the orthopaedic surgeons and 15.8% of the gynaecologists reported to have a protocol after adverse events.

For peer review only - http://bmjopen.bmj.com/site/about/guidelines.xhtml

Outcomes of the HADS and TSQ by specialty are shown in table 2.

Table 2: Depression, anxiety, psychological distress and PTSD by specialty

	Total (n=1374)	Gynaecologists (n=672)	Orthopaedic Surgeons (n=292)	Paediatricians (n=410)
**Depression**	6.4	6.5	4.8	7.3
**Anxiety**	13.6	15.8	8.2	14.1
**Psychological distress**	17.1	18.2	12.0	19.3
**PTSD**				
PTE ≥ 4 weeks ago	20.8	12.8	19.5	34.9
PTS symptoms earlier	13.4	8.2	8.6	25.4
TSQ	1.5	1.5	0.3	2.2

All values shown in % scored above HADS or TSQ cut off
Abbreviations: Potential Traumatic Event (PTE); Trauma Screening Questionnaire (TSQ); Hospital Anxiety and Depression Scale (HADS); Posttraumatic stress (PTS); Posttraumatic stress disorder (PTSD).

Table 3: Depression, anxiety and psychological distress by position (resident vs. attending) and gender

	Total (n=1240)	Depression	p	Anxiety	p	Psycho-logical distress	p
**Gender**							
Male	40.8	5.5	0.075	10.1	**<0.001**	14.2	**<0.001**
Female	59.2	8.2		18.7		22.3	
**Position**							
Resident	25.4	4.7	0.112	17.1	0.125	18.4	0.887
Attending	74.6	7.4		13.6		18.1	
**Residents**							
Male	25.6	3.7	0.346	13.6	0.053	14.8	0.058
Female	74.4	5.1		18.3		19.6	
**Attending**							
Male	45.9	5.2	**0.008**	8.2	**<0.001**	12.5	**<0.001**
Female	54.1	9.2		18.2		22.8	

All values shown as % scored above cut-off HADS
All differences were analysed using χ^2
Abbreviations: Hospital Anxiety and Depression Scale (HADS)

For peer review only - http://bmjopen.bmj.com/site/about/guidelines.xhtml

Eighty eight (6.4%) respondents scored above the cut-off value for depression and one hundred and eighty eight (13.6%) respondents scored above the cut-off value for anxiety. Two hundred and thirty six (17.1%) scored above the cut-off value of the total HADS, corresponding to clinically relevant psychological distress. When comparing specialties, there is no significant difference in depression. However, looking at the prevalence of anxiety, the orthopaedic surgeons scored significantly lower compared to both gynaecologists (15.8% vs. 8.2%, $\chi 2$,p<0.01) and paediatricians (14.1% vs. 8.2%, $\chi 2$, p<0.05). Also psychological distress was significantly lower compared to gynaecologists (18.2% vs. 12.0%, $\chi 2$, p<0.05) and paediatricians (19.3% vs. 12.0%, $\chi 2$,p<0.01).

As seen in table 3, no significant difference was found between male and female residents in rates of depression, anxiety and psychological distress. When comparing gender among attendings, the rates of depression (5.2% vs. 9.2%, p=0.019), anxiety (8.2% vs. 18.2%, p<0.001) and the total HADS (12.5·0% vs. 22.8%, p<0.001) were significantly higher among female attendings.When comparing among all respondents, the prevalence rates of anxiety (10.1% vs. 18.7%, p<0.001) and psychological distress (14.2% vs. 22.3%, p<0.001) were significantly higher among female physicians There were no differences in primary outcomes when comparing residents with attendings. Turning to the results of the logistic regression analysis, there was a significant increase in the prevalence of depression in the groups with more years in practice (OR 2.95, p=0.030, 95% CI 1.11 – 7.80, supplementary file 2), but not with increased age. We found a significant decrease in the prevalence of anxiety with more years in practice (OR 0.33, p=0.001, 95% CI 0.17 - 0.64, supplementary file 2). The highest rate of respondents with a score above the cut-off value for anxiety were found among physicians with 6-10 years in practice (supplementary file 2).

Posttraumatic stress disorder

Two hundred eighty six respondents (20.8%) reported having experienced at least one potential traumatic event (PTE) during their work, at least four weeks ago, thereby meeting DSM-IV criterion A and E. Of these respondents, 183 reported recognising posttraumatic stress (PTS) symptoms after this event (13.4% of the total group, 64.3% of the potentially traumatised group) and 20 screened positive

For peer review only - http://bmjopen.bmj.com/site/about/guidelines.xhtml

for probable PTSD (1.5% of the total group, 7.0% of the potentially traumatised group, table 2). Of the paediatricians 34.9% reported having experienced a PTE at least four weeks ago. This is significantly higher than the percentages of the gynaecologists (12.8%, $\chi 2$, $p<0.001$) and orthopaedic surgeons (19.5%, $\chi 2$, $p<0.001$), as seen in table 2. When comparing the prevalence of probable PTSD, the paediatricians had a significant higher prevalence compared to the orthopaedic surgeons (2.2% vs. 0.3%, $\chi 2$, $p=0.041$). Both paediatricians and gynaecologists report to recognise more PTS symptoms.

When comparing gender and position (resident vs. attending, table 4), residents are less likely to have experienced a PTE (16.8% vs. 22.6%, p=0.030) and thus also report significantly fewer PTS symptoms earlier (7.9% vs. 15.6%, p<0.001).

Table 4: Posttraumatic stress outcomes by position (resident vs. attending) and gender

	Total (n=1240)	PTE	p	PTS symptoms earlier	p	PTSD	p
**Gender**							
Male	40.8	21.3	0.878	11.3	**0.044**	1.2	0.516
Female	59.2	21.0		15.3		1.6	
**Position**							
Resident	25.4	16.8	**0.030**	7.9	**<0.001**	0.9	0.391
Attending	74.6	22.6		15.6		1.6	
**Residents**							
Male	25.6	21.0	0.245	4.9	0.246	0.0	0.306
Female	74.4	15.3		8.9		1.3	
**Attending**							
Male	45.9	21.4	0.377	12.5	**0.006**	1.4	0.371
Female	54.1	23.6		18.2		1.8	

All values shown as %, PTSD is shown as scored above cut-off TSQ
All differences were analysed using χ^2
Abbreviations: Potential Traumatic Event (PTE); Trauma Screening Questionnaire (TSQ); Hospital Anxiety and Depression Scale (HADS); Posttraumatic stress (PTS); Posttraumatic stress disorder (PTSD).

For peer review only - http://bmjopen.bmj.com/site/about/guidelines.xhtml

The highest rate of probable PTSD was found among attendings (n=15, 1.6%) compared to three of the residents (0.9%). When comparing gender, male and female physicians report the same number of PTEs, but female physicians are more likely to report earlier PTS-symptoms (15.3% vs. 11.3%, p=0.044).

Those who witnessed a PTE, reported significantly more defensive work changes after an incident (28.0% vs 20.5%, p=0.007). Respondents who recognised PTS-symptoms after an incident considered quitting medical work more often (42.6% vs. 29.2%, p=0.029). Those with current probable PTSD significantly considered quitting medical work more often (60.0% vs. 35.8%, p=0.032).
When evaluating the presence of a support protocol, this was found not to influence the primary mental health outcomes, except for probable PTSD. Participants who responded that there was no support protocol at their work-place after an adverse event, were significantly more likely to report probable PTSD after a PTE (p=0.022).

DISCUSSION

Depression and anxiety

This additional analysis combined data from previous work to compare the point prevalence rates of probable depression, anxiety, psychological distress, work-related PTE and PTSD among gynaecologists, orthopaedic surgeons and paediatricians. It is part of the Work-related Adverse and Traumatic Events Research (WATER) and this paper combines the results of previous studies [6,8], to gain statistical power and show differences between support protocols, specialties, gender and position. Our point prevalence rates of depression (6.4%) and anxiety (13.6%), are high compared to 12-month prevalence rates of mood disorders (3.0%) and anxiety (6.0%) in high income populations in the Netherlands, and compared to European 12-month prevalence rate of anxiety (6.4%).[12,13] The point prevalence rates in this study are comparable to American 12-month general prevalence rates of depression (6.7%) and anxiety (18.1%).[29,30] This indicates that physicians are more depressed and anxious compared to the general population and the population with high income in the Netherlands. This high prevalence is in line with current literature, where even higher prevalence rates for

For peer review only - http://bmjopen.bmj.com/site/about/guidelines.xhtml

depression and anxiety were found among medical students, residents and specialists.[14,15,31] One of the relevant findings of this study is the high prevalence of psychological distress (17.6%). Scoring above this cut-off is not specific for one diagnosis, but does indicate a high level of distress and a risk for potential unfit physicians. We found a significant increase in the prevalence of depression corresponding with more years in practice. Furthermore, this study showed a significantly higher prevalence of depression and anxiety among female compared to male attendings. This finding correlates with previous study results,[32] where prevalence rates for depression and anxiety were higher among British female doctors compared to males. These findings are also in line with epidemiologic data on the gender distribution of mood and anxiety disorders in the general population.[12,13] This finding is relevant, because of the feminization of medical specialties globally, and an increase in female physicians of 1.0 to 2.0 % each year in the last ten years in the Netherlands.[33] These results may implicate that a change is needed in support after adverse events, that is sensitive to the increase in women working in the field of medicine.

Posttraumatic stress disorder

In this sample, 285 respondents (20.8%) reported to have experienced a potential traumatic event (PTE) that took place longer than four weeks ago. This is within the range of the estimated prevalence of second victims of 10.4% and 43.3%, reported by Seys et al.[34] Our prevalence for work-related PTSD (1.5%) is slightly higher, compared to the general Dutch prevalence of PTSD (1.2%).[35] The Dutch prevalence of only work-related PTSD is not known, however assumed to be lower. Of the potential traumatised respondents, 7.0% screened positive for PTSD, which is comparable to other occupations with mental health hazards, such as police officers after critical police incidents (7.0%).[36]

One of the key findings of this supplementary analysis, is that after experiencing a PTE, the absence of a support protocol is significantly associated with more probable PTSD (p=0.022). This once again shows the importance of support protocols and implicates that a well perceived support system may prevent posttraumatic stress complaints. This survey did however not explore the types of support

For peer review only - http://bmjopen.bmj.com/site/about/guidelines.xhtml

protocols (e.g. debriefing versus peer-support) in place, as they may differ across hospitals and even within one hospital across specialties. Therefore nothing can be concluded of the type of support protocol that may help prevent PTS symptoms. However, some form of formalised support may lead to seeking- and finding professional help earlier. Studies have shown that the threshold to look for professional mental health as a physician is high. Physicians report a reluctance to seek care, due to barriers such as too little time, fear of losing their medical license and stigma.[37] A (semi) compulsory support protocol after a PTE could potentially increase awareness, lower this threshold and guide physicians in need towards professional support or treatment. Debriefing and hospital based peer-support have shown useful and effective after PTEs in several small studies. [10,38] Also, this awareness of more workplace support may lead to perceiving adverse events less psychotraumatic, however having a support protocol was not correlated with less PTEs.

Of the residents who filled out the TSQ, three screened positive for PTSD (all female respondents). These findings are in line with the known gender distribution, as women are more likely to develop PTSD than men.[39] Other studies found higher prevalence rates of PTSD (i.e. Joseph[21] = 15%, Mills[24] = 11.9%, Ruitenburg[15] =15%, and Shi[22] = 3.8%), but they used different (non-) validated screening instruments and methods. None of these screening instruments assessed when the PTE happened (for a diagnosis of PTSD this needs to be at least four weeks ago), meaning that their respondents may not meet criterion A of PTSD and could also be suffering from acute stress symptoms. Furthermore, these studies did not assess whether the potential event was work-related, so these numbers show general PTSD prevalence rates which are usually higher.
Studies have shown that dysfunctional coping can predict complicated grief and PTSD severity.[40,41] Thus, it is important to educate physicians about coping mechanisms and stress management techniques to prevent adverse mental health conditions.[42] With social support being an important protective factor for PTSD[43], the role of educating health care professionals to address this problem and support their colleagues in peer-support groups is crucial.[10]

For peer review only - http://bmjopen.bmj.com/site/about/guidelines.xhtml

Job related consequences of depression, anxiety, adverse events and PTSD

This study shows that physicians consider quitting their job more often, after having experienced PTS-symptoms in the past or currently suffering from probable PTSD. Experiencing a PTE is also associated with more defensive medical practice. When looking at the prevalence of complaints at a disciplinary board, the orthopaedic surgeons reported the most complaints (table 1). This may be due to the higher amount of elective work they do, which usually leads to more dissatisfied patients, compared to emergency work. More complaints was not associated with considering quitting work or increased rates of depression, anxiety or psychological distress.

Strengths

Little research has been done on PTSD in physicians, in particular with regard to mental health in relation to social and occupational functioning and clinical practice related consequences. We incorporated the A and E criterion in our probable PTSD diagnosis, therefore our prevalence is more likely to reflect the real prevalence of PTSD. Also, this study included a large number (n=1374) of physicians, which made it possible to analyse gender, position and age differences. The overall response rate among gynaecologists was high (42.9%), followed by the orthopaedic surgeons (29%) and the paediatricians (18.9%).

Limitations

There are several limitations to this study. The population of this study is limited to three specialties and one country in Europe and thus it is difficult to translate these results to physicians worldwide. However, it is known that working conditions and related stress levels are comparable with the rest of Europe and North-America. Also, prevalence rates reported in this study are possibly not comparable to other studies on mental health, as many different samples and measurements are used in mental health studies. Furthermore, avoidance is one of the key symptoms of PTSD and can therefore lead to non-response and non-completion of the survey, as it may trigger symptoms of PTSD. Therefore the prevalence rates found in psychotrauma studies are often an underestimation of the PTSD-prevalence. On the other hand, this self-selection bias may also lead to overrepresentation of physicians with

For peer review only - http://bmjopen.bmj.com/site/about/guidelines.xhtml

For peer review only

strong opinions. One suggestion to decrease this non-completion bias, is to give respondents the opportunity to finish the survey at a later time point and receive a reminder. The overall response rate was 27.7%, which is slightly higher than the average response rate for email surveys (25.0%)[44] and comparable to similar surveys in this field [45]. Due to the low numbers of PTSD, it was not possible to correlate the type of adverse event with more PTSD. Furthermore, the HADS and TSQ are validated screening instruments, however they only give an estimate of the prevalence rates of probable disorders as they do not cover all the symptom domains of the disorders. Only the prevalence of PTSD is work-related, the numbers for depression and anxiety are general prevalence rates.

For peer review only

For peer review only - http://bmjopen.bmj.com/site/about/guidelines.xhtml

CONCLUSION

Physicians are often exposed to PTEs over the course of their career with potentially high emotional impact. The prevalence rates for probable depression (6.4%) and anxiety (13.6%) were higher among physicians, compared to the general population with high income in the Netherlands. The point prevalence of clinical psychological distress was 19.0% and of probable PTSD was 1.5%. Female physicians are significantly more prone to develop depressive- and anxiety disorders than their male colleagues. PTEs were associated with more defensive practice and dissatisfaction about work. And importantly, the absence of a support protocol was associated with more probable PTSD. This once again shows the importance of support protocols and implicates that a well perceived support system, such as peer support and debriefings, may prevent posttraumatic stress complaints and unfit physicians.

For peer review only

For peer review only - http://bmjopen.bmj.com/site/about/guidelines.xhtml

ACKNOWLEDGEMENTS

We would like to thank the Dutch Society of Obstetrics & Gynaecology (NVOG), Dutch Society of Orthopaedic Surgeons (NOV), the Dutch Society of Paediatricians (NvK), and their members, for their cooperation in this study.

For peer review only

For peer review only - http://bmjopen.bmj.com/site/about/guidelines.xhtml

COMPETING INTERESTS

None of the authors declare a conflict of interest.

For peer review only

FUNDING

This research received no specific grant from any funding agency in the public, commercial or not-for-profit sectors.

For peer review only

CONTRIBUTORSHIP

KS, MvS, CS, MvP were involved in the design of this study. KS and HP analysed the data and made the first draft. KS, HP, MvS, CS, MO and MvP were involved in finalising the manuscript.

For peer review only - http://bmjopen.bmj.com/site/about/guidelines.xhtml

DATA AVAILABILITY

The data can be made available by the corresponding author upon reasonable request and for a well defined purpose. The request will be discussed in our research group. The data will be de-identified participant data. A statistical analysis plan is available.

For peer review only

REFERENCES

1 Yehuda R, Vermetten E, McFarlane AC, *et al.* PTSD in the military: Special considerations for understanding prevalence, pathophysiology and treatment following deployment. *Eur J Psychotraumatol* Published Online First: 2014. doi:10.3402/ejpt.v5.25322

2 van der Meer CAI, Bakker A, Schrieken BAL, *et al.* Screening for trauma-related symptoms via a smartphone app: The validity of Smart Assessment on your Mobile in referred police officers. *Int J Methods Psychiatr Res* Published Online First: 2017. doi:10.1002/mpr.1579

3 Mellotte H, Murphy D, Rafferty L, *et al.* Pathways into mental health care for UK veterans: a qualitative study. *Eur J Psychotraumatol* Published Online First: 2017. doi:10.1080/20008198.2017.1389207

4 Edrees H, Federico F. Supporting clinicians after medical error. *BMJ* 2015. doi:10.1136/bmj.h1982

5 Devi S. Doctors in distress. *Lancet* Published Online First: 2011. doi:10.1016/S0140-6736(11)60145-1

6 van Steijn ME, Scheepstra KWF, Yasar G, *et al.* Occupational well-being in pediatricians—a survey about work-related posttraumatic stress, depression, and anxiety. *Eur J Pediatr* Published Online First: 2019. doi:10.1007/s00431-019-03334-7

7 Kerkman T, Dijksman LM, Baas MAM, *et al.* Traumatic Experiences and the Midwifery Profession: A Cross-Sectional Study Among Dutch Midwives. *J Midwifery Women's Heal* Published Online First: 2019. doi:10.1111/jmwh.12946

8 Baas MAM, Scheepstra KWF, Stramrood CAI, *et al.* Work-related adverse events leaving their mark: A cross-sectional study among Dutch gynecologists. *BMC Psychiatry* 2018;**18**. doi:10.1186/s12888-018-1659-1

9 Frank JR, Snell L, Sherbino J E. CanMEDs 2015 Physician Competency Framework. CanMEDS 2015 Physician Competency Fram. Ottawa R. Coll. Physicians Surg. Canada. 2015;:1–30.

10 Hu YY, Fix ML, Hevelone ND, *et al.* Physicians' needs in coping with emotional stressors: The case for peer support. *Arch Surg* Published Online First: 2012. doi:10.1001/archsurg.2011.312

11 American Psychiatric Association. Diagnostic and Statistical Manual of Mental Disorders: DSM-5. *Am Psychiatr Assoc* 2013;:991. doi:10.1176/appi.books.9780890425596.744053

12 Alonso J, Angermeyer MC, Bernert S, *et al.* Prevalence of mental disorders in Europe: results from the European Study of the Epidemiology of Mental Disorders (ESEMeD) project. *Acta Psychiatr Scand Suppl* 2004;**109**:21–7. doi:10.1111/j.1600-0047.2004.00327.x

13 De Graaf R, Ten Have M, Van Gool C, *et al.* Prevalence of mental disorders and trends from 1996 to 2009. Results from the Netherlands Mental Health Survey and Incidence Study-2. Soc. Psychiatry Psychiatr. Epidemiol. 2012;**47**:203–13. doi:10.1007/s00127-010-0334-8

14 Mata DA, Ramos MA, Bansal N, *et al.* Prevalence of Depression and Depressive Symptoms Among Resident Physicians: A Systematic Review and Meta-analysis. *JAMA* 2015;**314**:2373–83. doi:10.1001/jama.2015.15845

For peer review only

For peer review only - http://bmjopen.bmj.com/site/about/guidelines.xhtml

15 Ruitenburg MM, Frings-Dresen MH, Sluiter JK. The prevalence of common mental disorders among hospital physicians and their association with self-reported work ability: a cross-sectional study. *BMC Health Serv Res* 2012;**12**:292. doi:10.1186/1472-6963-12-292

16 Wurm W, Vogel K, Holl A, *et al.* Depression-burnout overlap in physicians. *PLoS One* Published Online First: 2016. doi:10.1371/journal.pone.0149913

17 Hsu K, Marshall V. Prevalence of depression and distress in a large sample of Canadian residents, interns, and fellows. *Am J Psychiatry* Published Online First: 1987. doi:10.1176/ajp.144.12.1561

18 Tomioka K, Morita N, Saeki K, *et al.* Working hours, occupational stress and depression among physicians. *Occup Med (Chic Ill)* Published Online First: 2011. doi:10.1093/occmed/kqr004

19 O T, A A, D T, *et al.* Physician Suicide: A Silent Epidemic. In: *In: APA annual meeting, Poster no 227, New York City*. 2018. 123–4.

20 Kessler RC, Aguilar-Gaxiola S, Alonso J, *et al.* Trauma and PTSD in the WHO World Mental Health Surveys. Eur. J. Psychotraumatol. 2017. doi:10.1080/20008198.2017.1353383

21 Joseph B, Pandit V, Hadeed G, *et al.* Unveiling posttraumatic stress disorder in trauma surgeons. *J Trauma Acute Care Surg* 2014;**77**:148–54. doi:10.1097/TA.0000000000000271

22 Shi L, Wang L, Jia X, *et al.* Prevalence and correlates of symptoms of post-traumatic stress disorder among Chinese healthcare workers exposed to physical violence: a cross-sectional study. *BMJ Open* 2017;**7**:e016810. doi:10.1136/bmjopen-2017-016810

23 Chan AOM, Huak CY. Influence of work environment on emotional health in a health care setting. *Occup Med (Lond)* 2004;**54**:207–12. doi:10.1093/occmed/kqh062

24 Mills LD, Mills TJ. Symptoms of post-traumatic stress disorder among emergency medicine residents. J. Emerg. Med. 2005. doi:10.1016/j.jemermed.2004.05.009

25 Wahlberg, Andreen Sachs M, Johannesson K, *et al.* Post-traumatic stress symptoms in Swedish obstetricians and midwives after severe obstetric events: a cross-sectional retrospective survey. *BJOG An Int J Obstet Gynaecol* Published Online First: 2017. doi:10.1111/1471-0528.14259

26 Spinhoven P, Ormel J, Sloekers PP, *et al.* A validation study of the Hospital Anxiety and Depression Scale (HADS) in different groups of Dutch subjects. *Psychol Med* 1997;**27**:363–70. doi:10.1017/S0033291796004382

27 Bjelland I, Dahl AA, Haug TT, *et al.* The validity of the Hospital Anxiety and Depression Scale. An updated literature review. *J Psychosom Res* 2002.

28 Dekkers AMM, Olff M, Maring GWB. Identifying persons at risk for PTSD after trauma with TSQ in the Netherlands. *Community Ment Health J* 2010;**46**:20–5. doi:10.1007/s10597-009-9195-6

29 Kessler RC, Chiu WT, Demler O, *et al.* Prevalence, severity, and comorbidity of 12-month DSM-IV disorders in the National Comorbidity Survey Replication. *Arch Gen Psychiatry* 2005;**62**:617–27. doi:10.1001/archpsyc.62.6.617

30 Substance Abuse and Mental Health Services Administration, Center for Behavioural Health Statistics and Quality. Results from the 2015 National Survey on Drug Use and Health: Detailed Tables,. 2015.

31 Hope V, Henderson M. Medical student depression, anxiety and distress outside North

For peer review only - http://bmjopen.bmj.com/site/about/guidelines.xhtml

America: a systematic review. *Med Educ* 2014;**48**:963–79. doi:10.1111/medu.12512

32 Wall TD, Bolden RI, Borrill CS, *et al.* Minor psychiatric disorder in NHS trust staff: Occupational and gender differences. *Br J Psychiatry* 1997;**171**:519–23. doi:10.1192/bjp.171.6.519

33 Capaciteitsorgaan. Capaciteitsplan 2016 (in Dutch). Utrecht: 2016. www.capaciteitsorgaan.nl accessed 2-7-2018

34 Seys D, Wu AW, Gerven E Van, *et al.* Health Care Professionals as Second Victims after Adverse Events. *Eval Health Prof* 2013;**36**:135–62. doi:10.1177/0163278712458918

35 De Vries GJ, Olff M. The lifetime prevalence of traumatic events and posttraumatic stress disorder in the Netherlands. *J Trauma Stress* Published Online First: 2009. doi:10.1002/jts.20429

36 Carlier I V, Lamberts RD, Gersons BP. Risk factors for posttraumatic stress symptomatology in police officers: a prospective analysis. *J Nerv Ment Dis* Published Online First: 1997. doi:10.1097/00005053-199708000-00004

37 Dyrbye LN, West CP, Sinsky CA, *et al.* Medical Licensure Questions and Physician Reluctance to Seek Care for Mental Health Conditions. *Mayo Clin Proc* Published Online First: 2017. doi:10.1016/j.mayocp.2017.06.020

38 Baverstock A, Finlay F. Specialist registrars' emotional responses to a patient's death. *Arch Dis Child* Published Online First: 2006. doi:10.1136/adc.2005.076760

39 Olff M, Langeland W, Draijer N, *et al.* Gender differences in posttraumatic stress disorder. [Review] [287 refs]. *Psychol Bull* Published Online First: 2007. doi:10.1037/0033-2909.133.2.183

40 Schnider KR, Elhai JD, Gray MJ. Coping style use predicts posttraumatic stress and complicated grief symptom severity among college students reporting a traumatic loss. *J Couns Psychol* 2007;**54**:344–50. doi:10.1037/0022-0167.54.3.344

41 Kerai SM, Khan UR, Islam M, *et al.* Post-traumatic stress disorder and its predictors in emergency medical service personnel: a cross-sectional study from Karachi, Pakistan. *BMC Emerg Med* 2017;**17**:26. doi:10.1016/j.brat.2003.07.010

42 Brosschot JF. Ever at the ready for events that never happen. *Eur J Psychotraumatol* Published Online First: 2017. doi:10.1080/20008198.2017.1309934

43 Olff M. Bonding after trauma: On the role of social support and the oxytocin system in traumatic stress. *Eur J Psychotraumatol* Published Online First: 2012. doi:10.3402/ejpt.v3i0.18597

44 Fluid Surveys University. Response Rate Statistics for Online Surveys - What Numbers Should You be Aiming For? 2014.

45 Shanafelt TD, Boone S, Tan L, *et al.* Burnout and satisfaction with work-life balance among US physicians relative to the general US population. *Arch Intern Med* Published Online First: 2012. doi:10.1001/archinternmed.2012.3199

For peer review only

For peer review only

Supplementary files

1. Questionnaire
2. Supplementary table: logistic regression work experience vs. HADS-D and HADS-A

For peer review only

For peer review only - http://bmjopen.bmj.com/site/about/guidelines.xhtml

Supplementary file 1: Questionnaires

Paediatricians

1 What is your gender?
1. Male
2. Female

2 What is your age?
1. 20-29 years
2. 30-39 years
3. 40-49 years
4. 50-59 years
5. 60-69 years
6. ≥ 70 years

3 Are you a member of the Dutch Society of Paediatricians (NvK)?
1. Yes
2. No

4 What is your current position?
1. Resident
2. Attending physician
3. Retired
4. Non-practicing/management

5 How many years have you been working in the paediatrics field as a physician? (Including as intern)
1. ≤ 5 years
2. 6-10 years
3. 11-15 years
4. 16-20 years
5. 21-25 years
6. 26-30 years
7. > 30 years

6 At the moment, are you working in a general hospital or in an academic referral centre?
1. General hospital
2. Academic referral centre
3. Both
4. I do not work anymore, but I used to work in a general hospital
5. I do not work anymore, but I used to work in an academic referral centre

7 At which department do you work the most? (If you are not practicing anymore, fill in where you used to work)
1. Neonatology
2. Emergency room
3. General paediatrician
4. NICU
5. Child intensive care
6. Policlinic
7. Children's oncology
8. Other…

8 What do you consider to be an adverse event on the work-floor? (More than one answer possible)
1. Not applicable

For peer review only

2. Bad news conversation/interview
3. (Critically) ill neonate
4. (Critically) ill children
5. When a patient dies
6. When you miss a diagnosis
7. When you have to refuse a patient
8. When you are in doubt whether you are making the right decision
9. When an inexperienced colleague is on call
10. When you decide on an abstaining course of treatment
11. Treatment of neonates
12. When you know a patient will remain severely handicapped
13. Handling the emotions of the parents
14. (Suspicion of) child abuse
15. Other …

9 How do you cope with adverse events on the work-floor? (More than one answer possible)

1. Not applicable / never experienced
2. Seeking professional help
3. Use new medication
4. Drink (more) alcohol
5. Smoke (more) cigarettes
6. Use (more) drugs
7. Work out (more)
8. Going home as soon as possible
9. Call in sick
10. Hide your emotions
11. Find a distraction
12. Praying or other religious activities
13. Talking to friends and/or family
14. Informally discussing the matter with peers/colleagues
15. Quitting
16. Work less
17. Not working any evening/night shifts
18. Other …

10 The current support organized by your institution after an adverse event is good:

1. Strongly disagree
2. Disagree
3. Agree
4. Strongly agree

11 There is plenty of room to informally discuss adverse events in the department/partnership:

1. Strongly disagree
2. Disagree
3. Agree
4. Strongly agree

12 There is plenty of room to informally discuss adverse events in the hospital:

1. Strongly disagree
2. Disagree
3. Agree
4. Strongly agree

13 There should be a culture change regarding support after an adverse event:

1. Strongly disagree

For peer review only

2. Disagree
3. Agree
4. Strongly agree

14 There is a protocol at your institution regarding support after an adverse event:
1. Yes
2. Yes, but nobody uses it
3. No, but there is one currently being created
4. No
5. I do not know

15 Support after an adverse event consisted of: (more than one answer possible)
1. I have never experienced an adverse event
2. There was none
3. Professionally organised peer-support
4. Self-initiated peer-support with direct colleagues (own department)
5. Self-initiated peer-support with indirect colleagues (different department)
6. Conversation(s) with a psychologist or coach
7. Evaluation with the present team
8. Help from the medical officer after a sick-leave
9. Other …

16 Your preferred support after an adverse event would be: (more than one answer possible)
1. Not applicable
2. Support is unnecessary
3. Professionally organised peer-support
4. Evaluation with the present team
5. Peer-support from direct colleagues (own department)
6. Peer-support from indirect colleagues (different department)
7. Mindfulness
8. Other …

17 It should be mandatory for the hospital to organise support after an adverse event:
1. Yes
2. No

18 You have learned to cope with adverse events through/during:
1. Med-school (without clerkships)
2. Clerkships
3. Internships
4. Residency
5. Attending physician
6. Other study
7. Psychological help
8. Specific course/training
9. Peer-review
10. Mindfulness
11. Never learnt
12. Other…

19 In the course of your career, you have become more defensive:
1. Strongly disagree
2. Disagree
3. Agree
4. Strongly agree

For peer review only

20 Have you changed your working-conditions (e.g. less shifts, more diagnostic tests) after experiencing a patient-related adverse event?

1. Yes
2. No

21 What did you change? (More than one answer possible)

1. Work less
2. Less evening/night-shifts
3. No evening/night-shifts anymore
4. Calling a colleague sooner
5. Quit my job
6. More diagnostic tests
7. Start treatment sooner
8. Other…

22 Sometimes people experience traumatic events, such as live-threatening situations as a cause of a natural disaster, high-impact trauma or fire; being attacked or raped; witness a murder, death or hear find out someone close to them experienced something terrible. As a physician, one can experience such events in patient-care: critical illness or death of a patients, severe injury, as well as violent behaviour from a patient or their family.
Have you ever, during your work AS A PHYSICIAN experienced such (adverse) events/incidents?

1. Yes
2. No

23 Can you briefly describe the adverse event(s)?

1. Yes, please describe it…
2. No

24 Did (some part of) this/these adverse event(s) take place more than 4 weeks ago?

1. Yes
2. No

Followed by the TSQ questionnaire.

35 It might be that you did not experience the reactions and emotions as described on the previous pages, but that you recognize them from a past period in your life. For example, having disturbing thoughts or dreams, reliving the event, getting upset. Do you recognize these reactions?

1. Yes
2. No

36 Which of these reactions do you recognize? (More than one answer possible)

1. Disturbing thoughts
2. Disturbing dreams
3. Reliving experiences
4. Getting upset
5. Physical responses
6. Insomnia
7. Irritability
8. Trouble focussing
9. Hyper arousal
10. Tensed

37 How long did these symptoms last?

1. < 4 weeks

For peer review only

2. > 4 weeks
3. > 6 months
4. > 1 year

38 Have you ever been diagnosed with PTSD (posttraumatic stress-disorder)?

1. Yes
2. No

Followed by the HADS questionnaire.

53 Have you ever seriously considered quitting your job as a paediatrician? (e.g. by looking for a different job, talking to human resources about ending your contract?

1. Yes, absolutely
2. Yes, sometimes
3. No, never

54 What was the reason to consider quitting? (More than one answer possible)

1. Bad collaboration (working together) with a co-worker
2. Not challenging enough within the field
3. A lot of complications
4. Afraid to make mistakes
5. Too much responsibility
6. Traumatic experience on the work-floor
7. Complaint(s) from patients
8. One-sided
9. A lot of stress
10. A high workload
11. Disbalance between work and private life
12. Disagreements with work providers
13. Different interests
14. New challenge
15. Too much administration
16. Too much bureaucracy
17. Patient-violence
18. Too many rules
19. Insufficient guidance from supervisors
20. Problems with the partnership
21. Private reasons
22. Disutility
23. Other…

55 Did you ever faced a complaint at the disciplinary board?

1. Yes
2. No

Gynaecologists

1 Gender

1. Female
2. Male

2 Age

1. 25-34 years
2. 35-44 years

For peer review only

3. 45-54 years
4. 55-64 years
5. 65 years and older

3 Current position
1. Resident
2. Attending / fellow
3. Retired gynaecologist
4. Non-practicing/management

4 How many years have you been working as a gynaecologist? (Including working as a resident)

5 I considered quitting my job as a gynaecologist:
1. Strongly disagree
2. Disagree
3. Agree
4. Strongly agree

6 In case you considered quitting, what was the most important reason?
1. Most important reason: …
2. Never considered quitting

7 Did you ever face a complaint at the disciplinary board?
1. Yes
2. No

8 What do you consider to be an adverse event on the work-floor? (More than one answer possible)
1. Not applicable
2. Bad news conversations/interview
3. The critical moments when a patient's life is at risk
4. When a patient dies
5. When you miss a diagnosis
6. When you feel like you cannot help the patients
7. When you are in doubt whether you are making the right decision
8. Other …

9 After experiencing adverse events on the work-floor, I do the following: (More than one answer possible)
1. Not applicable
2. Seeking professional help
3. Going home as soon as possible
4. Drink more alcohol, use more drugs and smoke more cigarettes than I normally would do
5. Use new medication I barely or normally not use
6. Trying not to think about it
7. Find a distraction
8. Praying or other religious activities
9. Formal case review
10. Work out or doing my hobby
11. Talking to friends and/or family
12. Informally discussing the matter with peers/colleagues
13. I developed symptoms of a burn-out (e.g. emotional exhaustion, depersonalisation, lower self-esteem)
14. Call in sick
15. Quit my job
16. Other …

For peer review only

10 The current support organized by your institution after an adverse event is good:
1. Strongly disagree
2. Disagree
3. Agree
4. Strongly agree

11 There is plenty of room to informally discuss adverse events in the department/partnership:
1. Strongly disagree
2. Disagree
3. Agree
4. Strongly agree

12 You have learned to cope with adverse events through/during:
1. Clerkships
2. Residency
3. Specific course/training
4. Intervision
5. Other …
6. Never really learned it

13 After experiencing an adverse event I changed my working conditions (e.g. no more shifts, no more vaginal breech births, not performing any surgeries alone, coming to the hospital sooner instead of supervision over the telephone)
1. Strongly disagree
2. Disagree
3. Agree
4. Strongly agree

14 In the course of your career, you have become more defensive:
1. Strongly disagree
2. Disagree
3. Agree
4. Strongly agree

15 Support after an adverse event consisted of… (fill in the box)

16 There is a protocol at your institution regarding support after an adverse event:
1. No
2. I do not know
3. Yes, consisting of…

17 Your preferred support after an adverse event would be: (more than one answer possible)
1. Evaluation with direct colleagues
2. Intervision with indirect colleague's (physicians with a different specialty)
3. Talking to a psychologist or coach
4. A buddy
5. Other …

18 When you lay awake at night, you are not made for this job:
1. Strongly disagree
2. Disagree
3. Agree
4. Strongly agree

For peer review only

19 There should be a culture change regarding support after an adverse event:

1. Strongly disagree
2. Disagree
3. Agree
4. Strongly agree

20 Sometimes people experience traumatic events, such as live-threatening situations as a cause of a natural disaster, high-impact trauma or fire; being attacked or raped; witness a murder, death or hear find out someone close to them experienced something terrible. As a physician, one can experience such events in patient-care: critical illness or death of a patients, severe injury, as well as violent behaviour from a patient or their family.
1) Have you ever, during your work AS A PHYSICIAN experienced such (adverse) events/incidents?
2) After at least one of those adverse events, did you experience intense fear, helplessness or abomination?
3) Did this/these event(s) happened at least 4 weeks ago?

1. I answered yes to all of these questions
2. No, I did not answer yes to all these questions

Followed by the TSQ

31 Can you briefly describe the adverse event(s)?

32 It might be that you did not experience the reactions and emotions as described on the previous pages, but that you recognize them from a past period in your life. For example, having disturbing thoughts or dreams, reliving the event, getting upset. Do you recognize these reactions?

1. Yes
2. No

Followed by the HADS.

Othopaedic surgeons

1 What is your gender?

1. Male
2. Female

2 What is your age?

1. 20-29 years
2. 30-39 years
3. 40-49 years
4. 50-59 years
5. 60-69 years
6. ≥ 70 years

3 Are you a member of the Dutch Orthopaedic Society (NOV)?

1. Yes
2. No

4 What is your current position?

1. Resident
2. Attending physician
3. Retired
4. Non-practicing/management

For peer review only

5 How many years have you been working in the orthopaedic field as a physician? (Including as intern)

1. ≤ 5 years
2. 6-10 years
3. 11-15 years
4. 16-20 years
5. 21-25 years
6. 26-30 years
7. > 30 years

6 What do you consider to be an adverse event on the work-floor? (More than one answer possible)

1. Not applicable
2. Bad news conversation/interview
3. (Critically) ill patients
4. When a patient dies
5. When you miss a diagnosis
6. When you have to refuse a patient
7. When you are in doubt whether you are making the right decision
8. When an inexperienced colleague is on call
9. When you decide on an abstaining course of treatment
10. Treatment of young patients
11. When you know a patient will remain severely handicapped
12. Other …

7 How do you cope with adverse events on the work-floor? (More than one answer possible)

1. Not applicable / never experienced
2. Seeking professional help
3. Use new medication
4. Drink (more) alcohol
5. Smoke (more) cigarettes
6. Use (more) drugs
7. Work out (more)
8. Going home as soon as possible
9. Call in sick
10. Hide your emotions
11. Find a distraction
12. Praying or other religious activities
13. Talking to friends and/or family
14. Informally discussing the matter with peers/colleagues
15. Quitting
16. Work less
17. Other …

8 The current support organized by your institution after an adverse event is good:

1. Strongly disagree
2. Disagree
3. Agree
4. Strongly agree

9 There is plenty of room to informally discuss adverse events in the department/partnership:

1. Strongly disagree
2. Disagree
3. Agree
4. Strongly agree

For peer review only

10 There is plenty of room to informally discuss adverse events in the hospital:
1. Strongly disagree
2. Disagree
3. Agree
4. Strongly agree

11 There should be a culture change regarding support after an adverse event:
1. Strongly disagree
2. Disagree
3. Agree
4. Strongly agree

12 There is a protocol at your institution regarding support after an adverse event:
1. Yes
2. Yes, but nobody uses it
3. No, but there is one currently being created
4. No
5. I do not know

13 Support after an adverse event consisted of: (more than one answer possible)
1. I have never experienced an adverse event
2. There was none
3. Professionally organised peer-support
4. Self-initiated peer-support with direct colleagues (own department)
5. Self-initiated peer-support with indirect colleagues (different department)
6. Conversation(s) with a psychologist or coach
7. Evaluation with the present team
8. Help from the medical officer after a sick-leave
9. Other …

14 Your preferred support after an adverse event would be: (more than one answer possible)
1. Not applicable
2. Support is unnecessary
3. Professionally organised peer-support
4. Evaluation with the present team
5. Peer-support from direct colleagues (own department)
6. Peer-support from indirect colleagues (different department)
7. Mindfulness
8. Other …

15 It should be mandatory for the hospital to organise support after an adverse event:
1. Yes
2. No

16 You have learned to cope with adverse events through/during:
1. Med-school (without clerkships)
2. Clerkships
3. Internships
4. Residency
5. Attending physician
6. Psychological help
7. Specific course/training
8. Peer-review
9. Mindfulness
10. Never learnt

For peer review only

17 In the course of your career, you have become more defensive:
1. Strongly disagree
2. Disagree
3. Agree
4. Strongly agree

18 Have you changed your working-conditions (e.g. less shifts, more diagnostic tests) after experiencing a patient-related adverse event?
1. Yes
2. No

19 What did you change? (More than one answer possible)
1. Work less
2. Less evening/night-shifts
3. No evening/night-shifts anymore
4. Calling a colleague sooner
5. Quit
6. Start treatment sooner
7. Other…

20 Have you ever been diagnosed with PTSD (posttraumatic stress-disorder)?
1. Yes
2. No

21 Sometimes people experience traumatic events, such as live-threatening situations as a cause of a natural disaster, high-impact trauma or fire; being attacked or raped; witness a murder, death or hear find out someone close to them experienced something terrible. As a physician, one can experience such events in patient-care: critical illness or death of a patients, severe injury, as well as violent behaviour from a patient or their family.
Have you ever, during your work AS A PHYSICIAN experienced such (adverse) events/incidents?
1. Yes
2. No

22 Did this/these take place less than 4 weeks ago?
1. Yes
2. No

23 Can you describe the event/incident?
1. Yes
2. No

Followed by the TSQ and HADS questionnaire.

51 Have you ever seriously considered quitting your job as an orthopaedic surgeon? (e.g. by looking for a different job, talking to human resources about ending your contract?
1. Yes
2. No

52 What was the reason to consider quitting? (More than one answer possible)
1. Bad collaboration (working together) with a co-worker
2. Not challenging enough within the field
3. A lot of complication
4. Afraid to make mistakes
5. Too much responsibility

For peer review only

6. Traumatic experience on the work-floor
7. Complaint(s) from patients
8. One-sided
9. A lot of stress
10. A high workload
11. Disbalance between work and private life
12. Disagreements with work providers
13. Different interests
14. New challenge
15. Too much administration
16. Too much bureaucracy
17. Patient-violence
18. Too many rules
19. Insufficient guidance from supervisors
20. Problems with the partnership
21. Private reasons
22. Disutility
23. Other…

53 Did you ever face a complaint at the disciplinary board?
1. Yes
2. No

For peer review only

Supplementary file 2: logistic regression model and odds ratios ‘work experience – HADS-D and HADS-A’

Model summary HADSA

Model	Deviance	AIC	BIC	df	X²	p	McFadden R^2	Nagelkerke R^2	Tjur R^2	Cox & Snell R^2
H_0	1092.619	1094.619	1099.84	1371						
H_1	1078.140	1092.140	1128.70	1365	14.479	0.025	0.013	0.019	0.014	0.010

Coefficients

Work experience	Estimate	Standard Error	Odds Ratio	z	Wald Test: Wald Statistic	Wald Test: df	Wald Test: p	95% Confidence interval (odds ratio scale): Lower bound	95% Confidence interval (odds ratio scale): Upper bound
(Intercept)	-1.508	0.198	0.221	-7.595	57.690	1	< .001	0.150	0.327
5-10 years	-0.179	0.263	0.836	-0.680	0.463	1	0.496	0.500	1.400
10-15 years	-0.361	0.281	0.697	-1.284	1.650	1	0.199	0.401	1.209
15-20 years	-0.164	0.279	0.848	-0.589	0.347	1	0.556	0.491	1.465
20-25 years	-0.347	0.299	0.707	-1.160	1.345	1	0.246	0.393	1.271
25-30 years	-0.461	0.315	0.631	-1.461	2.134	1	0.144	0.340	1.170
>30 years	-1.116	0.341	0.328	-3.276	10.735	1	0.001	0.168	0.639

Note. HADSA level 'Abnormal' coded as class 1.

Model summary HADSD

Model	Deviance	AIC	BIC	df	X²	p	McFadden R^2	Nagelkerke R^2	Tjur R^2	Cox & Snell R^2
H_0	653.648	655.648	660.872	1371						
H_1	635.472	649.472	686.040	1365	18.176	0.006	0.028	0.035	0.033	0.013

Coefficients

	Estimate	Standard Error	Odds Ratio	z	Wald Test: Wald Statistic	Wald Test: df	Wald Test: p	95% Confidence interval (odds ratio scale): Lower bound	95% Confidence interval (odds ratio scale): Upper bound
(Intercept)	-3.314	0.416	0.036	-7.974	63.591	1	< .001	0.016	0.082
5-10 years	0.302	0.510	1.352	0.592	0.350	1	0.554	0.498	3.675
10-15 years	0.849	0.486	2.337	1.746	3.048	1	0.081	0.901	6.064
15-20 years	1.023	0.484	2.781	2.115	4.472	1	0.034	1.078	7.176
20-25 years	0.979	0.496	2.661	1.974	3.897	1	0.048	1.007	7.033
25-30 years	1.081	0.497	2.946	2.176	4.736	1	0.030	1.113	7.797
>30 years	-0.385	0.615	0.681	-0.626	0.392	1	0.531	0.204	2.270

Note. HADSD level 'Abnormal' coded as class 1.

For peer review only

STROBE 2007 (v4) Statement—Checklist of items that should be included in reports of *cross-sectional studies*

Section/Topic	Item #	Recommendation	Reported on page #
**Title and abstract**	1	(*a*) Indicate the study's design with a commonly used term in the title or the abstract	1 - 2
		(*b*) Provide in the abstract an informative and balanced summary of what was done and what was found	1 – 2
**Introduction**			
Background/rationale	2	Explain the scientific background and rationale for the investigation being reported	3 – 4
Objectives	3	State specific objectives, including any prespecified hypotheses	4
**Methods**			
Study design	4	Present key elements of study design early in the paper	4 - 5
Setting	5	Describe the setting, locations, and relevant dates, including periods of recruitment, exposure, follow-up, and data collection	4
Participants	6	(*a*) Give the eligibility criteria, and the sources and methods of selection of participants	4
Variables	7	Clearly define all outcomes, exposures, predictors, potential confounders, and effect modifiers. Give diagnostic criteria, if applicable	5
Data sources/ measurement	8*	For each variable of interest, give sources of data and details of methods of assessment (measurement). Describe comparability of assessment methods if there is more than one group	4
Bias	9	Describe any efforts to address potential sources of bias	*5*
Study size	10	Explain how the study size was arrived at	-
Quantitative variables	11	Explain how quantitative variables were handled in the analyses. If applicable, describe which groupings were chosen and why	5
Statistical methods	12	(*a*) Describe all statistical methods, including those used to control for confounding	5-6
		(*b*) Describe any methods used to examine subgroups and interactions	5-6
		(*c*) Explain how missing data were addressed	5-6
		(*d*) If applicable, describe analytical methods taking account of sampling strategy	-
		(*e*) Describe any sensitivity analyses	-
**Results**			

For peer review only

Participants	13*	(a) Report numbers of individuals at each stage of study—eg numbers potentially eligible, examined for eligibility, confirmed eligible, included in the study, completing follow-up, and analysed	7
		(b) Give reasons for non-participation at each stage	7
		(c) Consider use of a flow diagram	-
Descriptive data	14*	(a) Give characteristics of study participants (eg demographic, clinical, social) and information on exposures and potential confounders	7
		(b) Indicate number of participants with missing data for each variable of interest	7
Outcome data	15*	Report numbers of outcome events or summary measures	8-10
Main results	16	(*a*) Give unadjusted estimates and, if applicable, confounder-adjusted estimates and their precision (eg, 95% confidence interval). Make clear which confounders were adjusted for and why they were included	8-10
		(*b*) Report category boundaries when continuous variables were categorized	-
		(*c*) If relevant, consider translating estimates of relative risk into absolute risk for a meaningful time period	-
Other analyses	17	Report other analyses done—eg analyses of subgroups and interactions, and sensitivity analyses	8-10
**Discussion**			
Key results	18	Summarise key results with reference to study objectives	10-12
Limitations	19	Discuss limitations of the study, taking into account sources of potential bias or imprecision. Discuss both direction and magnitude of any potential bias	13
Interpretation	20	Give a cautious overall interpretation of results considering objectives, limitations, multiplicity of analyses, results from similar studies, and other relevant evidence	14
Generalisability	21	Discuss the generalisability (external validity) of the study results	13
**Other information**			
Funding	22	Give the source of funding and the role of the funders for the present study and, if applicable, for the original study on which the present article is based	17

*Give information separately for cases and controls in case-control studies and, if applicable, for exposed and unexposed groups in cohort and cross-sectional studies.

Note: An Explanation and Elaboration article discusses each checklist item and gives methodological background and published examples of transparent reporting. The STROBE checklist is best used in conjunction with this article (freely available on the Web sites of PLoS Medicine at http://www.plosmedicine.org/, Annals of Internal Medicine at http://www.annals.org/, and Epidemiology at http://www.epidem.com/). Information on the STROBE Initiative is available at www.strobe-statement.org.

For peer review only
